# Click-Enabled Grafting for Adaptive Chiral Recognition in Porous Crystals

**DOI:** 10.1021/jacs.5c17377

**Published:** 2026-01-07

**Authors:** Guillermo Gómez-Tenés, Alechania Misturini, Neyvis Almora-Barrios, Sergio Tatay, Natalia M. Padial, Carlos Martí-Gastaldo

**Affiliations:** Functional Inorganic Materials Team, Instituto de Ciencia Molecular (ICMol), 16781 Universitat de València, Paterna 46980, Spain

## Abstract

Reticular frameworks are promising candidates for chiral environments, yet most rely on static stereogenic units, overlooking adaptive host–guest interactions in enantioselective recognition. We report a modular postsynthetic click strategy to install amino acid-derived peptidic moieties into UiO-68 frameworks without compromising crystallinity. Only the histidine-functionalized material exhibits high enantioselectivity for cetirizine. Simulations reveal adaptive interaction pockets, emphasizing the importance of local pore reorganization in chiral molecular recognition.

## Graphical abstract

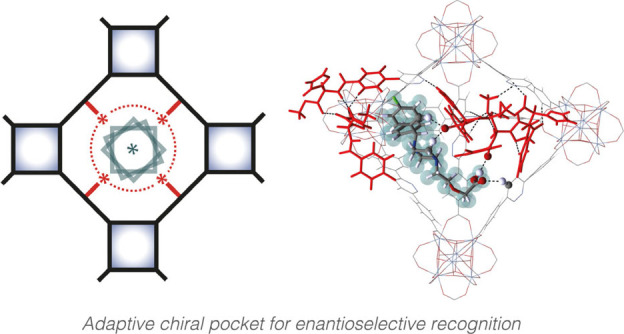

The modularity of reticular frameworks^1^ makes them ideal for engineering chiral recognition sites.^2–4^ Many studies show that incorporating chiral units into Metal–Organic or Covalent Organic Frameworks (MOFs and COFs) can endow them with enantioselective properties;^5–13^ however they often treat chirality as sufficient for molecular discrimination, overlooking a key feature of natural recognition: adaptivity. In biology, enantioselectivity arises not only from chirality but from dynamic environments that adapt to molecular inputs.^14^ Inspired by this paradigm, we hypothesized that coupling permanent chiral functionalities with adaptive behavior in confined pores could offer an alternative approach for chiral discrimination (Figure 1).

**Figure 1:**
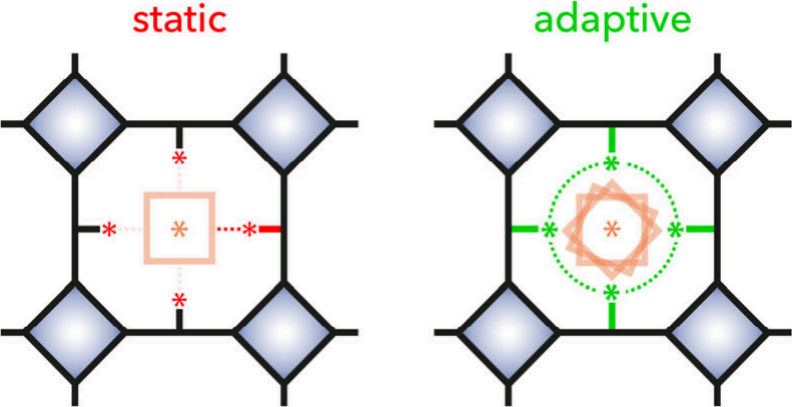
Static vs adaptive chiral pore environments. In a static cavity (*left*), fixed stereogenic elements enable enantioselective recognition through predefined interactions, but cannot respond to guest features. An adaptive cavity (*right*) reorganizes its local environment in response to guest uptake.

Amino acids are ideal candidates for this purpose. Beyond their intrinsic stereochemistry, their diverse side chains enable multiple noncovalent interactions with guests.^2–4^ When connected through peptide bonds, these residues can organize into conformationally dynamic arrays that offer chirality, directionality, and adaptivity, thereby reinforcing enantiodiscrimination through cooperative effects.^15^ Various strategies have been developed to incorporate amino acids into MOFs. Direct synthesis from enantiopure amino acid–based linkers can be used to introduce chirality during framework formation,^16^ but often suffers from limited crystallinity and structural disorder due to the steric and polar nature of amino acid side chains. Moreover, it is often incompatible with the assembly of certain frameworks, reducing its general applicability. Alternatively, postsynthetic modification (PSM) offers a more versatile strategy,^17^ enabling the grafting of amino acids onto preassembled frameworks either through coordination to the metal nodes,^18^ or via covalent functionalization of organic linkers bearing reactive tags.^19^ These approaches have expanded the structural and functional diversity of MOFs,^20–22^ allowing the construction of customized chiral environments. Despite these advances, most PSM protocols require harsh conditions, multistep procedures, or reactive intermediates that can compromise framework stability or result in incomplete and heterogeneous functionalization. This makes the construction of homogeneous chiral domains via PSM particularly challenging, especially with complex peptide chemistry.

In this context, we envisioned that tetrazine-based click chemistry,^23^ and specifically the inverse electron-demand Diels–Alder (iEDDA) reaction,^24^ could provide a versatile and chemoselective route for postsynthetic functionalization. Our previous work with UiO-68-TZDC crystals (TZDC = 4,4′-(1,2,4,5-tetrazine-3,6diyl)­dibenzoate) (Figure 2A) demonstrated that this reaction proceeds under mild conditions and enables quantitative linker conversion.^25,26^ By coupling this strategy with a modular platform for the design of silyl enol ether dienophiles bearing enantiopure amino acid cores, we achieved quantitative grafting of short peptide fragments under mild conditions, with minimal impact on crystal integrity. This enabled the preparation of a set of chiral MOFs with chemically distinct side chains, to probe the role of local environment in enantioselective recognition.

**Figure 2:**
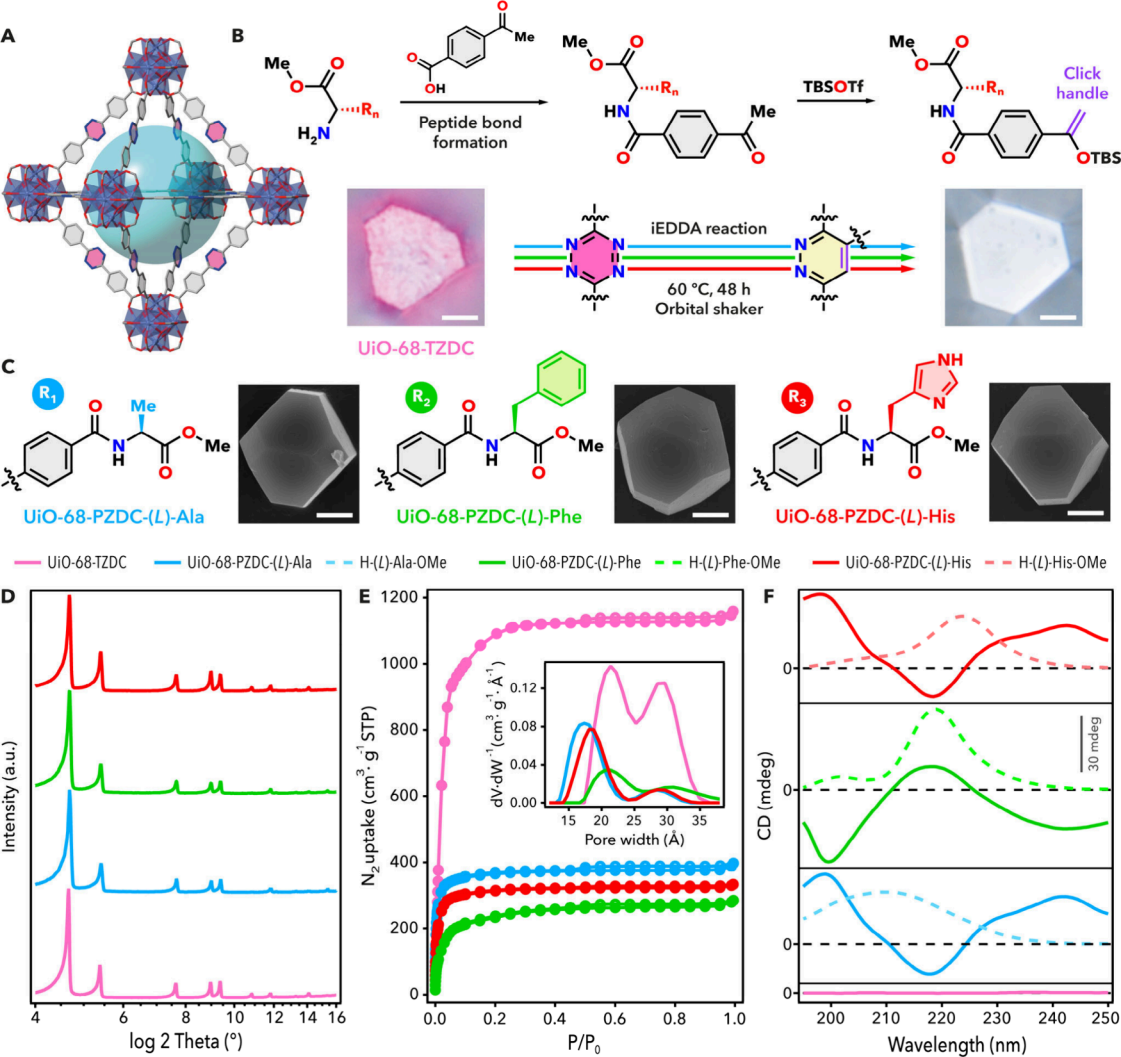
(a) Octahedral cavity of UiO-68-TZDC. (b) Synthesis of enantiopure amino acid dienophiles (*top*) and their subsequent use in the click functionalization of the framework (*bottom*), with optical images of crystals before and after modification (scale bar = 2 μm). (c) Structures of grafted fragments and SEM images of the functionalized crystals. (d) PXRD patterns confirm preservation of crystallinity after functionalization. (e) N_2_ isotherms and PSD analysis (*inset*) confirm the impact of grafting in porosity. (f) Circular dichroism comparison between 1 mg·mL^–1^ material suspensions in methanol and 0.25 mg·mL^–1^ starting amino acid solutions.

To integrate chiral functionalization with iEDDA reactivity, we selected a modular platform for synthesizing clickable dienophiles derived from commercially available *C*-protected amino acids. As illustrated in Figure 2B, the route involves forming a peptidic bond between the amino acid and 4-acetylbenzoic acid using a coupling agent, followed by installation of a silyl enol ether at the ketone position. This tag enables quantitative cycloaddition to *s*-tetrazines under mild conditions, as reported previously.^27^ Using this two-step strategy, we synthesized dienophiles from (*L*)-alanine (Ala), (*L*)-phenylalanine (Phe), and (*L*)-histidine (His), selected for their ability to establish distinct noncovalent interactions via their side chains (Figure 2C). The compounds were obtained in good yields and retained the stereochemistry and side-chain functionality of the parent amino acid (Supplementary Section S3). The *C*-terminus protecting group was deliberately retained to prevent interference from the free carboxylate in guest recognition.

Crystals of UiO-68-TZDC were prepared following our previously reported procedure (Supplementary Section S2).^28^ Click functionalization was done in anhydrous methanol by adding 20 equiv. of the amino acid-derived dienophile (Supplementary Section S4). The reaction mixture was incubated at 60 °C for 48 h under gentle orbital shaking, which better preserved crystal integrity compared to conventional stirring. Progress of the iEDDA reaction was visually confirmed by the fading of tetrazine’s characteristic pink color, observable under an optical microscope (Figure 2B), due to its conversion into the corresponding pyridazine ligand (PZDC). Scanning electron microscopy (SEM) images showed that all functionalized materials retained an average crystal size of ∼ 6 μm and preserved the characteristic truncated octahedral morphology of the parent MOF (Figure 2C). Covalent linkage was confirmed by ^1^H NMR of acid-digested UiO-68-PZDC-(*L*)-X (X = Ala, Phe, His) crystals, showing the expected signals for the functionalized PZDC ligands and negligible contributions from unreacted TZDC (Supplementary Section S5.5). Quantitative analysis using fumaric acid as internal standard revealed >90% conversion for all samples, confirming near-quantitative functionalization of the crystals. HRMS confirmed the exact masses of the covalently functionalized PZDC-(*L*)-X ligands (Supplementary Section S5.6).

Powder X-ray diffraction (PXRD) confirmed the retention of crystallinity, with no loss of long-range order (Figure 2D). Le Bail refinement revealed only minor variations in unit cell parameters (*Fm–3m*, *a* = 32.55 ± 0.01 Å), consistent with preservation of the framework structure despite near-quantitative functionalization (Supplementary Section S5.1). N_2_ adsorption at 77 K supported the maintenance of porosity in UiO-68-PZDC-(*L*)-X, with total pore volumes decreasing from 1.74 cm^3^·g^–1^ for the parent TZDC framework to values between 0.58 (Ala) and 0.41 cm^3^·g^–1^ (Phe) due to the incorporation of side chains of varying size (Figure 2E). Pore size distributions (PSDs) revealed that this pore volume reduction, of down to 76.4%, was dominated by loss of the larger mesoporous cavities. Chiral induction was confirmed by circular dichroism (CD) spectra of the MOFs in methanol (Supplementary Section S5.8). While the parent TZDC framework is CD-silent across the 190–250 nm range, the spectra of UiO-68-PZDC-(*L*)-X show distinct Cotton effects, chiroptical activity and transfer of the chirality from the grafted moieties (Figure 2F). The CD profiles differ clearly from those of the free enantiopure amino acids in position, intensity, and sign. These differences suggest that the observed optical activity is influenced by the spatial arrangement of chiral centers. Ala and His show opposite-sign CD splitting, not observed in the free ligands, indicative of exciton coupling.^29,30^ This implies supramolecular chromophore organization enforced by cavity constraints, amplifying chiroptical response. The fact that such response is observed in bulk crystalline samples with >90% functionalization confirms that the introduced chirality is not limited to surface-accessible sites but homogeneously distributed throughout the crystal volume.

Building on the successful functionalization of the materials, we explored their use for enantioselective guest recognition. Kim and co-workers first demonstrated that MOFs could be used as chiral adsorbents in 2000.^5^ Since then, many studies have expanded this concept,^31^ particularly for small chiral molecules.^10,32–37^ More recent examples have targeted larger compounds, including pharmaceutical drugs.^6,9,11,12,38^ However, most rely on a fixed chiral environment, built into the structure or added postsynthetically, to achieve enantiodiscrimination across racemates. This “one chiral platform–many guests” can work for simple cases but may lack the specificity required to recognize more complex, conformationally flexible drugs. In our case, different chiral selectors are anchored onto a fixed platform, localizing adaptive recognition sites within comparable spatial environments. Their chemical variety and conformational flexibility, enabled by single-peptide bond rotations, may promote specific interactions with one enantiomer. As a benchmark, we selected cetirizine (CTZ), a second-generation antihistamine (Figure 3A). CTZ is difficult to separate because due to its conformational flexibility and polarity. It contains multiple rotatable bonds and functional groups capable of noncovalent interactions, allowing a range of conformations in solution. To the best of our knowledge, no chiral adsorbent has been reported for CTZ separation. Enantioselective recognition was studied by immersing evacuated UiO-68-PZDC-(*L*)-X crystals in racemates of CTZ and shaking at controlled temperatures to promote uptake. After 16 h, enantiomeric excess (*ee*) values were determined by chiral HPLC analysis of the supernatants (Supplementary Section S6).

**Figure 3:**
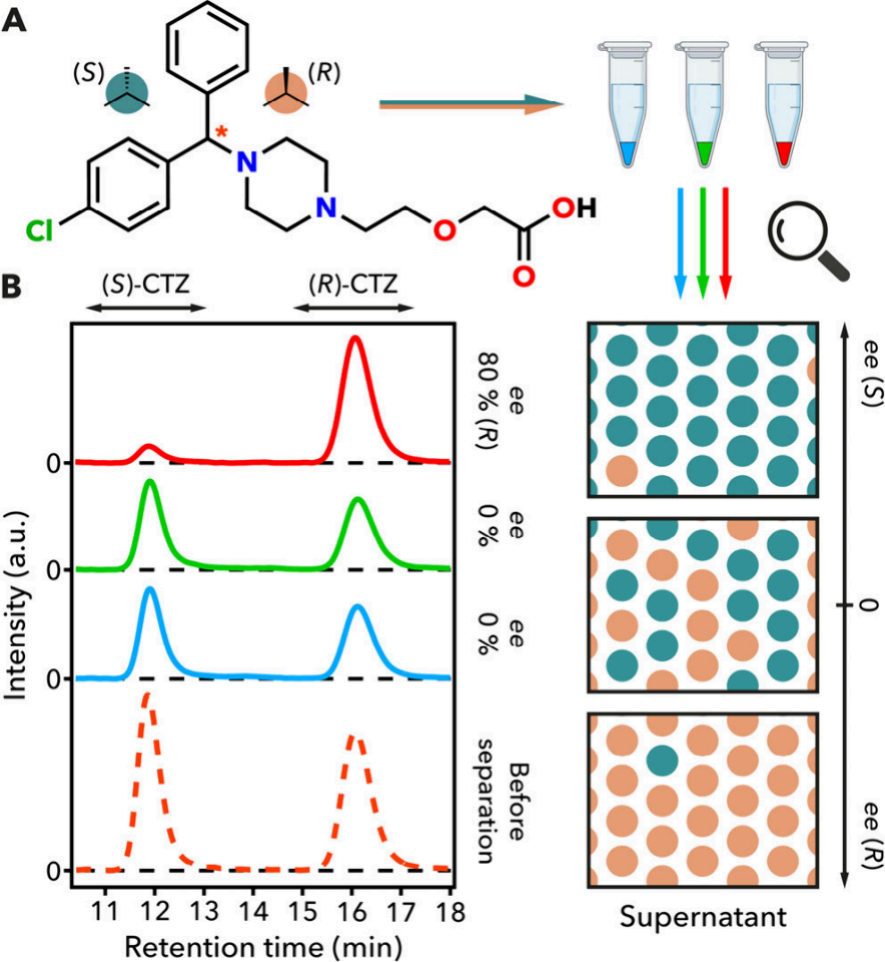
(a) Schematic representation of the side chain effect study of the materials on the enantioselective adsorption of cetirizine. (b) HPLC chromatograms of the cetirizine solutions before and after separation for each MOF.

We optimized the conditions for UiO-68-(*L*)-His, the most enantioselective adsorbent. Ethanol was used to maintain CTZ in its neutral (nonzwitterionic) form and to avoid pH-related artifacts. Enantioselectivity was affected by drug concentration (0.4–2 mM) and temperature (10–35 °C) (Supplementary Section S6.1). Maximum enantioselectivity was observed at intermediate concentration (1 mM) and temperature (25 °C), suggesting a balance between diffusion kinetics and stereoselective binding. Under these conditions, the supernatant reached 80% *ee* for (*R*)-CTZ, confirming preferential uptake of (*S*)-CTZ (Figure 3B), with 83% *ee* measured inside the MOF cavities. While not directly comparable to results obtained using chromatographic or membrane-based methods, these values rank among the highest reported for direct adsorption of chiral drugs by MOFs (Supplementary Section S6.5). In contrast, Ala- and Phe-functionalized MOFs showed *ee* values near zero. Additional experiments with other chiral drugs such as ibuprofen, metoprolol and propranolol (Supplementary Section S6.7) showed negligible enantioselectivity, highlighting the high specificity of the adaptive His-based pockets toward CTZ. The unmodified UiO-68-TZDC showed no enantioselectivity (Supplementary Section S6.2). PXRD and SEM confirmed that encapsulation preserved crystallinity and morphology. A leaching test under guest encapsulation conditions confirmed that the grafted moieties remain covalently anchored to the framework, with no detectable release into solution (Supplementary Section S6.2). To assess recyclability, UiO-68-(*L*)-His was subjected to three consecutive uptake–release cycles, with methanol washes between runs. The material maintained *ee* values >75% across cycles, with no detectable structural degradation (Supplementary Section S6.3). Adsorption capacities confirm that pore occupation by the grafted amino acid fragments reduces the total uptake capacity, while enantioselective adsorption is only observed for the His-functionalized framework (Supplementary Section S.6.6).

To understand the role of the amino acid side chain in enantioselective recognition, we performed *in silico* studies (Supplementary Section S7). Given the cubic symmetry of UiO-68 and the conformational flexibility of the grafted chains, structural refinement beyond the backbone is not feasible, and all models were based on the symmetry and cell parameters obtained from experimental PXRD. Geometry-optimized models of the functionalized frameworks were first obtained using density functional theory (DFT). Host–guest models were generated by adsorption site sampling and DFT optimization. Configurational sampling was required to capture enantioselectivity. We explored the conformational landscape using molecular dynamics (MD) simulations with the partially polarizable pGFN-FF force field.^39,40^ 90 geometries per enantiomer were extracted and energy-minimized. Simulated annealing was used to refine the sampling. The 45 lowest-energy structures per system were used to compute the average interaction energy ( $\overset{®}{\Delta E^{int}}$ ). The difference between the average values for S- and R-cetirizine provided the relative interaction energy, $\Delta \overset{®}{\Delta E^{int}}$ = $\overset{®}{\Delta E^{int}}$ ­(*S*) - $\overset{®}{\Delta E^{int}}$ ­(*R*).

Among the lowest-energy configurations for each system (Figure 4A), (*S*)-CTZ preferentially localizes at the interface between octahedral and tetrahedral cages, where confinement enhances dispersive interactions, particularly with less bulky amino acids like Ala or His. In contrast, frameworks functionalized with Phe accommodate both enantiomers within octahedral cages, likely due to steric hindrance from the phenyl side chain. In all cases, the (*S*) enantiomer exhibits stronger interactions (Figure 4B), with a relative energy difference of at least −3.1 kcal·mol^–1^ for Phe. For the His-functionalized MOF, this difference increases to −12.2 kcal·mol^–1^, suggesting an enthalpic origin for the enantioselectivity. These values are consistent with stabilization energies reported for other histidine-functionalized frameworks, such as MOF-808-His^12^ or Cu-GlyHisGly,^6^ which range from 12 to 14 kcal·mol^–1^ (Table S11).

**Figure 4:**
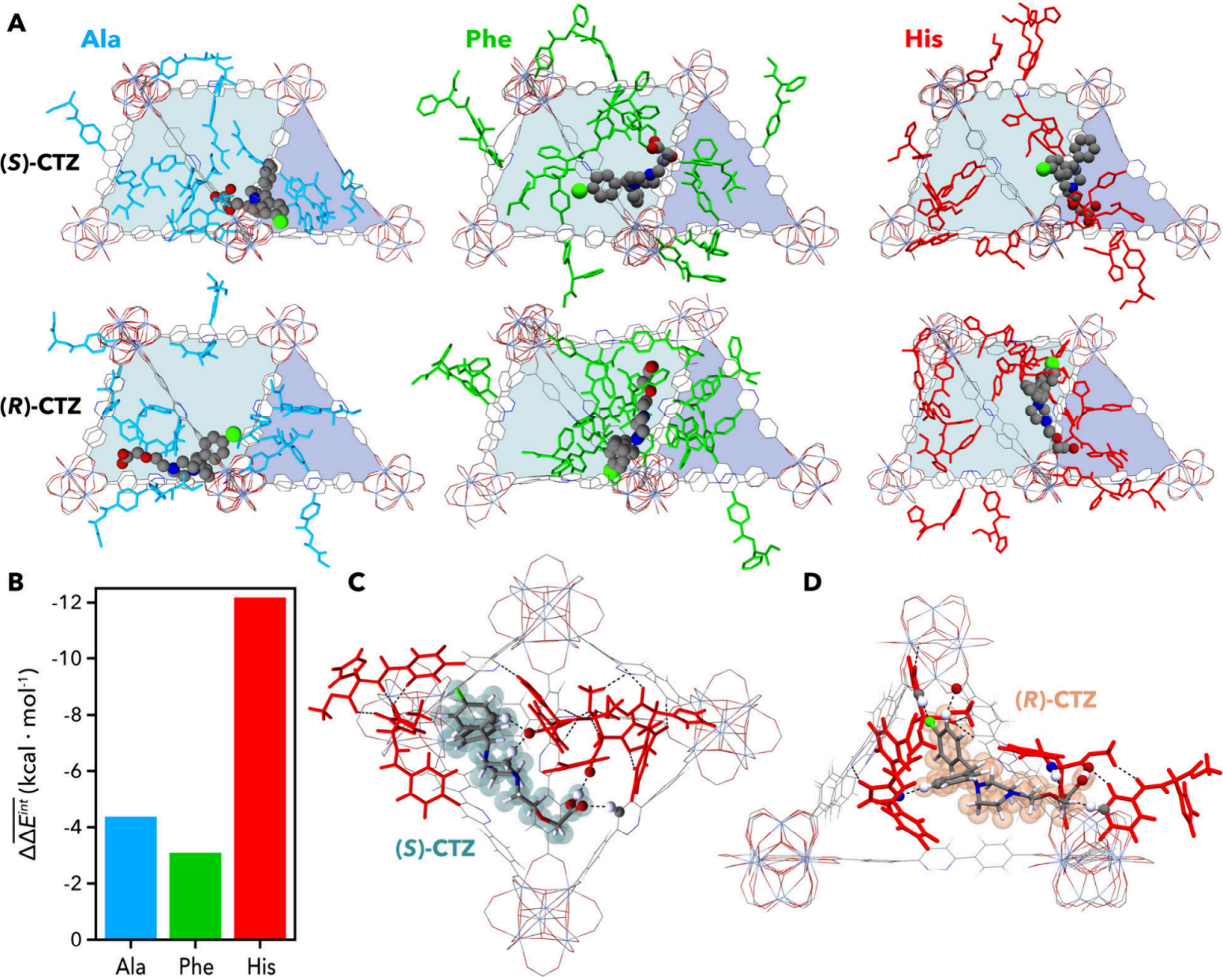
(a) Representative low-energy configurations of (*S*)- and (*R*)-cetirizine within the peptide-functionalized frameworks. Octahedral and tetrahedral cages of UiO-68 are shown in light blue and purple, respectively. (b) Relative interaction energies ( $\overset{®}{\Delta \Delta E^{int}}$ ) between (*S*)- and (*R*)-stereoisomers of CTZ. (c, d) Most stable conformations of UiO-68-(*L*)-His with (*S*)-CTZ (c) and (*R*)-CTZ (d). Hydrogen bonds are shown as dashed lines; donor and acceptor atoms involved in intermolecular hydrogen bonds are represented as spheres.

In the most stable configurations of CTZ within UiO-68-PZDC-(*L*)-His, moderate hydrogen bonds are formed between the framework and the guest. The (*S*) enantiomer primarily acts as a hydrogen-bond donor (Figure 4C), while (*R*)-CTZ establishes more interactions, acting as both donor and acceptor (Figure 4D). A detailed analysis of noncovalent interactions (NCI) is provided in Supplementary Section S7.3. Overall, (*R*)-CTZ is stabilized by moderate hydrogen bonding and weak contacts localized on one face of the molecule, an arrangement also reflected in the NCI surfaces (Movie S1). In contrast, (*S*)-CTZ embeds within a pocket-like environment formed by peptidic moieties, enhancing dispersive interactions (Movie S2). On average, this pocket establishes 5.4 close contacts (≤3.2 Å) with (*S*)-CTZ versus 4.3 for (*R*)-CTZ (Figure S45). Stabilization is further reinforced by intraframework π-π interactions within the pockets. In the case of (*S*)-CTZ, the pocket reconfigures to maximize T-shaped contacts between aromatic units for an average of 12.1 interactions, compared to 8.0 for the (*R*) enantiomer (Table S13). These results suggest that enantioselective recognition arises not only from chirality, but from a locally adaptive environment whose geometry and interaction pattern reorganize to complement the guest’s stereochemistry.

In summary, we have demonstrated that combining modular postsynthetic functionalization with amino acid–derived dienophiles provide a versatile route to engineer adaptive chiral environments within porous frameworks. While all materials shared a common scaffold and level of functionalization, only the histidine-modified framework showed high enantioselectivity for cetirizine adsorption. Experimental evidence, supported by molecular simulations, reveals that this selectivity originates not only from the presence of stereogenic elements, but from the ability of the grafted groups to reorganize locally and form confined interaction pockets tailored to the geometry and functionality of a specific enantiomer. This highlights the importance of adaptivity as a design parameter for chiral porous materials.

By mimicking recognition mechanisms found in biological systems, where selectivity emerges from conformational flexibility in confined molecular spaces, new directions open toward programmable enantioselective systems based on reticular platforms. Moreover, the compatibility of this click-based postfunctionalization strategy with the structural integrity of porous crystals further enables the design of increasingly complex pore environments, potentially incorporating single or multivariate polypeptide sequences to achieve sequence-dependent molecular recognition.

## Supplementary Material







## Notes

General considerations, synthesis and characterization of the starting material, synthesis and characterization of the dienophiles, click functionalization of the materials, characterization of the modified materials, chiral drug separations, computational methods, and NMR spectra (PDF)
NCI surfaces of (*R*)-CTZ at UiO-68-PZDC-(*L*)-His (MP4)
NCI surfaces of (*S*)-CTZ at UiO-68-PZDC-(*L*)-His (MP4)

## ‡

Departamento de Química Física, Universitat de València, Carrer del Doctor Moliner, 50, 46100 Burjassot, Spain

## †

Departamento de Química Inorgánica, Universidad de Granada, Avenida Fuentenueva S/N, Granada, Spain.
