# Supplementary material for: Click-Enabled Grafting for Adaptive Chiral Recognition in Porous Crystals
Source: J Am Chem Soc. 2026 Jan 7;148(2):2096–102. doi: 10.1021/jacs.5c17377 (PMC12833797; doi:10.1021/jacs.5c17377)
Supplement: Supplementary file 1 [file ja5c17377_si_001.pdf]

Supporting Information

Click-Enabled Grafting for Adaptive Chiral Recognition in Porous Crystals

Guillermo Gómez-Tenés, Alechania Misturini, Neyvis Almora-Barrios, Sergio Tatay, Natalia M. Padial* and Carlos Martí-Gastaldo*

*natalia.munoz@uv.es

*carlos.marti@uv.es

TABLE OF CONTENTS

S.1. GENERAL CONSIDERATIONS: STARTING MATERIALS AND CHARACTERIZATION.. 3

S.1.1. MATERIALS AND REAGENTS ... 3
S.1.2. PHYSICAL AND CHEMICAL CHARACTERIZATION ... 4

S.2. SYNTHESIS AND CHARACTERIZATION OF THE STARTING MATERIAL ... 6

S.2.1. SYNTHESIS OF THE STARTING MATERIAL ... 6
S.2.2. CHARACTERIZATION OF THE STARTING MATERIAL ... 7

S.3. SYNTHESIS AND CHARACTERIZATION OF THE DIENOPHILES ... 9

S.3.1. SYNTHESIS OF THE DIENOPHILE PRECURSORS ... 9
S.3.2. SYNTHESIS OF THE DIENOPHILES ... 12

S.4. CLICK FUNCTIONALIZATION OF THE MATERIALS ... 15

S.5. CHARACTERIZATION OF THE MODIFIED MATERIALS ... 16

S.5.1. LE BAIL REFINEMENTS ... 16
S.5.2. OPTICAL IMAGES ... 18
S.5.3. SCANNING ELECTRON MICROSCOPY ... 20
S.5.4. ANALYSIS OF N_2 ISOTHERMS AT 77 K ... 22
S.5.5. ^{1}H NMR ANALYSIS ... 28
S.5.6. HRMS ANALYSIS ... 30
S.5.7. THERMOGRAVIMETRIC ANALYSIS (TGA) ... 31
S.5.8. CIRCULAR DICHROISM (CD) ... 33

S.6. CHIRAL DRUG SEPARATIONS ... 37

S.6.1. OPTIMIZATION OF THE METHODOLOGY ... 37
S.6.2. COMPARISON BETWEEN MATERIALS ... 38
S.6.3. RECYCLABILITY TESTS ... 41
S.6.4. HPLC ANALYSIS ... 43
S.6.5. COMPARISON WITH OTHER CHIRAL MOFs ... 46
S.6.6. ANALYSIS OF THE ADSORPTION CAPACITIES ... 48
S.6.7. TESTING THE MATERIALS WITH OTHER CHIRAL DRUGS ... 49

S.7. COMPUTATIONAL METHODS ... 52

S.7.1. FRAMEWORK MODELS ... 52
S.7.2. INTERACTION ENERGIES ... 53
S.7.3. ANALYSIS OF HOST-GUEST INTERACTIONS ... 58

S.8. NMR SPECTRA ... 62

S.8. REFERENCES ... 77

S.1. GENERAL CONSIDERATIONS: STARTING MATERIALS AND CHARACTERIZATION

S.1.1. MATERIALS AND REAGENTS

All purchased reagents and solvents were used without any previous purification unless specified. Commercially available chemicals were obtained from Sigma-Aldrich, TCI Chemicals, BLD Pharmatech, Scharlab and Eurisotop. Zirconium (IV) chloride (anhydrous, >99.99%), trifluoroacetic acid (99%), sodium nitrite (99%), hydrazine monohydrate (98%), triethylamine (≥99.5%), *tert*-butyldimethylsilyl trifluoromethanesulfonate (98%), cetirizine dihydrochloride (≥98%), propranolol hydrochloride (≥99%) and metoprolol tartrate salt (≥98%) were purchased from Sigma-Aldrich. 4-Cyanobenzoic acid (99%) and ibuprofen were purchased from TCI Chemicals. *N*-Acetyl-*L*-cysteine (98%), 4-acetylbenzoic acid (97%), 1-ethyl-3-(3-dimethylaminopropyl) carbodiimide (99%), (*L*)-alanine methyl ester hydrochloride (97%), (*L*)-phenylalanine methyl ester hydrochloride (99.9%), (*L*)-histidine methyl ester dihydrochloride (98%) and (*R*)-cetirizine dihydrochloride were purchased from BLD Pharmatech. 1-Hydroxybenzotriazole hydrate (98%) was purchased from Manchester Organics. *N,N*-Dimethylformamide (HPLC grade), acetone (HPLC grade), methanol (HPLC grade), ethanol (HPLC grade), *n*-hexane (HPLC grade), ethyl acetate (HPLC grade), dichloromethane (HPLC grade), acetic acid glacial (≥99%) and hydrogen peroxide 30% were purchased from Scharlab. Chloroform-*d* (99.80%), acetone-d_6 and dimethylsulfoxide-d_6 (99.80%) were purchased from Eurisotop.

Analytical thin layer chromatography (TLC) was performed on plates using TLC Silica gel 60 F_{254} from Sigma-Aldrich. Visualization of the compounds was accomplished with short-waved (254 nm) UV light.

S.1.2. PHYSICAL AND CHEMICAL CHARACTERIZATION

Routine X-Ray Diffraction patterns were collected in a PANanlytical X'Pert PRO diffractometer using cooper radiation (CuKα= 1.5418 Å) with an X'Celerator detector, operating at 40 mA and 45 kV. Profiles were collected in the 3º < 2θ < 40º range with a step size of 0.017º.

Profile fitting powder X-ray diffraction patterns were collected using 1.00 mm glass capillaries. The capillaries were mounted and aligned in a PANalytical Empyrean diffractometer using copper radiation (CuKα= 1.5418 Å) with a PIXcel detector, operating at 40 mA and 45 kV. Profiles were collected by using a Soller slit of 0.02º and a divergence slit of 0.25 at room temperature in the angular range 2º < 2θ < 40º with a step size of 0.017º. LeBail profile fittings were carried out with the TOPAS Academic v6 software package (http://www.topas-academic.net/).[1]

Optical images were acquired with a NIKON Eclipse LV150N Optical Microscope with polarized light and Differential Interference Contrast/Nomarski. Plan Fluor objectives with magnification of 20x and 100x were used. The images were taken with a NIKON DS-F/3 camera equipped in the setup.

Scanning electron microscopy (SEM) for testing particle morphologies and dimensions were studied with a Hitachi S-4800 scanning electron microscope at an acceleration voltage of 20 kV, over metalized samples with a mixture of gold and palladium for 60 seconds.

Gas adsorption measurements were recorded on a Micromeritics 3Flex apparatus. The samples were degassed for 6 hours at 60 °C and 10^{-6} Torr prior to analysis. Surface area, pore size and volume values were calculated from N_2 adsorption-desorption isotherms (77 K) Specific surface area was calculated by multi-point Brunauer-Emmett-Teller (BET) method. Total pore volume values were taken at P/P_0=0.90.

Thermogravimetric analysis (TGA) was carried out with a TGA 550 (Waters/TA Instruments) apparatus between 25 and 600 ºC under ambient conditions (5ºC min^{-1} scan rate and an air flow of 90 mL·min^{-1}).

Flash chromatography for organic purification was performed in a Teledyne ISCO CombiFlash® EZ Prep integrated with a PurIon mass spectrometer using RediSep® Bronze silica columns and the indicated solvents.

^{1}H, ^{13}C and DEPT-135 (NMR) spectra were recorded on a Bruker AVIII 300 MHz and NEO 500 MHz instruments and were calibrated using residual undeuterated solvent ($CHCl_3$, DMSO, $(CD_3)_2CO$ at 7.26, 2.50 and 2.05 ppm for ^{1}H NMR, respectively, and 77.2, 39.5 and 29.8 ppm for ^{13}C NMR). The following abbreviations were used to explain multiplicities: s = singlet, d = doublet, t = triplet, q = quartet, p = pentuplet, dd = doublet of doublets, ddd = doublet of doublet of doublets, m = multiplet.

High resolution mass spectrometry (HRMS) performed with a high-resolution quadrupole time-of-flight mass spectrometer, QTOF (LC-MS/MS). The experiments were carried out with TripleTOF™ 5600 LC/MS/MS System, (AB SCIEX) apparatus. Ionization source: ESI/APCI. Temperature: 450 ºC, ion spray voltage (ISVF): 5500.

Circular dichroism (CD) spectra were recorded on a JASCO 1500 CD spectrometer using a 1 mm quartz cell cuvette with a scanning rate at 50 $nm \cdot min^{-1}$, a data pitch of 1 nm, a slit width of 1000 μm, a D.I.T. of 1 s and a continuous scanning mode. The baseline correction was performed with the spectrum of the solvent. All spectra were recorded for the wavelength range of 195-250 nm. Concentrations were selected to maintain a HV below 700 Volts. The data was smoothed using a Savitzky-Golay filter.

UV-Vis spectra were recorded on a JASCO V-750 spectrometer using a 10 mm quartz cell cuvette in the wavelength range of 200-600 nm.

Chiral HPLC separations were performed in an Agilent 1260 Infinity II LC System equipped with a CHIRALCEL® OD-H column (4.6 mm × 25 cm, 5 μm) and UV detection. Detection wavelengths were chosen to match the absorption maximum of the analyte.

S.2. SYNTHESIS AND CHARACTERIZATION OF THE STARTING MATERIAL

S.2.1. SYNTHESIS OF THE STARTING MATERIAL

The synthesis of the H_2TZDC linker was carried out following a procedure reported by our group.[2] The synthesis of UiO-68-TZDC was carried out following the procedure of the same work with minor modifications. Briefly, 18.0 mg of H_2TZDC (0.055 mmol) were suspended in a mixture of 3.5 mL of *N,N*-dimethylformamide and 40 µL of trifluoroacetic acid in a 5 mL teflon vial. Subsequently, 12.0 mg of anhydrous $ZrCl_4$ (0.051 mmol) were added. The vial was sealed and sonicated for 15 min. Finally, it was heated at 100 ºC for 72 h in an isothermal oven (heating rate 5 ºC · min^{-1}, cooling rate 0.1 ºC · min^{-1}).

After cooling down to room temperature, the deep pink crystals were washed with fresh *N,N*-dimethylformamide (4 x 15 mL). The material destined for characterization purposes was washed with fresh methanol (3 x 15 mL) and then interchanged and kept in fresh *n*-hexane. The material destined for functionalization was kept in *N,N*-dimethylformamide until its use, when it was washed with fresh methanol three times.

S.2.2. CHARACTERIZATION OF THE STARTING MATERIAL

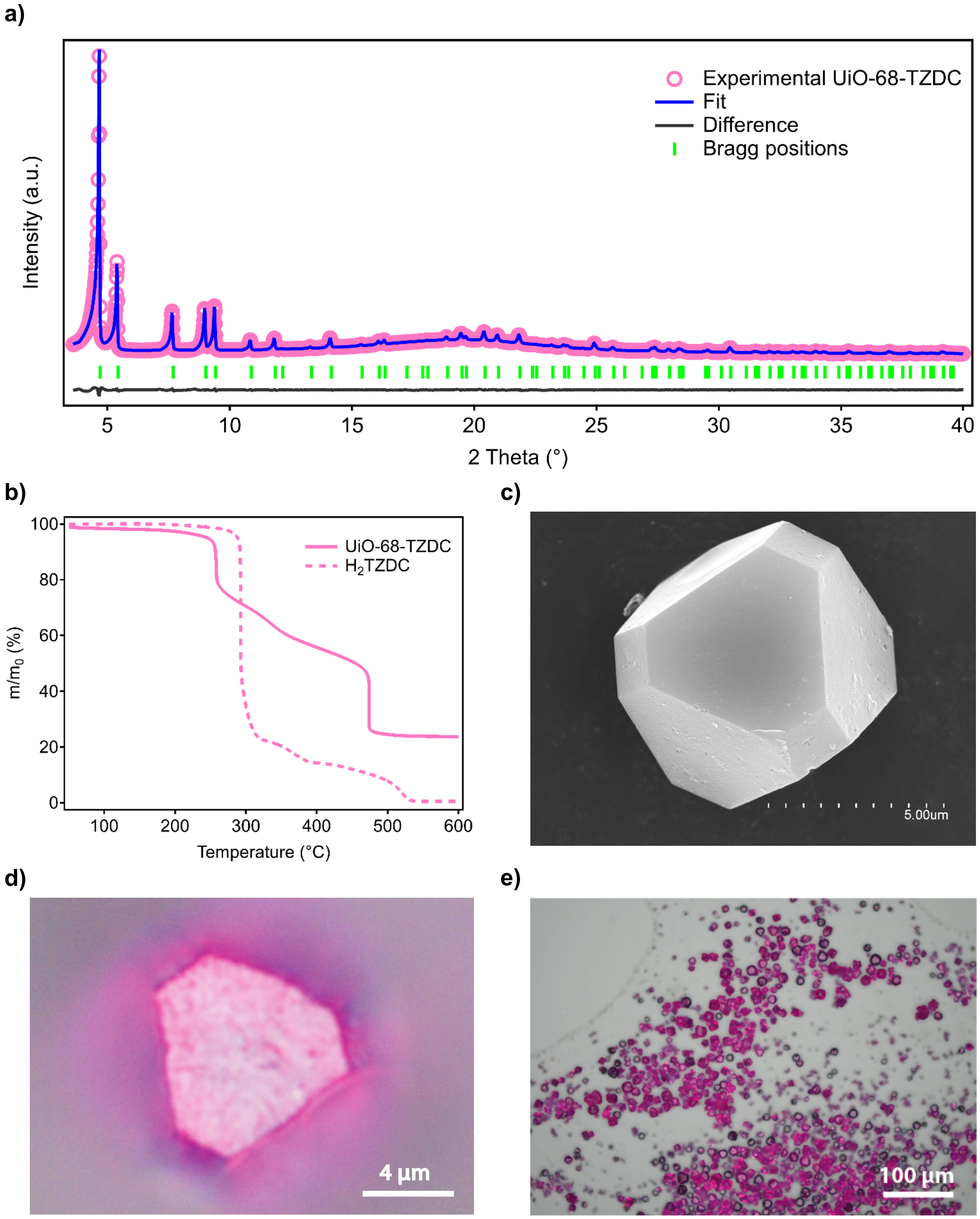

Figure S1. Characterization of UiO-68-TZDC via a) LeBail refinement. Fm$\bar{3}$m, a = b = c = 32.4905 Å, R_e = 1.02 %, R_p = 1.67 %, R_{wp} = 5.96 %, GoF = 2.27; b) thermogravimetric analysis; c) scanning electron microscopy; optical images with d) higher and e) lower magnification.

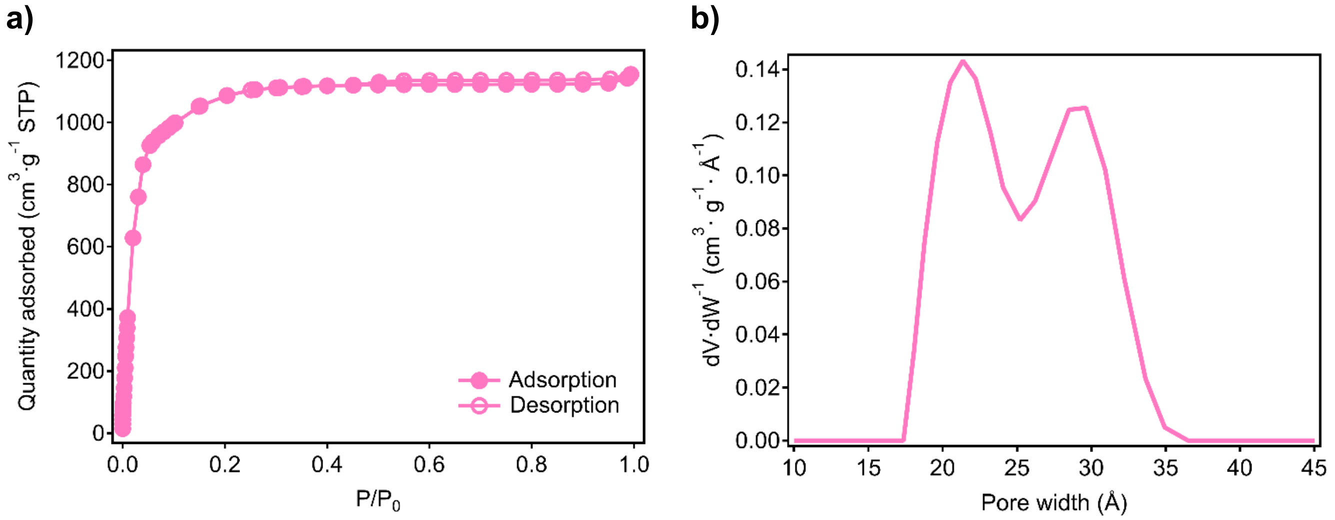

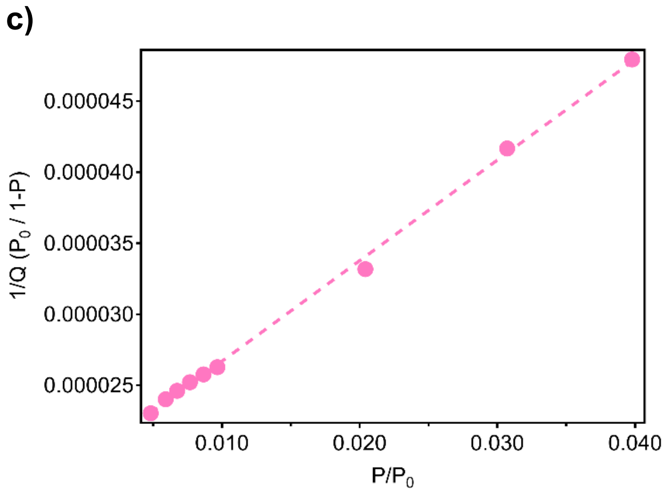

d)

**BET Area ($m^2 \cdot g^{-1}$)**	5982.7431 ± 90.8489
**Slope ($g \cdot cm^{-3}$ STP)**	0.000708 ± 0.000011
**Intercept ($g \cdot cm^{-3}$ STP)**	0.000020 ± 0.000000
**R^2**	0.9991490
**C**	37.085392
**Q_m ($cm^3 \cdot g^{-1}$ STP)**	1374.583
**$1/(\sqrt{C}+1)$**	0.141

Figure S2. Analysis of the N_2 adsorption/desorption isotherm of UiO-68-TZDC at 77 K. a) N_2 adsorption isotherm; b) Pore Size Distribution calculated by SWNT-NLDFT (regularization = 0.1); c) Multi-Point BET analysis; d) main parameters calculated from the multi-point BET analysis.

S.3. SYNTHESIS AND CHARACTERIZATION OF THE DIENOPHILES

S.3.1. SYNTHESIS OF THE DIENOPHILE PRECURSORS

Synthesis of methyl (4-acetylbenzoyl)-(*L*)-alaninate (1)

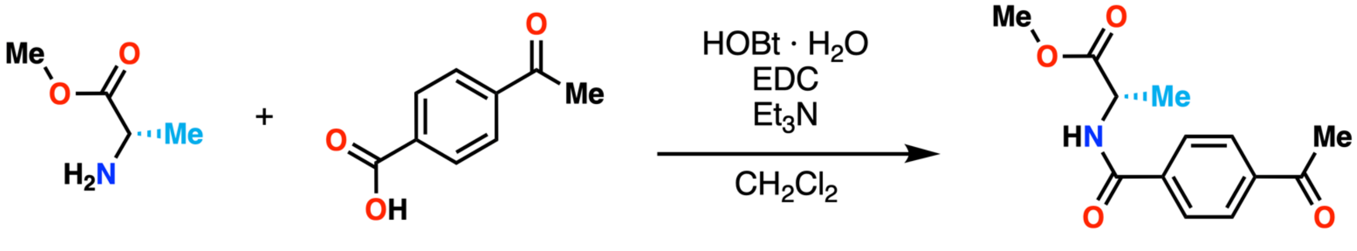

1

Scheme S1. Methyl (4-acetylbenzoyl)-(*L*)-alaninate (**1**) synthesis.

In a 100 mL round-bottomed flask equipped with a stirring bar, 4-acetylbenzoic acid (1.29 g, 7.9 mmol, 1.1 equiv.) was dissolved with 40 mL of anhydrous CH_2Cl_2 at room temperature under Ar atmosphere. Subsequently, 1-hydroxybenzotriazole hydrate (HOBt · H_2O, 1.32 g, 8.6 mmol, 1.2 equiv.), 1-ethyl-3-(3-dimethylaminopropyl)carbodiimide (EDC, 1.65 g, 8.6 mmol, 1.2 equiv.) and triethylamine (1.2 mL, 8.6 mmol, 1.2 equiv.) were added. Finally, (*L*)-alanine methyl ester hydrochloride (1.00 g, 7.2 mmol, 1 equiv.) was added and the mixture was stirred for an additional 4 hours. The reaction was monitored by TLC. After completion, the reaction was washed three times with a saturated ammonium chloride aqueous solution, one time with a saturated sodium bicarbonate aqueous solution and one time with brine. The organic phase was dried over sodium sulfate, filtered and concentrated under vacuum. The crude residue was purified by flash chromatography on silica gel using a 40 g column (gradient starting with a 7:3 and ending with a 2:8 hexane:ethyl acetate mixture) to obtain **1** as a white solid with a 78% yield.

^{1}H NMR (300 MHz, $CDCl_3$): δ_H 8.02 – 7.97 (m, 2H), 7.90 – 7.85 (m, 2H), 6.86 (d, *J* = 7.2 Hz, 1H), 4.80 (p, *J* = 7.2 Hz, 1H), 3.80 (s, 3H), 2.63 (s, 3H), 1.53 (d, *J* = 7.1 Hz, 3H).

^{13}C NMR (75 MHz, $CDCl_3$, DEPT-135): δ_C 197.5 (C), 173.7 (C), 166.0 (C), 139.5 (C), 137.9 (C), 128.7 (CH), 127.5 (CH), 52.8 (CH), 48.8 (CH_3), 27.0 (CH_3), 18.7 (CH_3).

HRMS: $[M+H]^+$ calculated for $C_{13}H_{15}NO_4$ $[M+H]^+$: 250.1079; found: 250.1066.

Synthesis of methyl (4-acetylbenzoyl)-(*L*)-phenylalaninate (2)

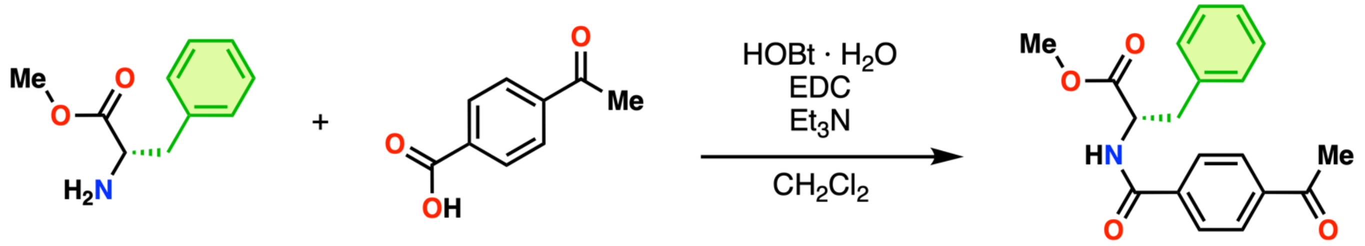

2

Scheme S2. Methyl (4-acetylbenzoyl)-(*L*)-phenylalaninate (**2**) synthesis.

In a 100 mL round-bottomed flask equipped with a stirring bar, 4-acetylbenzoic acid (0.84 g, 5.1 mmol, 1.1 equiv.) was dissolved with 45 mL of anhydrous CH_2Cl_2 at room temperature under Ar atmosphere. Subsequently, 1-hydroxybenzotriazole hydrate (HOBt · H_2O, 0.85 g, 5.6 mmol, 1.2 equiv.), 1-ethyl-3-(3-dimethylaminopropyl)carbodiimide (EDC, 1.07 g, 5.6 mmol, 1.2 equiv.) and triethylamine (0.8 mL, 5.6 mmol, 1.2 equiv.) were added. Finally, (*L*)-phenylalanine methyl ester hydrochloride (1.00 g, 4.6 mmol, 1 equiv.) was added and the mixture was stirred for an additional 4 hours. The reaction was monitored by TLC. After completion, the reaction was washed three times with a saturated ammonium chloride aqueous solution, one time with a saturated sodium bicarbonate aqueous solution and one time with brine. The organic phase was dried over sodium sulfate, filtered and concentrated under vacuum. The crude residue was purified by flash chromatography on silica gel using a 40 g column (gradient starting with a 6:4 and ending with a 2:8 hexane:ethyl acetate mixture) to obtain **2** as a white solid with a 92% yield.

^{1}H NMR (300 MHz, DMSO): δ_H 9.05 (d, *J* = 7.8 Hz, 1H), 8.08 – 7.97 (m, 2H), 7.95 – 7.84 (m, 2H), 7.33 – 7.23 (m, 4H), 7.22 – 7.11 (m, 1H), 4.69 (ddd, *J* = 10.0, 7.8, 5.4 Hz, 1H), 3.65 (s, 3H), 3.19 (dd, *J* = 13.7, 5.4 Hz, 1H), 3.10 (dd, *J* = 13.7, 10.0 Hz, 1H), 2.61 (s, 3H).

^{13}C NMR (75 MHz, DMSO, DEPT-135): δ_C 197.7 (C), 172.0 (C), 165.7 (C), 138.9 (C), 137.6 (C), 137.4 (C), 129.1 (CH), 128.3 (CH), 128.2 (CH), 127.7 (CH), 126.5 (CH), 54.3 (CH), 52.0 (CH_3), 36.2 (CH_2), 27.0 (CH_3).

HRMS: $[M+H]^+$ calculated for $C_{19}H_{19}NO_4 + H^+$: 326.1392; found: 326.1396.

Synthesis of methyl (4-acetylbenzoyl)-(*L*)-histidinate (3)

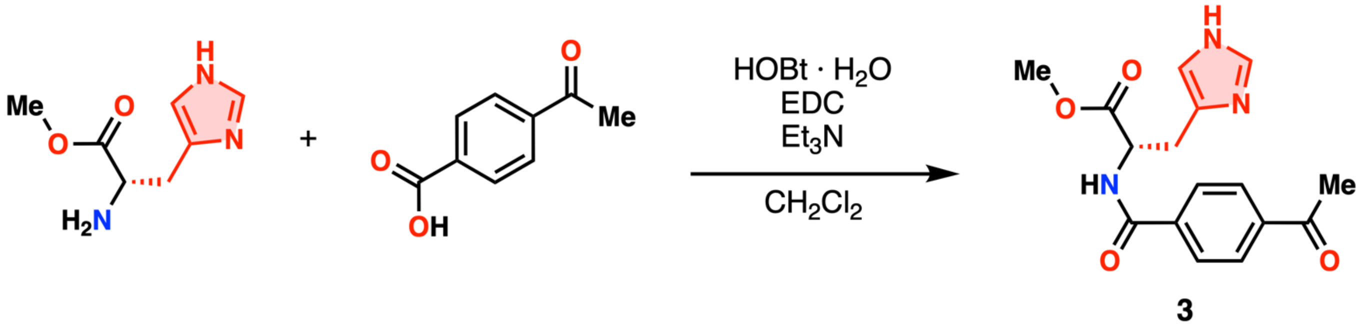

Scheme S3. Methyl (4-acetylbenzoyl)-(*L*)-histidinate (**3**) synthesis.

In a 100 mL round-bottomed flask equipped with a stirring bar, 4-acetylbenzoic acid (0.81 g, 5.0 mmol, 1.2 equiv.) was dissolved with 40 mL of anhydrous CH_2Cl_2 at room temperature under Ar atmosphere. Subsequently, 1-hydroxybenzotriazole hydrate ($HOBt \cdot H_2O$, 0.76 g, 5.0 mmol, 1.2 equiv.), 1-ethyl-3-(3-dimethylaminopropyl)carbodiimide (EDC, 0.95 g, 5.0 mmol, 1.2 equiv.) and triethylamine (0.7 mL, 5.0 mmol, 1.2 equiv.) were added. Finally, (*L*)-histidine methyl ester hydrochloride (1.00 g, 4.1 mmol, 1 equiv.) was added and the mixture was stirred for an additional 4 hours. The reaction was monitored by TLC. After completion, the reaction was washed three times with a saturated sodium bicarbonate aqueous solution and one time with brine. The organic phase was dried over sodium sulfate, filtered and concentrated under vacuum. The crude residue was purified by flash chromatography on silica gel using a 40 g column (gradient starting with pure CH_2Cl_2 and ending with a 9:1 CH_2Cl_2:MeOH mixture) to obtain **3** as a white solid with a 53% yield.

^{1}H NMR (500 MHz, DMSO): δ_H 9.07 (d, J = 7.4 Hz, 1H), 8.07 – 8.01 (m, 2H), 7.98 – 7.92 (m, 2H), 7.57 (d, J = 1.2 Hz, 2H), 6.87 (d, J = 1.2 Hz, 1H), 4.70 (dt, J = 7.5, 6.2 Hz, 1H), 3.63 (s, 3H), 3.07 (d, J = 6.1 Hz, 2H), 2.62 (s, 3H).

^{13}C NMR (126 MHz, DMSO, DEPT-135): δ_C 197.7 (C), 172.0 (C), 165.5 (C), 138.9 (C), 137.5 (C), 135.1 (CH), 128.2 (CH), 127.6 (CH), 53.2 (CH), 52.0 (CH_3), 28.6 (CH_2), 26.7 (CH_3).

HRMS: $[M+H]^+$ calculated for $C_{16}H_{17}N_3O_4 + H^+$: 316.1297; found: 316.1297.

S.3.2. SYNTHESIS OF THE DIENOPHILES

General considerations

The following syntheses correspond to the formation of compounds which are prone to hydrolyze when exposed to moisture or acidic media. Therefore, special care was taken in the process. Drying of dichloromethane and triethylamine in Schlenk tubes over molecular sieves led to improved yields of the reactions. The purity of tert-butyldimethylsilyl trifluoromethanesulfonate also affects the overcoming of the syntheses. Therefore, it was stored and added under inert conditions. Moreover, flash chromatography was performed with a small percentage of triethylamine in the mobile phase for deactivating the silica gel. Finally, the products were stored under inert conditions at -20 ºC until their use.

The synthetic protocol of the dienophiles was based in a work from Gademann and co-workers[3] with some modifications.

Synthesis of methyl (4-(1-((*tert*-butyldimethylsilyl)oxy)vinyl)benzoyl)-(*L*)-alaninate (4)

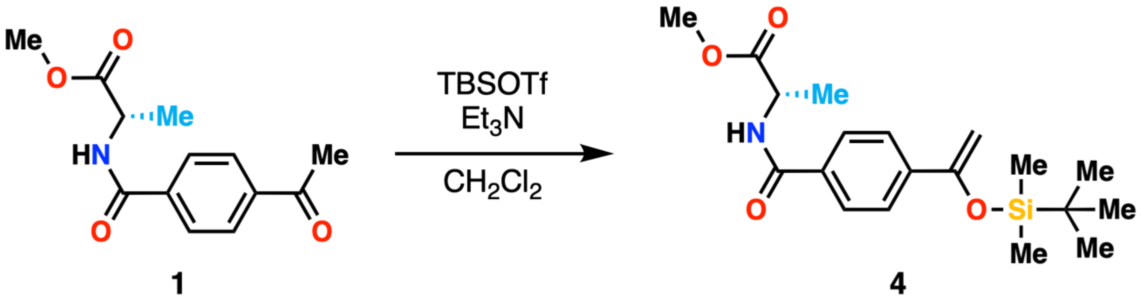

Scheme S4. Methyl (4-(1-((*tert*-butyldimethylsilyl)oxy)vinyl)benzoyl)-(*L*)-alaninate (**4**) synthesis.

Compound **1** (1.00 g, 4.0 mmol, 1 equiv.) was dissolved with 20 mL of anhydrous CH_2Cl_2 at room temperature under Ar atmosphere in a dried 50 mL round-bottomed flask equipped with a stirring bar. Triethylamine (1.1 mL, 8.0 mmol, 2 equiv.) was added to the mixture, followed by *tert*-butyldimethylsilyl trifluoromethanesulfonate (TBSOTf, 1.4 mL, 6.0 mmol, 1.5 equiv.) at 0 ºC. The reaction mixture was allowed to warm to room temperature and stirred for 30 min. The reaction was monitored by TLC and concentrated under vacuum after completion. The crude residue was purified by flash chromatography on silica gel (gradient starting with 1% triethylamine in hexane and ending in a 7:3 hexane (1% triethylamine):ethyl acetate mixture) to obtain **4** as a yellowish oil with an 85% yield.

^{1}H NMR (500 MHz, DMSO): δ_H 8.79 (d, *J* = 7.0 Hz, 1H), 7.92 – 7.82 (m, 2H), 7.74 – 7.64 (m, 2H), 5.16 (d, *J* = 2.1 Hz, 1H), 4.54 (d, *J* = 2.1 Hz, 1H), 4.47 (p, *J* = 7.2 Hz, 1H), 3.64 (s, 3H), 1.40 (d, *J* = 7.3 Hz, 3H), 0.97 (s, 9H), 0.21 (s, 6H).

^{13}C NMR (126 MHz, DMSO, DEPT-135): δ_C 173.1 (C), 165.8 (C), 154.2 (C), 139.8 (C), 133.3 (C), 127.5 (CH), 124.6 (CH), 93.1 (CH_2), 51.9 (CH), 48.3 (CH_3), 25.6 (CH_3), 18.0 (C), 16.7 (CH_3), -4.7 (CH_3).

HRMS: $[M+H]^+$ calculated for $C_{19}H_{29}NO_4Si + H^+$: 364.1944; found: 364.1939.

Synthesis of methyl (4-(1-((*tert*-butyldimethylsilyl)oxy)vinyl)benzoyl)-(*L*)-phenylalaninate (5)

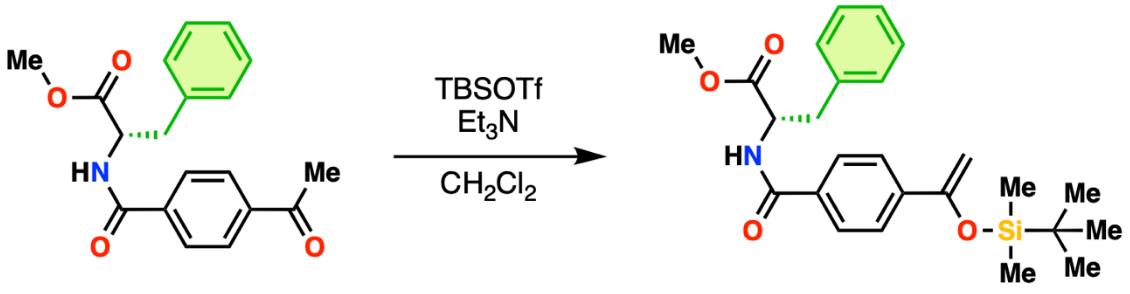

2 **5**

Scheme S5. Methyl (4-(1-((*tert*-butyldimethylsilyl)oxy)vinyl)benzoyl)-(*L*)-phenylalaninate (**5**) synthesis.

Compound **2** (1.00 g, 3.1 mmol, 1 equiv.) was dissolved with 20 mL of anhydrous CH_2Cl_2 at room temperature under Ar atmosphere in a dried 50 mL round-bottomed flask equipped with a stirring bar. Triethylamine (0.9 mL, 6.2 mmol, 2 equiv.) was added to the mixture, followed by *tert*-butyldimethylsilyl trifluoromethanesulfonate (TBSOTf, 1.1 mL, 4.6 mmol, 1.5 equiv.) at 0 ºC. The reaction mixture was allowed to warm to room temperature and stirred for 30 min. The reaction was monitored by TLC and concentrated under vacuum after completion. The crude residue was purified by flash chromatography on silica gel (gradient starting with 1% triethylamine in hexane and ending in a 7:3 hexane (1% triethylamine):ethyl acetate mixture) to obtain **5** as a yellowish oil with a 78% yield.

^{1}H NMR (500 MHz, DMSO): δ_H 8.86 (d, *J* = 7.8 Hz, 1H), 7.83 – 7.78 (m, 2H), 7.70 – 7.65 (m, 2H), 7.33 – 7.24 (m, 4H), 7.22 – 7.15 (m, 1H), 5.14 (d, *J* = 2.1 Hz, 1H), 4.67 (ddd, *J* = 10.1, 7.8, 5.2 Hz, 1H), 4.53 (d, *J* = 2.1 Hz, 1H), 3.64 (s, 3H), 3.17 (dd, J = 13.7, 5.2 Hz, 1H), 3.10 (dd, J = 13.8, 10.1 Hz, 1H), 0.97 (s, 9H), 0.20 (s, 6H).

^{13}C NMR (126 MHz, DMSO, DEPT-135): δ_C 172.2 (C), 166.0 (C), 154.1 (C), 139.9 (C), 137.7 (C), 133.3 (C), 129.0 (CH), 128.2 (CH), 127.5 (CH), 126.5 (CH), 124.7 (CH), 93.1 (CH_2), 54.3 (CH), 51.9 (CH_3), 36.2 (CH_2), 25.7 (CH_3), 18.0 (C), -4.8 (CH_3)

HRMS: $[M+H]^+$ calculated for $C_{25}H_{33}NO_4Si + H^+$: 440.2257; found: 440.2247.

Synthesis of methyl (4-(1-((*tert*-butyldimethylsilyl)oxy)vinyl)benzoyl)-(*L*)-histidinate (6)

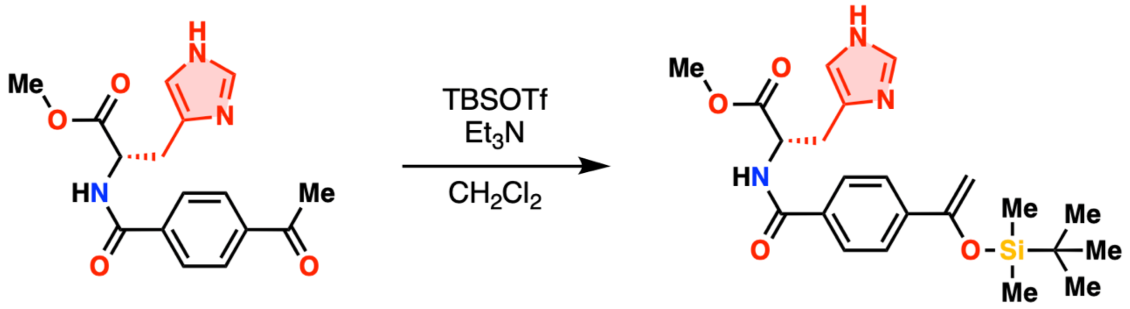

Scheme S6. Methyl (4-(1-((*tert*-butyldimethylsilyl)oxy)vinyl)benzoyl)-(*L*)-histidinate (**6**) synthesis.

Compound **3** (1.00 g, 4.0 mmol, 1 equiv.) was dissolved with 20 mL of anhydrous CH_2Cl_2 at room temperature under Ar atmosphere in a dried 50 mL round-bottomed flask equipped with a stirring bar. Triethylamine (1.1 mL, 8.0 mmol, 2 equiv.) was added to the mixture, followed by *tert*-butyldimethylsilyl trifluoromethanesulfonate (TBSOTf, 1.4 mL, 6.0 mmol, 1.5 equiv.) at 0 ºC. The reaction mixture was allowed to warm to room temperature and stirred for 30 min. The reaction was monitored by TLC and concentrated under vacuum after completion. The crude residue was purified by flash chromatography on silica gel (gradient starting with 1% triethylamine in CH_2Cl_2 and ending in a 95:5 CH_2Cl_2 (1% triethylamine):MeOH mixture) to obtain **6** as a yellowish oil with a 57% yield.

^{1}H NMR (500 MHz, $(CD_3)_2CO$): δ_H 8.83 (d, J = 7.2 Hz, 1H), 7.97 – 7.86 (m, 2H), 7.81 – 7.71 (m, 2H), 7.66 (d, J = 1.3 Hz, 1H), 6.99 (d, J = 1.2 Hz, 1H), 5.11 (d, J = 2.0 Hz, 1H), 4.83 (ddd, J = 7.3, 6.1, 4.7 Hz, 1H), 4.56 (d, J = 2.0 Hz, 1H), 3.63 (s, 3H), 3.19 (dd, J = 14.6, 5.8 Hz, 1H), 3.11 (dd, J = 14.9, 4.4 Hz, 1H), 1.02 (s, 9H), 0.25 (s, 6H).

^{13}C NMR (126 MHz, $(CD_3)_2CO$, DEPT-135): δ_C 172.6 (C), 166.5 (C), 156.0 (C), 141.4 (C), 136.0 (CH), 135.0 (C), 128.0 (CH), 126.0 (CH), 93.1 (CH_2), 54.1 (CH), 52.2 (CH_3), 47.0 (CH_2), 26.2 (CH_3), 18.9 (C), -4.5 (CH_3).

HRMS: $[M+H]^+$ calculated for $C_{22}H_{31}N_3O_4Si + H^+$: 430.2162; found: 430.2159.

S.4. CLICK FUNCTIONALIZATION OF THE MATERIALS

The inclusion of the chiral residues into the framework was based on a procedure reported by our group[4] with some modifications. Briefly, UiO-68-TZDC (20 mg, 0.015 mmol, 1 equiv.) was suspended in 5 mL of anhydrous methanol under Ar atmosphere in a 20 mL Pyrex culture tube capped with a septum. The amino acid dienophile (0.308 mmol, 20 equiv.) was dissolved in 5 mL of anhydrous methanol under Ar atmosphere and subsequently added with a syringe to the Pyrex culture tube containing the material.

The resulting mixture was stirred in an orbital shaker at 60 ºC for 48 hours. The progress of the click reaction can be easily tested macroscopically, as the linkers progressively lose their characteristic color, which can be observed with the naked eye. After this time, the solids were thoroughly washed with fresh methanol and kept in fresh *n*-hexane.

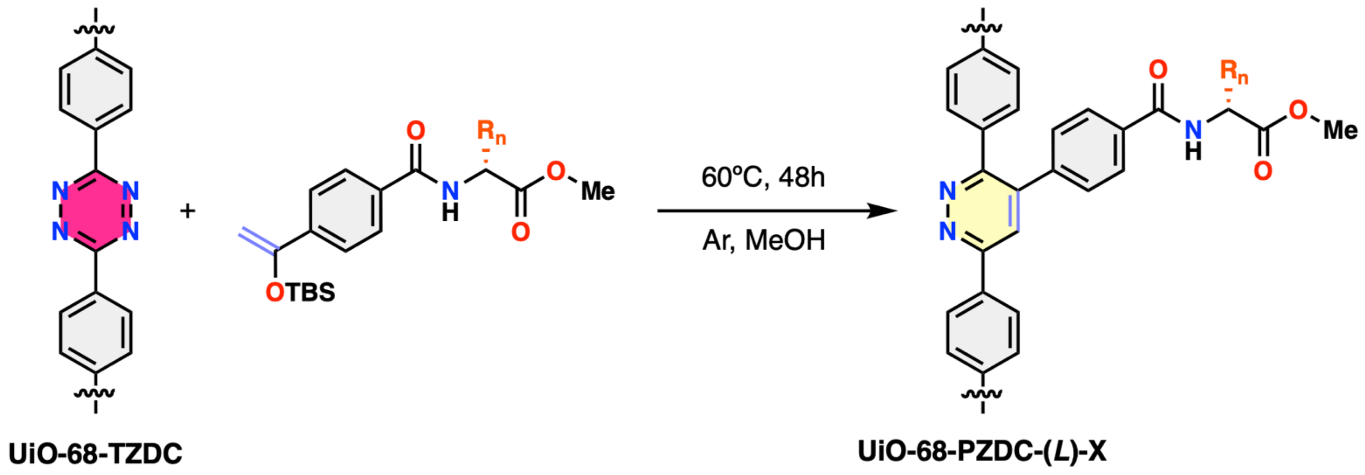

Scheme S7. Inverse electron-demand Diels–Alder (iEDDA) reaction.

S.5. CHARACTERIZATION OF THE MODIFIED MATERIALS

S.5.1. LE BAIL REFINEMENTS

PXRD patterns were collected using capillaries filled with *n*-hexane and polycrystalline wet samples at room temperature. To test the respectfulness of the modification method with the crystallinity of the material, Le Bail refinements were performed using UiO-68-TZDC data (CCDC 2023345) as starting parameters.

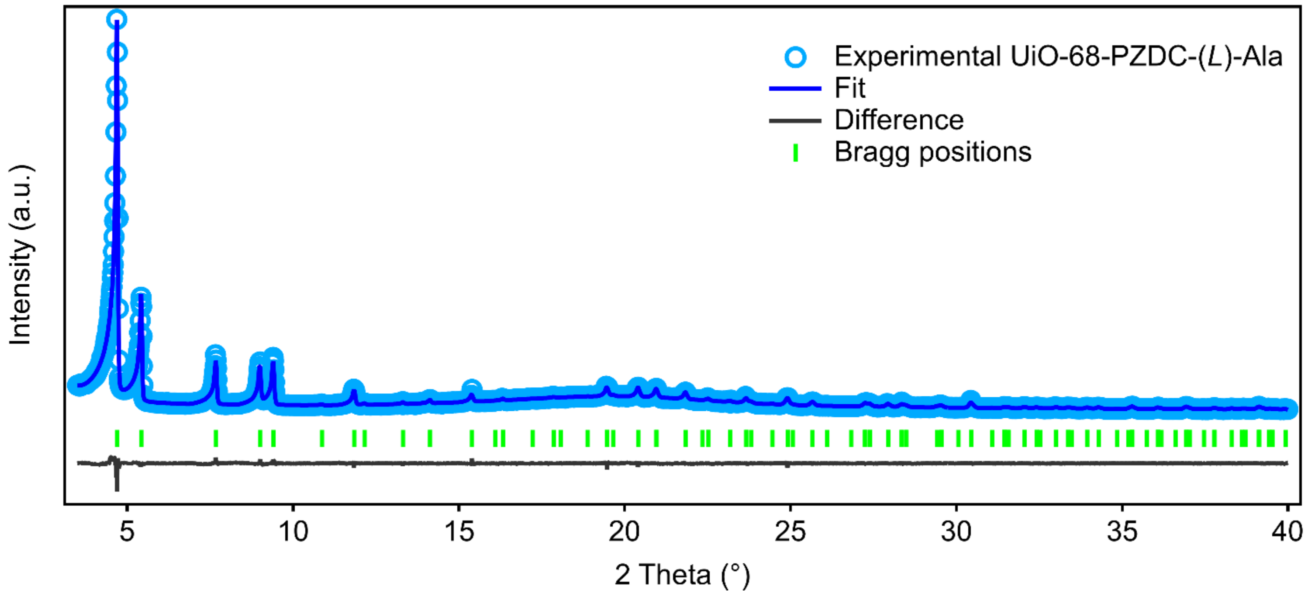

Figure S3. Le Bail refinement of UiO-68-PZDC-(*L*)-Ala (λ = 1.5406 Å).

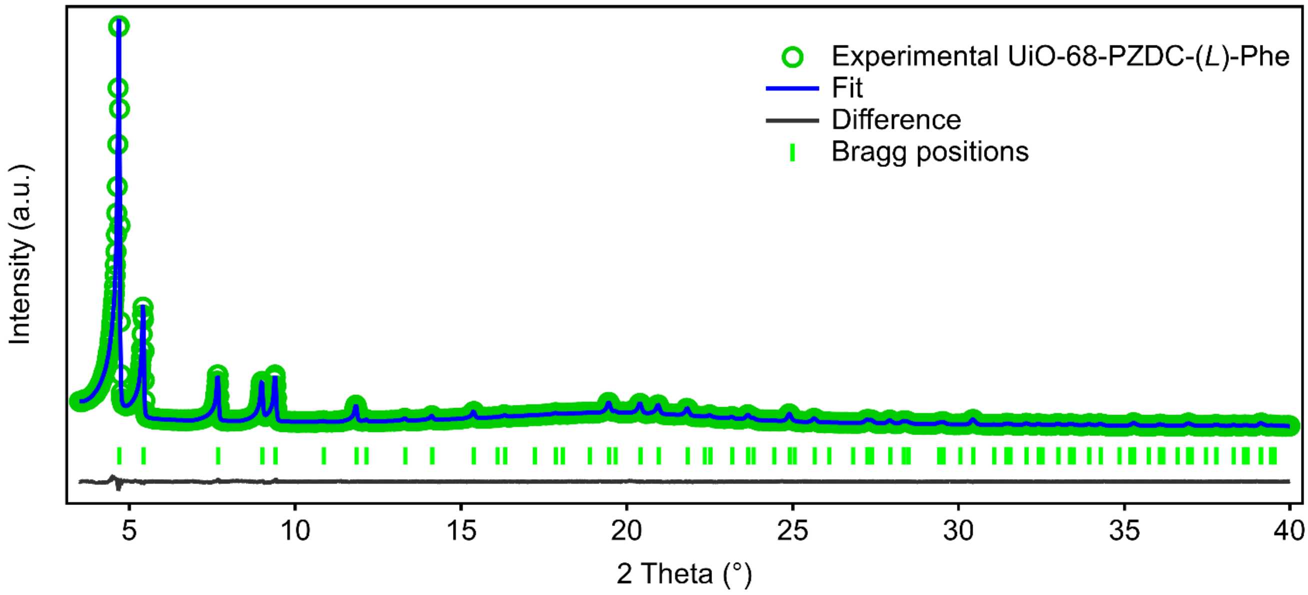

Figure S4. Le Bail refinement of UiO-68-PZDC-(*L*)-Phe (λ = 1.5406 Å).

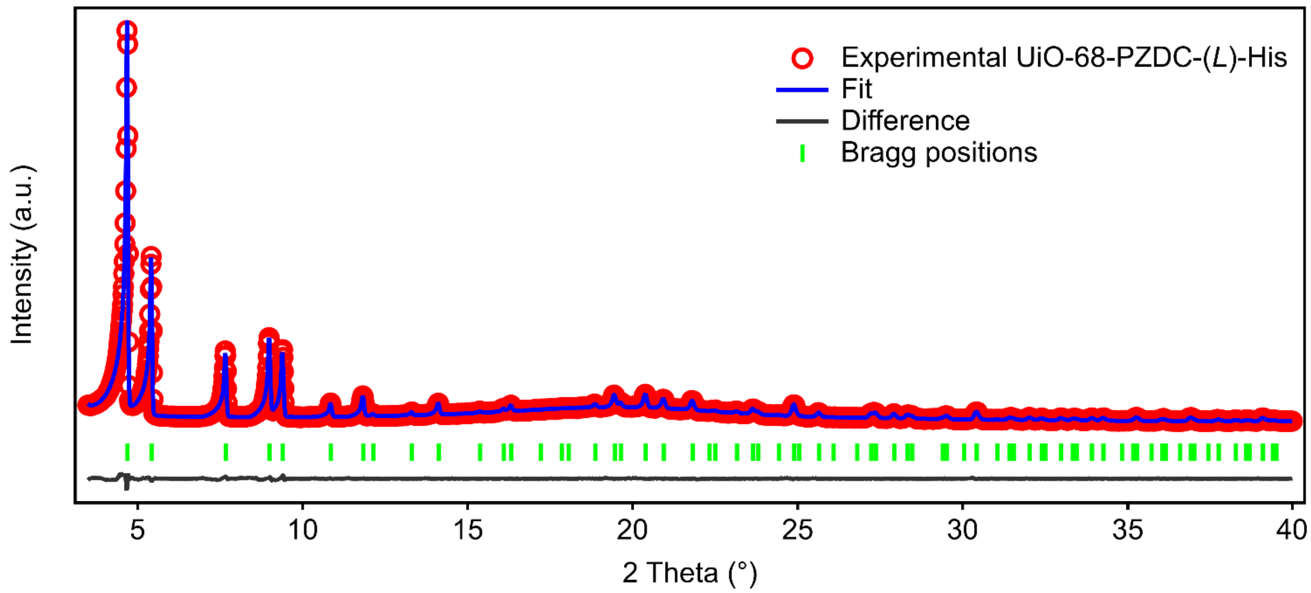

Figure S5. Le Bail refinement of UiO-68-PZDC-(*L*)-His (λ = 1.5406 Å).

Table S1. Summary of the parameters obtained from LeBail refinements.

	a = b = c (Å)	V ($Å^3$)	R_e (%)	R_p (%)	R_{wp} (%)	gof
**(*L*)-Ala**	32.5363	34444	1.57	2.27	7.30	2.10
**(*L*)-Phe**	32.5402	34456	1.55	1.77	5.12	1.48
**(*L*)-His**	32.5579	34511	1.56	2.09	5.60	1.72

S.5.2. OPTICAL IMAGES

After the click reactions, the materials were observed with an optical microscope, which reveals the loss of tetrazines' characteristic color. Moreover, the perfect retention of crystal morphology (truncated octahedra) is also checked.

a) **b)**

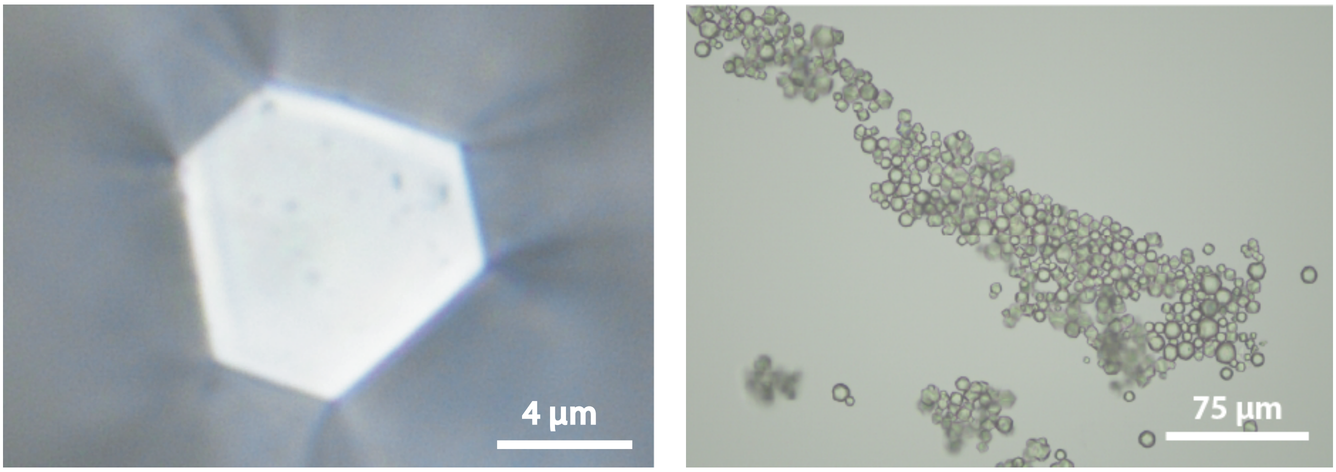

Figure S6. Optical images of UiO-68-PZDC-(*L*)-Ala with a) higher and b) lower magnification objective.

a) **b)**

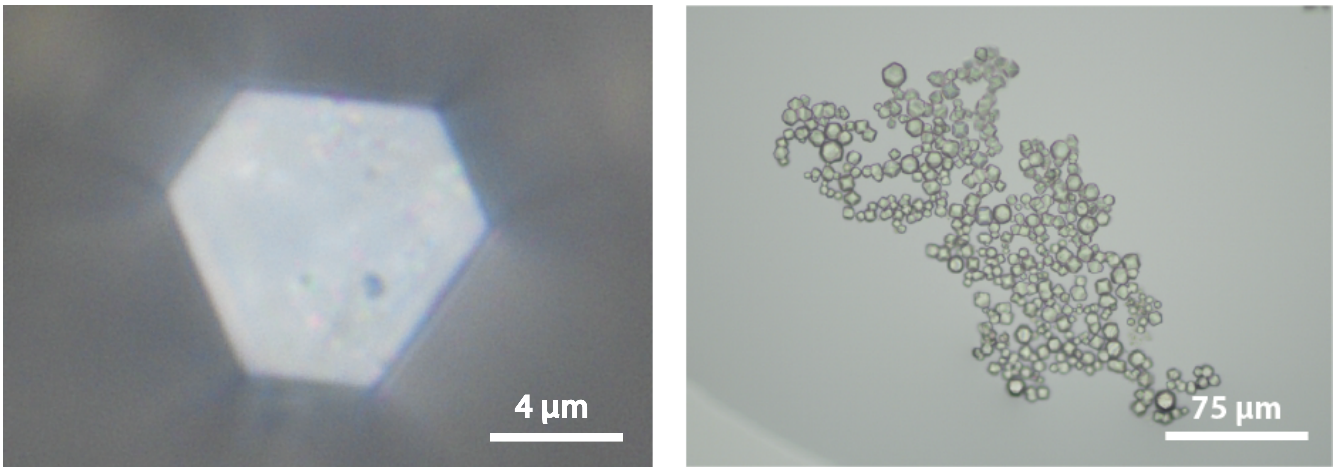

Figure S7. Optical images of UiO-68-PZDC-(*L*)-Phe with a) higher and b) lower magnification objective.

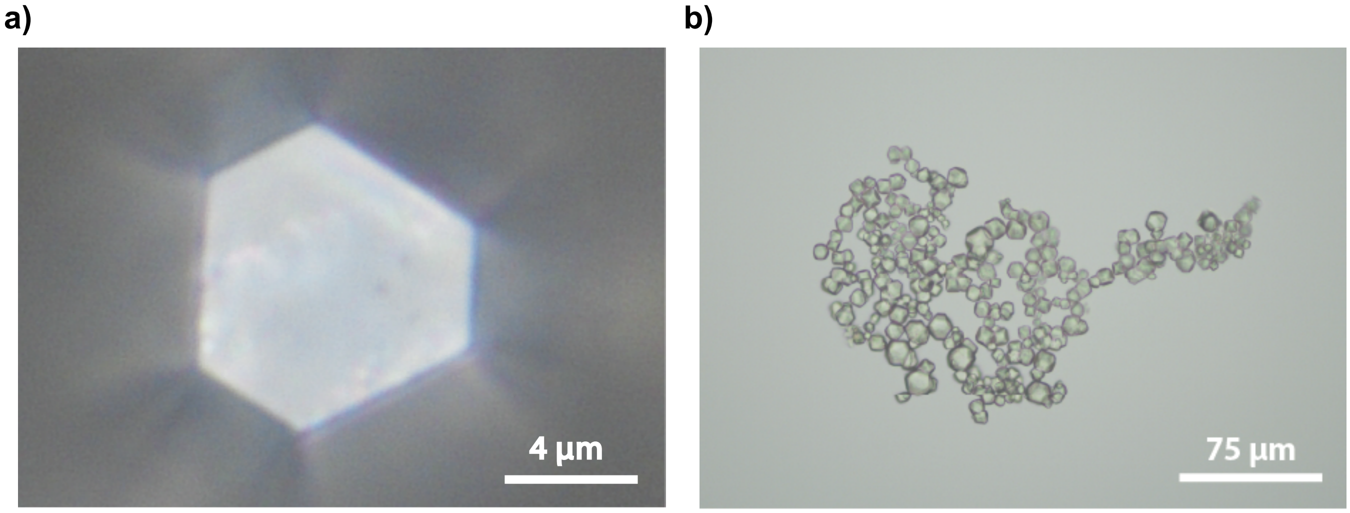

Figure S8. Optical images of UiO-68-PZDC-(*L*)-His with a) higher and b) lower magnification objective.

S.5.3. SCANNING ELECTRON MICROSCOPY

Images taken from a scanning electron microscope further tested the minimum effect from the functionalization method on the crystals' morphology.

a) **b)**

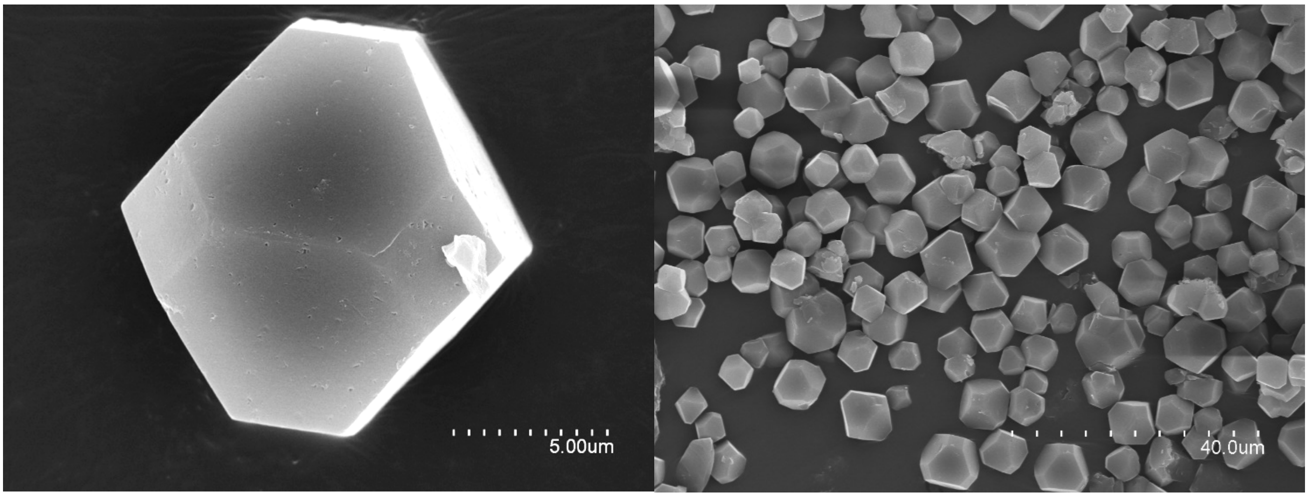

Figure S9. Scanning electron microscopy images of UiO-68-PZDC-(*L*)-Ala with a) higher and b) lower magnification.

a) **b)**

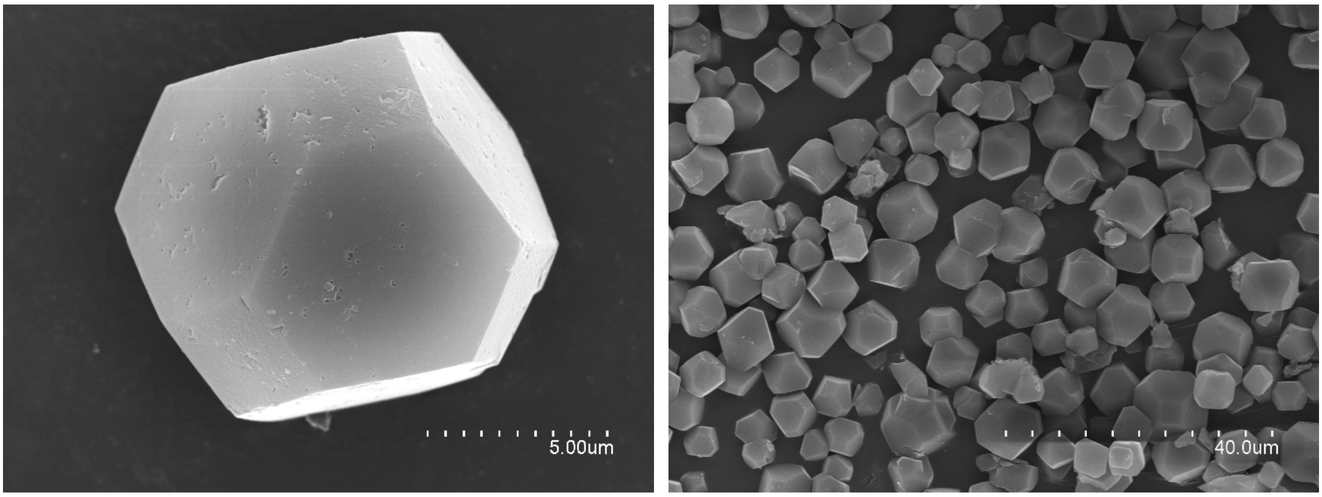

Figure S10. Scanning electron microscopy images of UiO-68-PZDC-(*L*)-Phe with a) higher and b) lower magnification.

a) b)

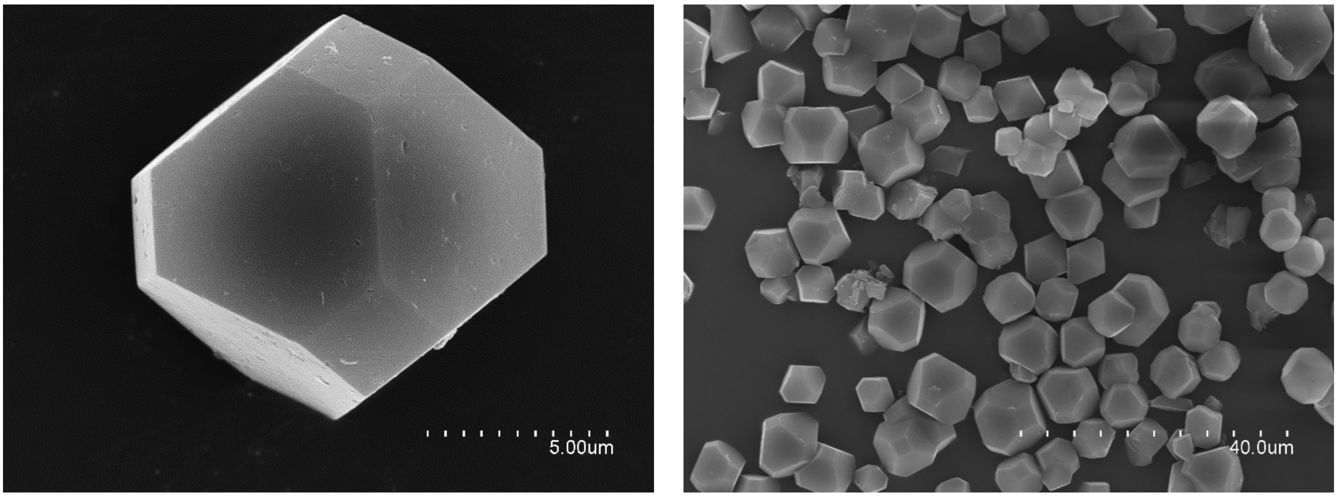

Figure S11. Scanning electron microscopy images of UiO-68-PZDC-(*L*)-His with a) higher and b) lower magnification.

S.5.4. ANALYSIS OF N_2 ISOTHERMS AT 77 K

Gas adsorption measurements were performed ex-situ on solids exchanged in hexane. Surface area, pore size and volume values were calculated from nitrogen adsorption-desorption isotherms (77 K) recorded on a *Micromeritics 3Flex* apparatus. Samples were degassed overnight at 60ºC and 10^{-6} Torr prior to analysis. Brunauer-Emmett-Teller (BET) Surface area analysis were performed as recommended for microporous and mesoporous materials.[5] Specific surface area (SA) was calculated by multi-point Brunauer-Emmett-Teller (BET) method. Total pore volume was taken at P/P_0=0.9. Pore size distribution was analysed by using the solid density functional theory (SWDFT) for the adsorption branch by assuming a cylindrical pore model (N_2@77-Carb Cyl Pores, SWNT, NLDFT in MicroActive software).

a) b)

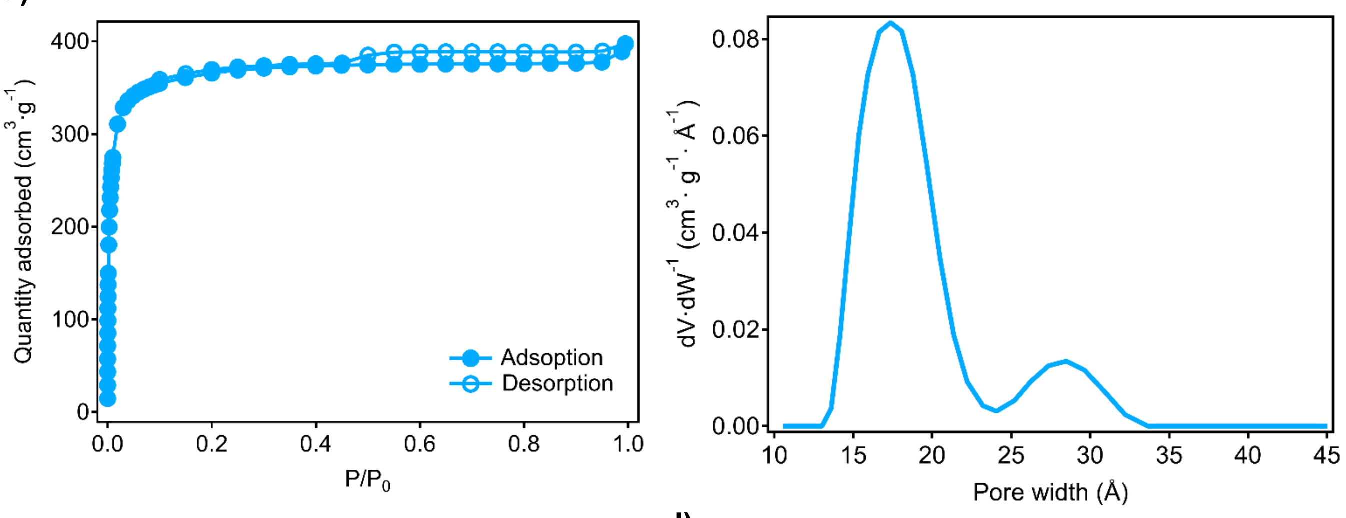

c)

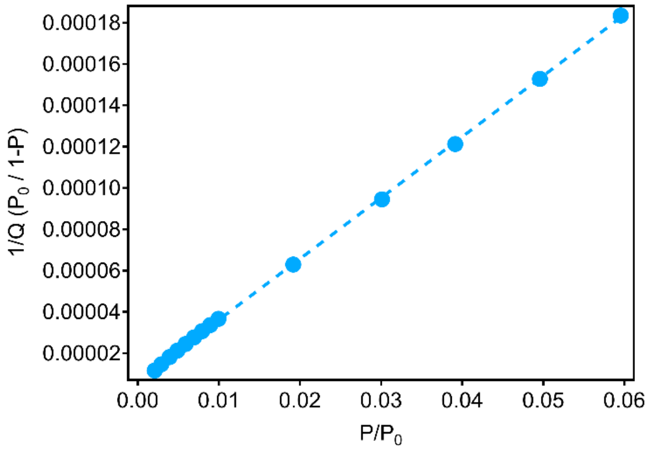

d)

**BET Area ($m^2 \cdot g^{-1}$)**	1470.5932 ± 5.3508
**Slope ($g \cdot cm^{-3}$ STP)**	0.002953 ± 0.000011
**Intercept ($g \cdot cm^{-3}$ STP)**	0.000007 ± 0.000000
**R^2**	0.9999203
**C**	449.408714
**Q_m ($cm^3 \cdot g^{-1}$ STP)**	337.8672
**$1/(\sqrt{C}+1)$**	0.045

Figure S12. Analysis of the N_2 adsorption/desorption isotherm of UiO-68-PZDC-(*L*)-Ala at 77 K. a) N_2 adsorption isotherm; b) Pore Size Distribution calculated by SWNT-NLDFT (regularization = 0.1); c) Multi-Point BET analysis; d) main parameters calculated from the multi-point BET analysis

a) b)

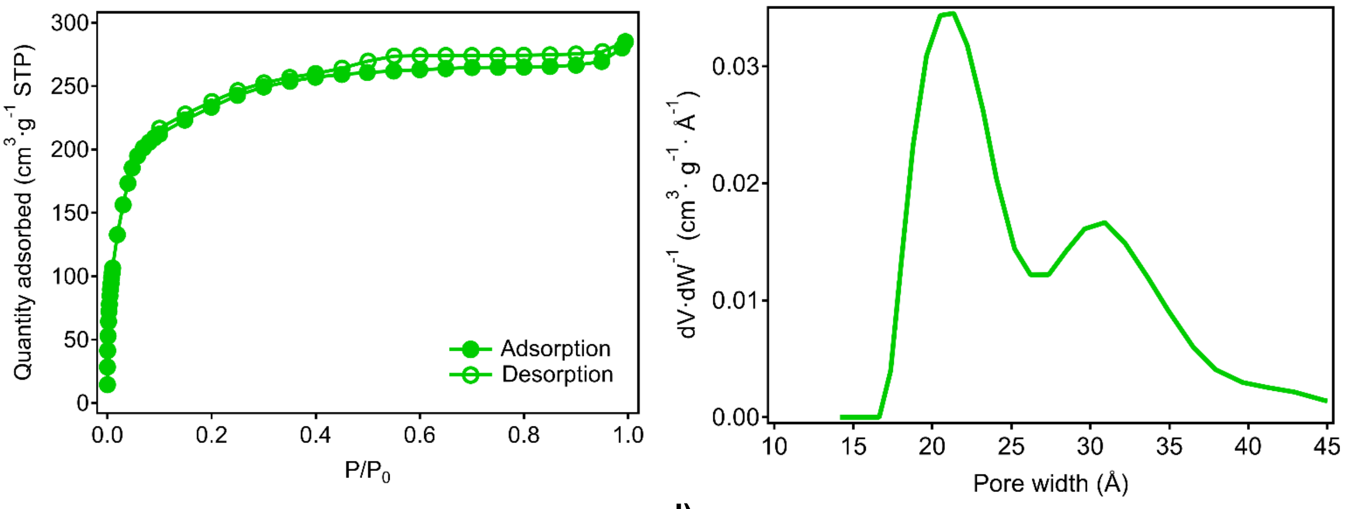

c)

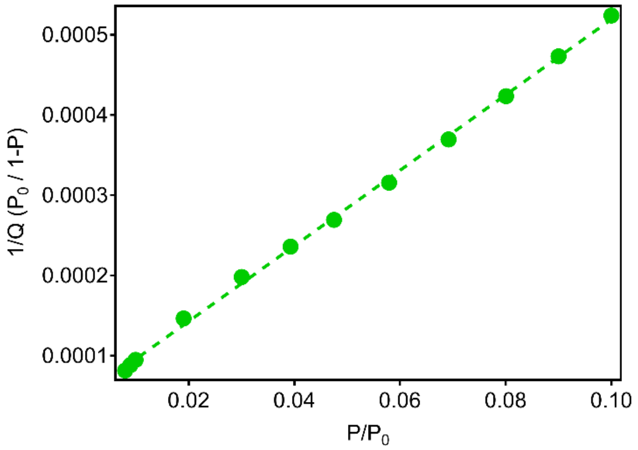

d)

**BET Area ($m^2 \cdot g^{-1}$)**	916.2768 ± 9.0336
**Slope ($g \cdot cm^{-3}$ STP)**	0.004701 ± 0.000047
**Intercept ($g \cdot cm^{-3}$ STP)**	0.000049 ± 0.000003
**R^2**	0.9995058
**C**	97.317307
**Q_m ($cm^3 \cdot g^{-1}$ STP)**	210.514
**$1/(\sqrt{C}+1)$**	0.092

Figure S13. Analysis of the N_2 adsorption/desorption isotherm of UiO-68-PZDC-(*L*)-Phe at 77 K. a) N_2 adsorption isotherm; b) Pore Size Distribution calculated by SWNT-NLDFT (regularization = 0.1); c) Multi-Point BET analysis; d) main parameters calculated from the multi-point BET analysis

a)

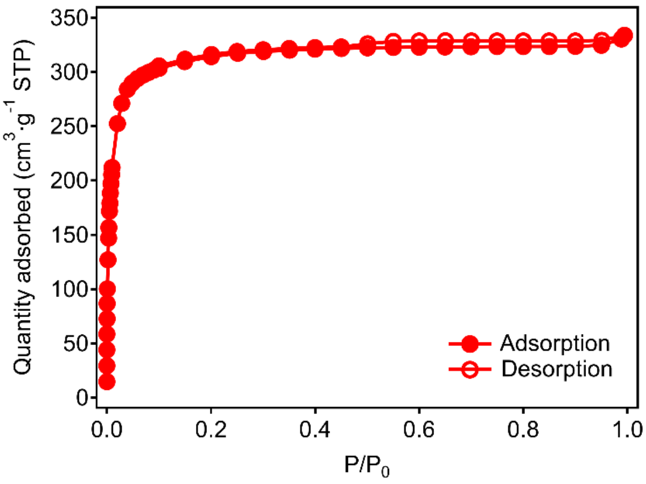

b)

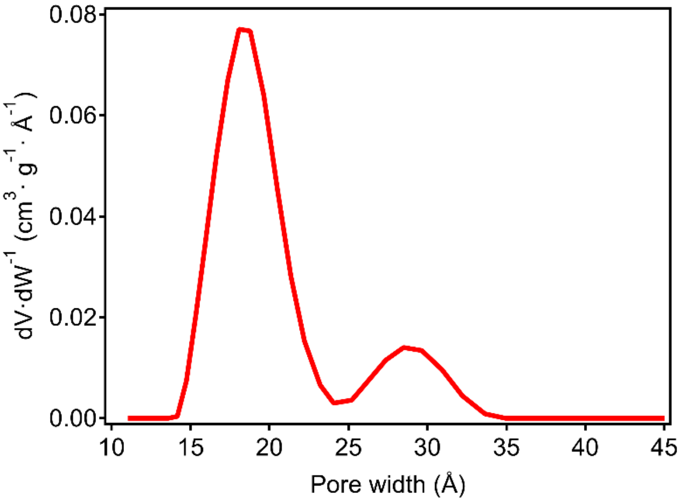

c)

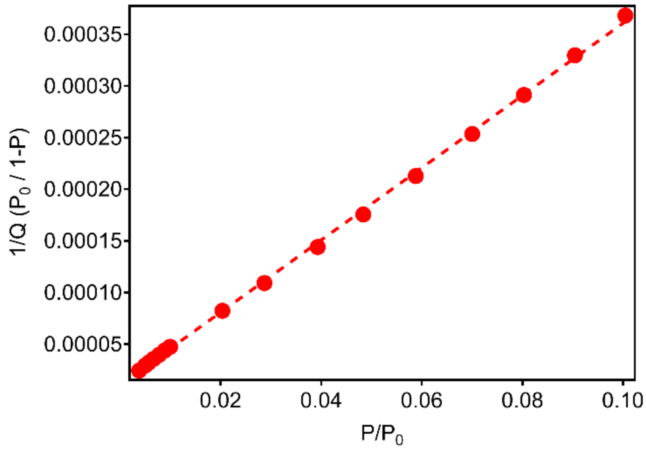

d)

**BET Area ($m^2 \cdot g^{-1}$)**	1240.6772 ± 7.8485
**Slope ($g \cdot cm^{-3}$ STP)**	0.003497 ± 0.000022
**Intercept ($g \cdot cm^{-3}$ STP)**	0.000011 ± 0.000001
**R^2**	0.9997189
**C**	323.493613
**Q_m ($cm^3 \cdot g^{-1}$ STP)**	285.0442
**$1/(\sqrt{C}+1)$**	0.053

Figure S14. Analysis of the N_2 adsorption/desorption isotherm of UiO-68-PZDC-(*L*)-His at 77 K. a) N_2 adsorption isotherm; b) Pore Size Distribution calculated by SWNT-NLDFT (regularization = 0.1); c) Multi-Point BET analysis; d) main parameters calculated from the multi-point BET analysis

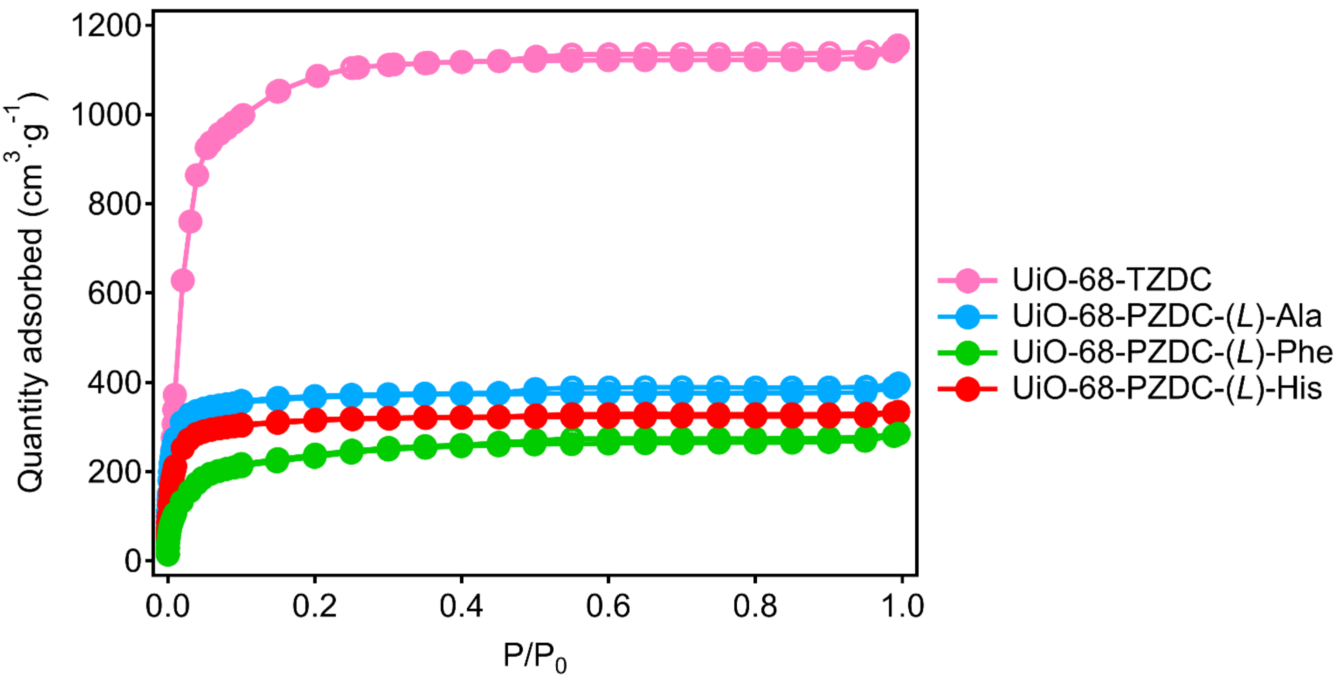

Figure S15. Comparison of N_2 isotherms at 77 K of the materials.

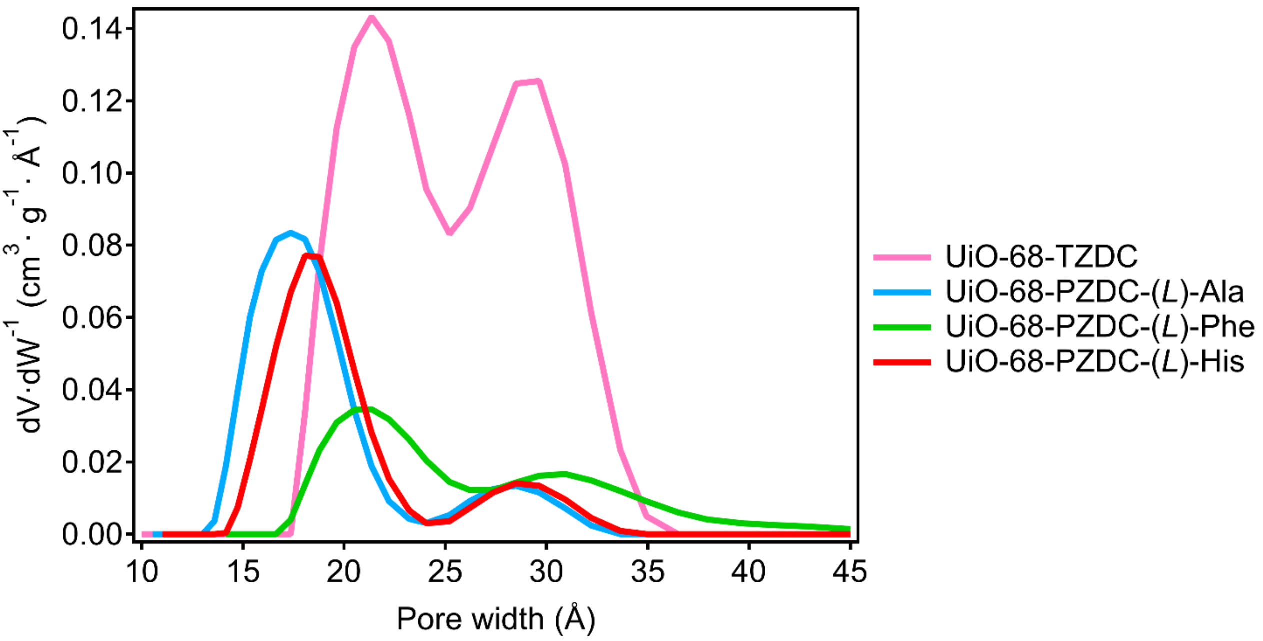

Figure S16. Comparison of Pore Size Distribution of the materials.

Table S2. Summary of the experimental adsorption data of the materials.

Framework	SA_{BET}[a] [$m^2 \cdot g^{-1}$]	V_t[b] [$cm^3 \cdot g^{-1}$]	PSD_{NLDFT}[c] [Å]	
			Tetrahedral	Octahedral
UiO-68-TZDC	5982.7	1.74	21	29
UiO-68-PZDC-(*L*)-Ala	1470.6	0.58	17	28
UiO-68-PZDC-(*L*)-Phe	916.3	0.41	21	29
UiO-68-PZDC-(*L*)-His	1240.7	0.50	18	29

[a] Specific surface area (Sa) was calculated by multi-point Brunauer-Emmett-Teller (BET) method. [b] Total pore volume at P/P_0=0.9. [c] Pore size distribution was analyzed by using the solid density functional theory (SWNT-NLDFT Carb Cyl Mesopore; regularization = 1.000) for the adsorption branch assuming a cylindrical pore model for the tetrahedral and octahedral pores of the materials.

S.5.5. ^{1}H NMR ANALYSIS

Before ^{1}H NMR analysis, the samples were dried from *n*-hexane and activated under vacuum at 120 ºC overnight. Subsequently, close to 1.5 mg of activated material were weighed in a precision analytical balance and suspended in 0.6 mL of a fumaric acid solution (0.02 M) in DMSO-d_6 and two drops of D_2SO_4-d_2 were added. Then, the mixture was digested by stirring for 5 minutes at 80 ºC. Finally, the resulting clear solution was transferred to an NMR tube, and the spectra was recorded.

In the case of UiO-68-TZDC, the partial solubility of the H_2TZDC in the media made it necessary to filtrate the solution before recording the spectra. However, in the case of the modified materials, there was no trouble at all solubilizing the digested samples, confirming the quantitative functionalization of these frameworks.

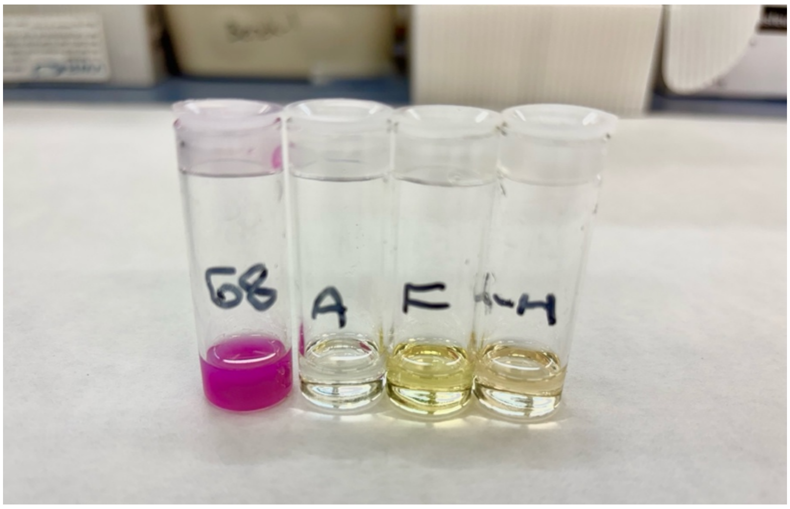

Figure S17. Resulting solutions after digesting samples of (from left to right) UiO-68-TZDC, UiO-68-PZDC-(*L*)-Ala, UiO-68-PZDC-(*L*)-Phe, UiO-68-PZDC-(*L*)-His.

Negligible characteristic peaks from unmodified material in the spectra made this even clearer. However, the poor solubility of H_2TZDC in the media made it impossible to obtain a reliable conversion value. That is why we chose to use fumaric acid as an internal standard for quantitative ^{1}H NMR calculations, assuming total solubility from the standard and the modified ligands.

For the calculations, we firstly analyzed the signals inherent to the modified ligands. Then, we integrated the signal at δ_H 6.6 corresponding to 2H from fumaric acid. By comparing the number of standard molecules per modified ligand, we could obtain its concentration in the media. Lastly, we calculated the yield of functionalization (x %) parting from a molecular formula of $C(L_{1-x}^{TZ}\ L_x^{AA})_6$ being C the cluster, L^{TZ} the unmodified linker and L^{AA} the modified one.

The relevant data for its obtention and the percentages of conversion for each material can be found in the following page. The high values of conversion assure the quantitativeness of the modification. The NMR spectra can be found in **Section S.8.**

Table S3. Summary of relevant data for the quantitative ^{1}H NMR calculations.

	Weighed MOF mass (mg)	$n_{fumaric}/n_{ligand}$	n_{ligand} (mmol)	Functionalization (%)
**(*L*)-Ala**	1.6	5.1	$2.35 \cdot 10^{-3}$	94 %
**(*L*)-Phe**	1.7	5.5	$2.18 \cdot 10^{-3}$	91 %
**(*L*)-His**	1.8	5.2	$2.31 \cdot 10^{-3}$	90 %

S.5.6. HRMS ANALYSIS

The sample treatment for digestion was the same as the used for ^{1}H NMR analysis excluding the inclusion of fumaric acid. Once the materials were digested, a drop was taken and dispersed in 0.5 mL of methanol. The positive mode was studied.

The HRMS results confirm the formation of the modified ligands, as shown in the table below.

Table S4. Summary of relevant data for the quantitative ^{1}H NMR calculations.

	Molecule	Chemical formula	Theoretical $[M+H]^+$	Experimental $[M+H]^+$
**(*L*)-Ala**	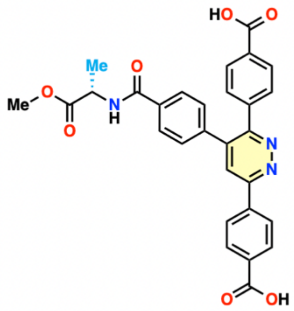	$C_{29}H_{23}N_3O_7$	526.1614	526.1598
**(*L*)-Phe**	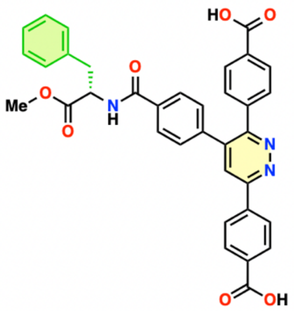	$C_{35}H_{27}N_3O_7$	602.1927	602.1922
**(*L*)-His**	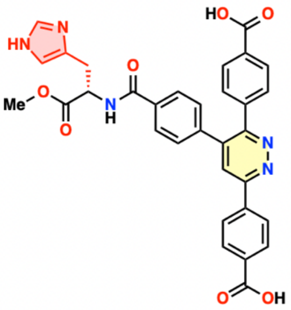	$C_{32}H_{25}N_5O_7$	592.1832	592.1808

S.5.7. THERMOGRAVIMETRIC ANALYSIS (TGA)

Samples were activated in a vacuum oven at 120ºC overnight before the analysis. Changes on the weight loss curve show the compositional changes in the frameworks with larger mass losses, due to the included organic residues.

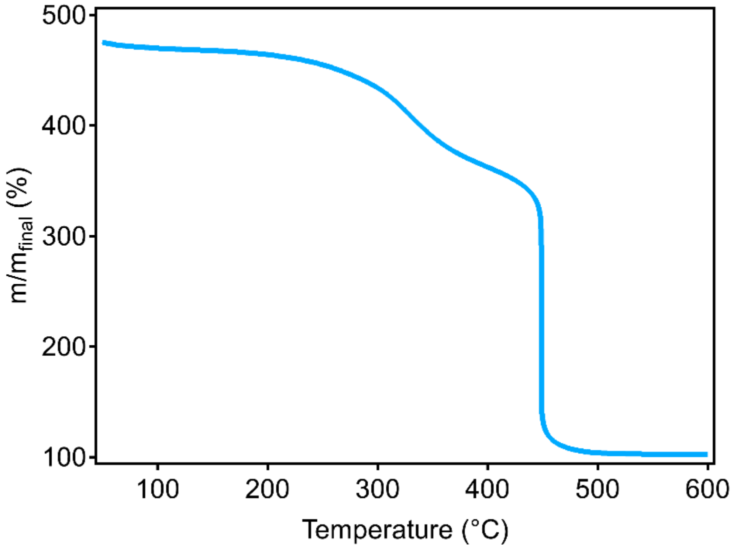

Figure S18. TGA analysis of UiO-68-PZDC-(*L*)-Ala.

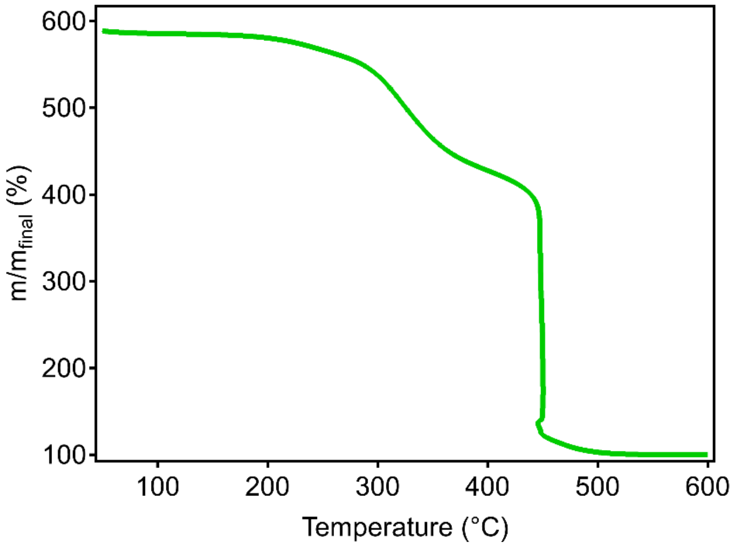

Figure S19. TGA analysis of UiO-68-PZDC-(*L*)-Phe.

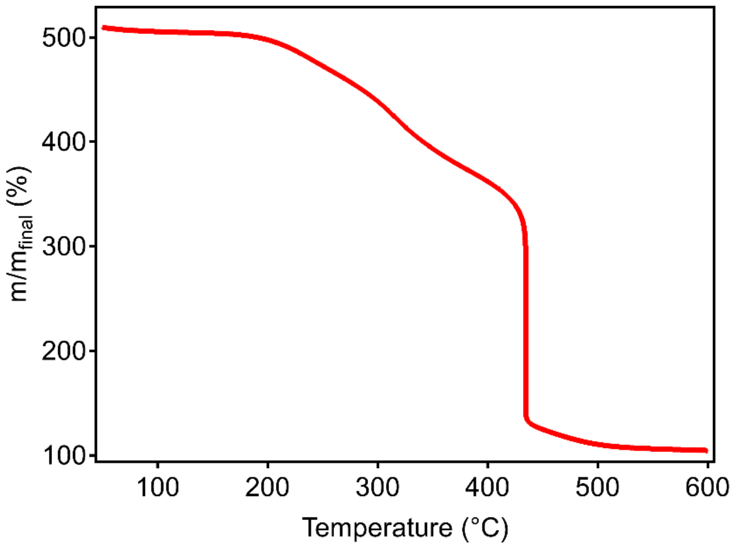

Figure S20. TGA analysis of UiO-68-PZDC-(*L*)-His.

S.5.8. CIRCULAR DICHROISM (CD)

Samples were dispersed in methanol (1 mg·mL^{-1}) and introduced in a quartz cuvette for their measurement. The materials' spectra were compared with the recorded spectra for the corresponding amino acid starting reagents (methyl ester forms, H-(*L*)-AA-OMe; 0.25 mg·mL^{-1}) methanolic solutions. The high-tension voltage plots with values below 700 V assure reliable data.

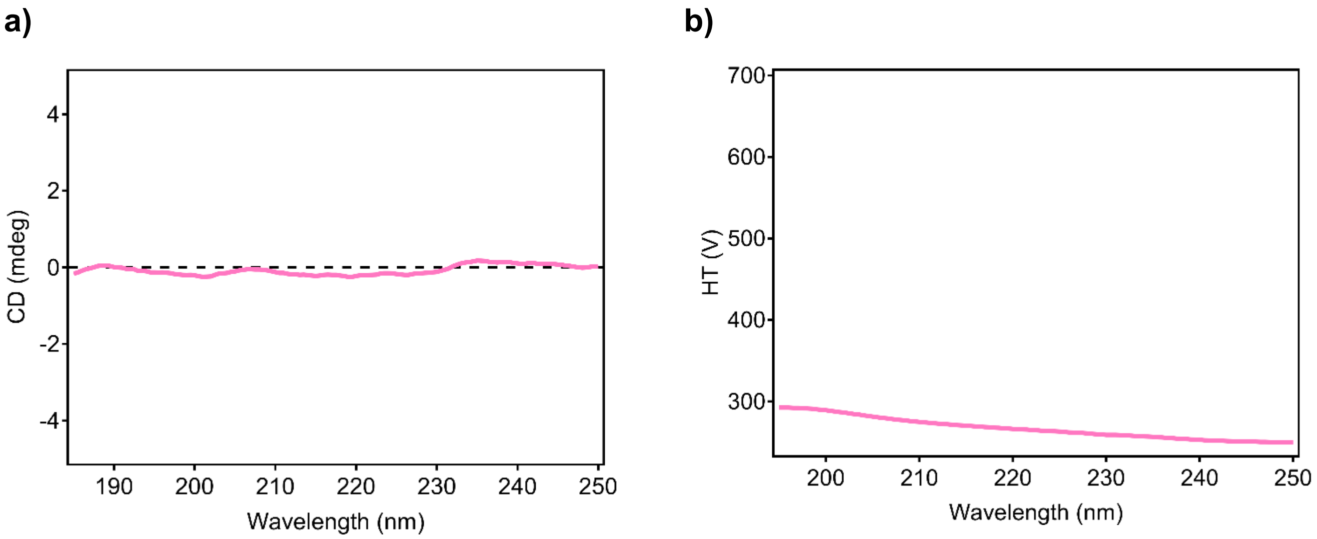

Figure S21. a) Circular dichroism analysis of UiO-68-TZDC and b) high-tension voltage plot of the measure. No signals can be observed, which indicates the achiral nature of the pristine material.

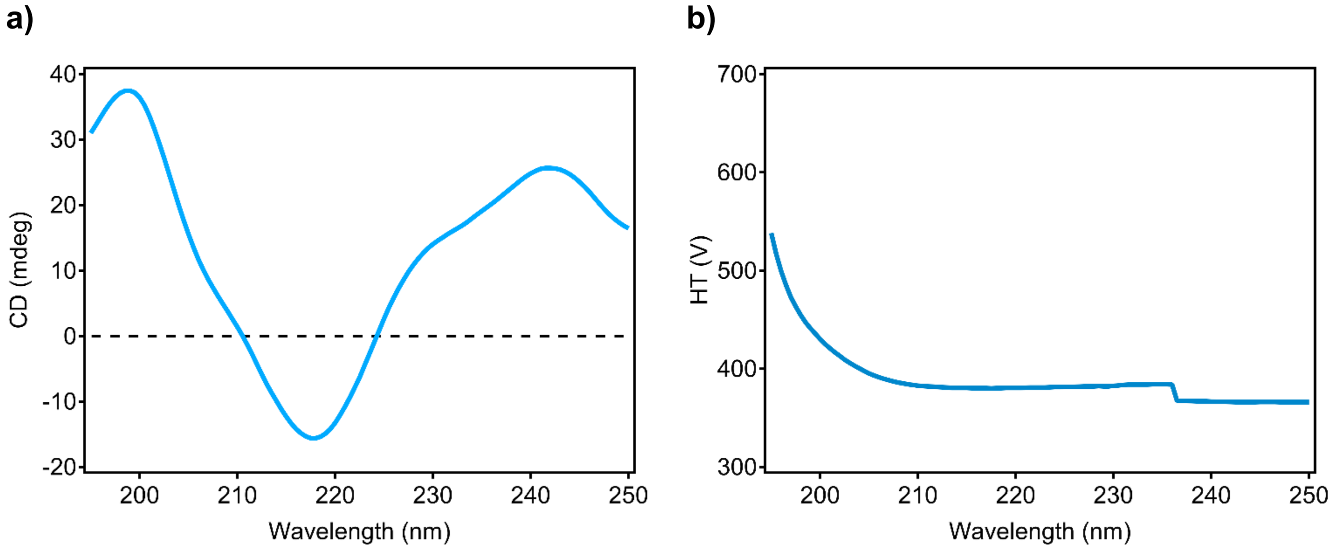

Figure S22. a) Circular dichroism analysis of UiO-68-PZDC-(*L*)-Ala and b) high-tension voltage plot of the measure.

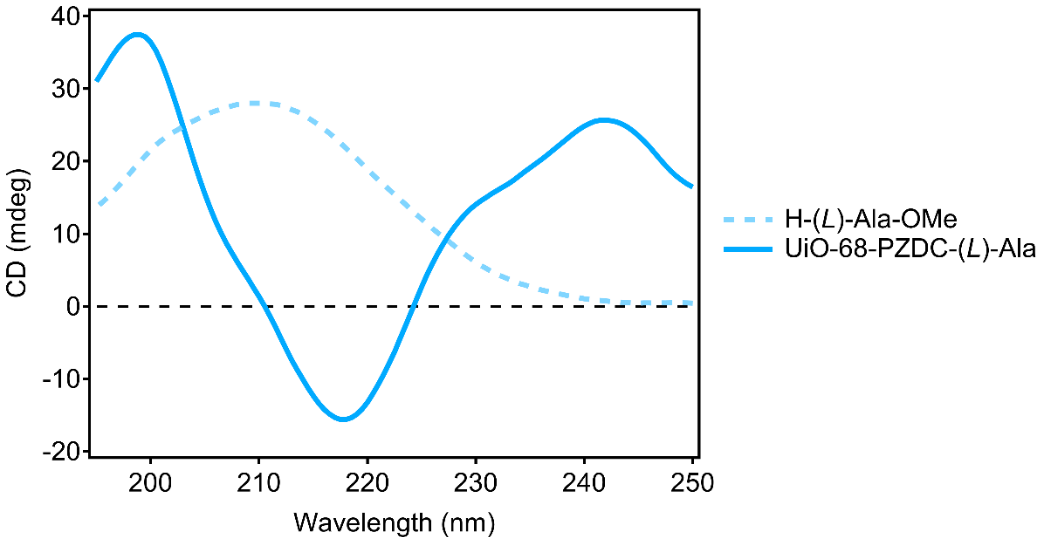

Figure S23. Comparison of the normalized circular dichroism spectra of H-(*L*)-Ala-OMe and UiO-68-PZDC-(*L*)-Ala.

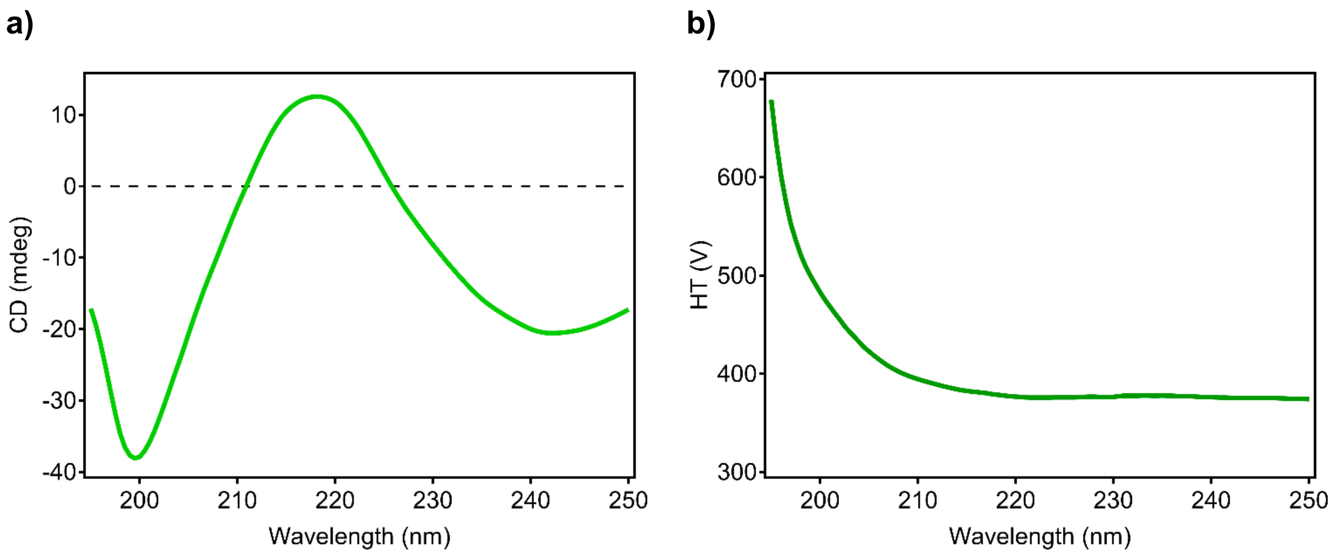

Figure S24. a) Circular dichroism analysis of UiO-68-PZDC-(*L*)-Phe and b) high-tension voltage plot of the measure.

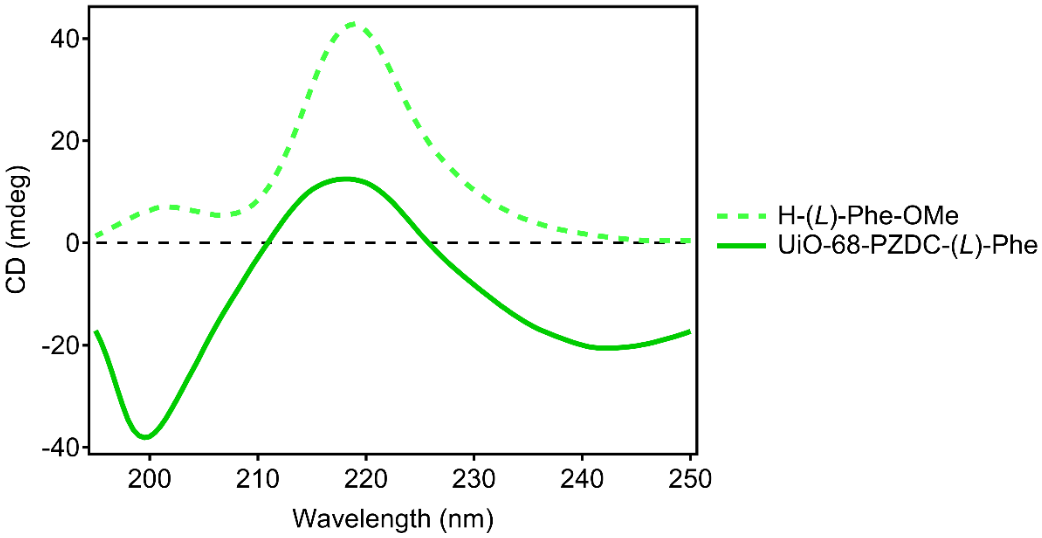

Figure S25. Comparison of the normalized circular dichroism spectra of H-(*L*)-Phe-OMe and UiO-68-PZDC-(*L*)-Phe.

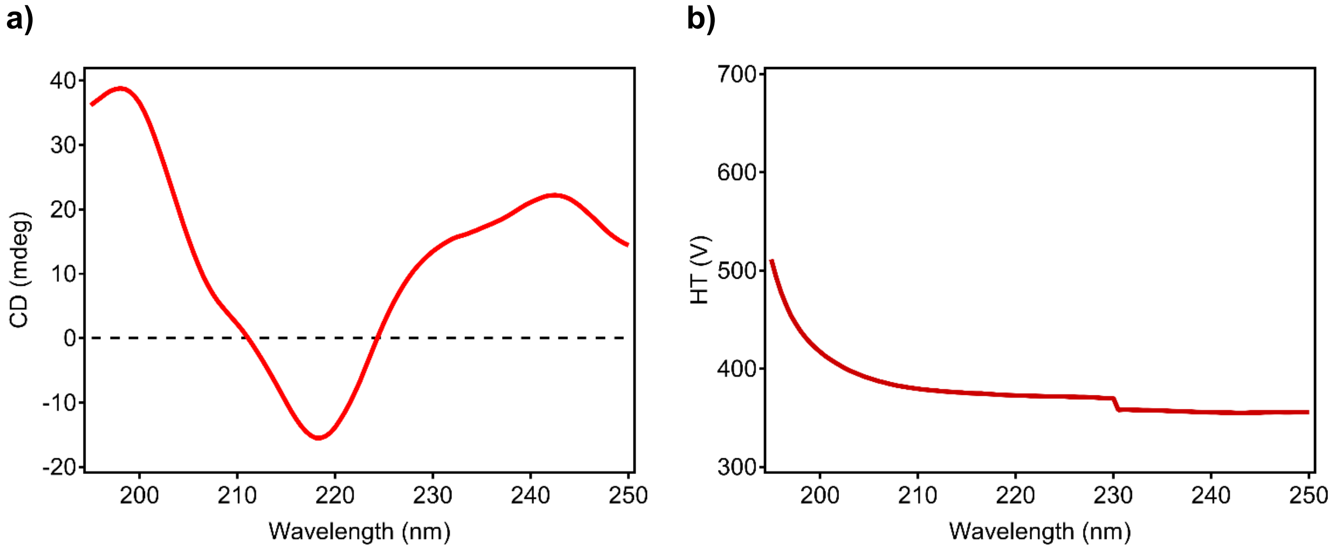

Figure S26. a) Circular dichroism analysis of UiO-68-PZDC-(*L*)-His and b) high-tension voltage plot of the measure.

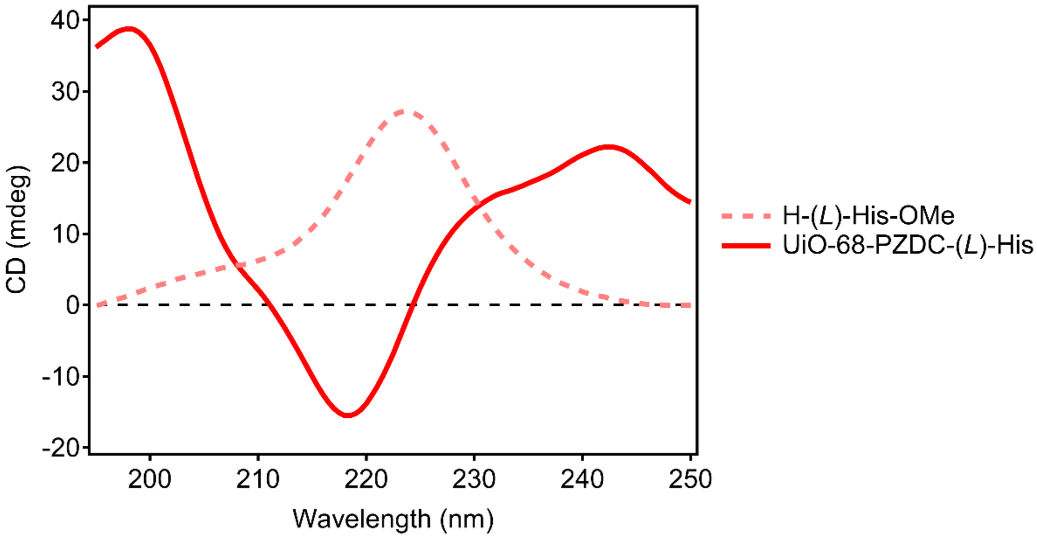

Figure S27. Comparison of the normalized circular dichroism spectra of H-(*L*)-His-OMe and UiO-68-PZDC-(*L*)-His.

S.6. CHIRAL DRUG SEPARATIONS

S.6.1. OPTIMIZATION OF THE METHODOLOGY

We started by optimizing the drug concentration and temperature of the separations. We report the process for UiO-68-PZDC-(*L*)-His, as it was the material which showed the larger supernatant enantiomeric excess (*ee*) values.

General procedure for optimization studies

The enantioselective adsorption of cetirizine was studied by immersing $7.5 \cdot 10^{-4}$ mmol of evacuated material in 1 mL of an ethanolic solution of the racemic drug in a 1.5 mL Eppendorf tube. Then, the tube was shaken at 600 rpm in an Eppendorf ThermoMixer® C device with temperature control for 16 hours. The tube was afterwards centrifugated and the supernatant was collected. It was finally filtrated with a 0.2 µm PTFE filter and analysed via chiral HPLC to obtain the supernatant *ee* (%) values of each essay (see **Section S.6.2** for details).

Table S5. Survey of the conditions used for the enantioselective adsorption of cetirizine in UiO-68-PZDC-(*L*)-His.

Entry	Concentration (mM)	Temperature (ºC)	*ee* (%)†
**1**	0.4	25	71 % (*R*)
**2**	1	25	80 % (*R*)
**3**	2	25	42 % (*R*)
**4**	1	10	59 % (*R*)
**5**	1	35	62 % (*R*)

† Determined by HPLC (letters in brackets indicate most concentrated enantiomer in the supernatant).

By comparing the peak areas before and after separation, we determined the *ee* values within the material. Our analysis focused on entries 1–3 from **Table S5**, which reveal an increase in *ee* of up to 83%, underscoring the strong influence of the initial conditions on the overall process.

Table S6. Survey of the conditions used for the enantioselective adsorption of cetirizine in UiO-68-PZDC-(*L*)-His.

Entry	Concentration (mM)	ee_{inside} (%)†
**1**	0.4	59 % (*S*)
**2**	1	82 % (*S*)
**3**	2	83 % (*S*)

† Determined by comparison between peak areas before and after separation (letters in brackets indicate most concentrated enantiomer in the supernatant).

S.6.2. COMPARISON BETWEEN MATERIALS

General procedure for comparison studies

The enantioselective adsorption of cetirizine was studied by immersing $7.5 \cdot 10^{-4}$ mmol of evacuated material in 1 mL of a 1 mM ethanolic solution of the racemic drug in a 1.5 mL Eppendorf tube. Then, the tube was shaked at 600 rpm in an Eppendorf ThermoMixer® C device at 25 ºC for 16 hours. The tube was afterwards centrifugated and the supernatant was collected. It was finally filtrated with a 0.2 µm PTFE filter and analysed via chiral HPLC to obtain the *ee* (%) values of each essay (see **Section S.6.2** for details).

Table S7. Survey of the enantioselective adsorption experiments of cetirizine with all the materials.

Entry	Material	*ee* (%)†
**1**	UiO-68-TZDC	< 1 %
**2**	UiO-68-PZDC-(*L*)-Ala	< 1 %
**3**	UiO-68-PZDC-(*L*)-Phe	< 1 %
**4**	UiO-68-PZDC-(*L*)-His	80 % (*R*)

† Determined by HPLC (letters in brackets indicate most concentrated enantiomer in the supernatant).

Leaching test under separation conditions

To test the leaching of the grafted chains under the separation conditions, we tested the functionalized materials with control experiments via UV-Vis spectroscopy of the ethanol supernatants (**Figure S28**). The experiments confirm the absence of leaching, highlighting the excellent anchoring stability of the grafted groups.

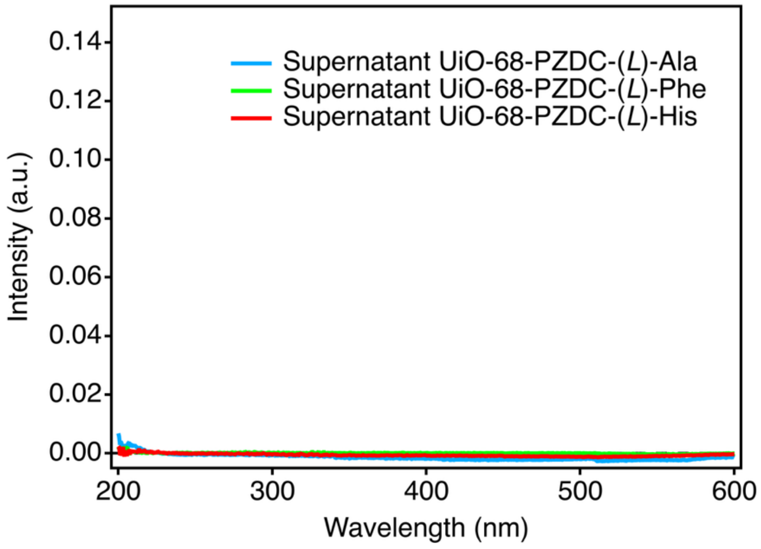

Figure S28. UV-Vis spectra of the supernatants after the anchored chains leaching test.

Characterization of the materials after their use

To test the crystallinity of the material after its use in the separation experiments, we checked its PXRD patterns and crystal morphology via SEM. We interchanged the samples with *n*-hexane before the measurements.

As shown in the next pages, both PXRD and SEM show the great retention of crystallinity from the samples after their use.

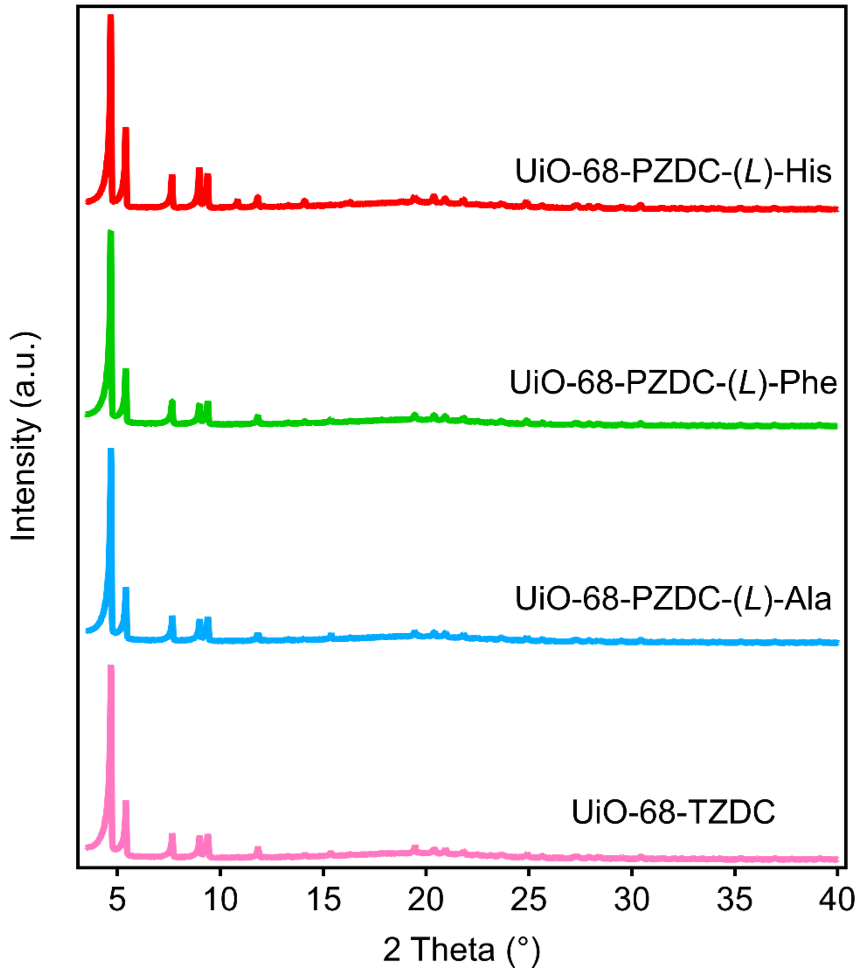

Figure S29. Comparison of the normalized PXRD patterns from the different materials after the separation experiments (λ = 1.5406 Å).

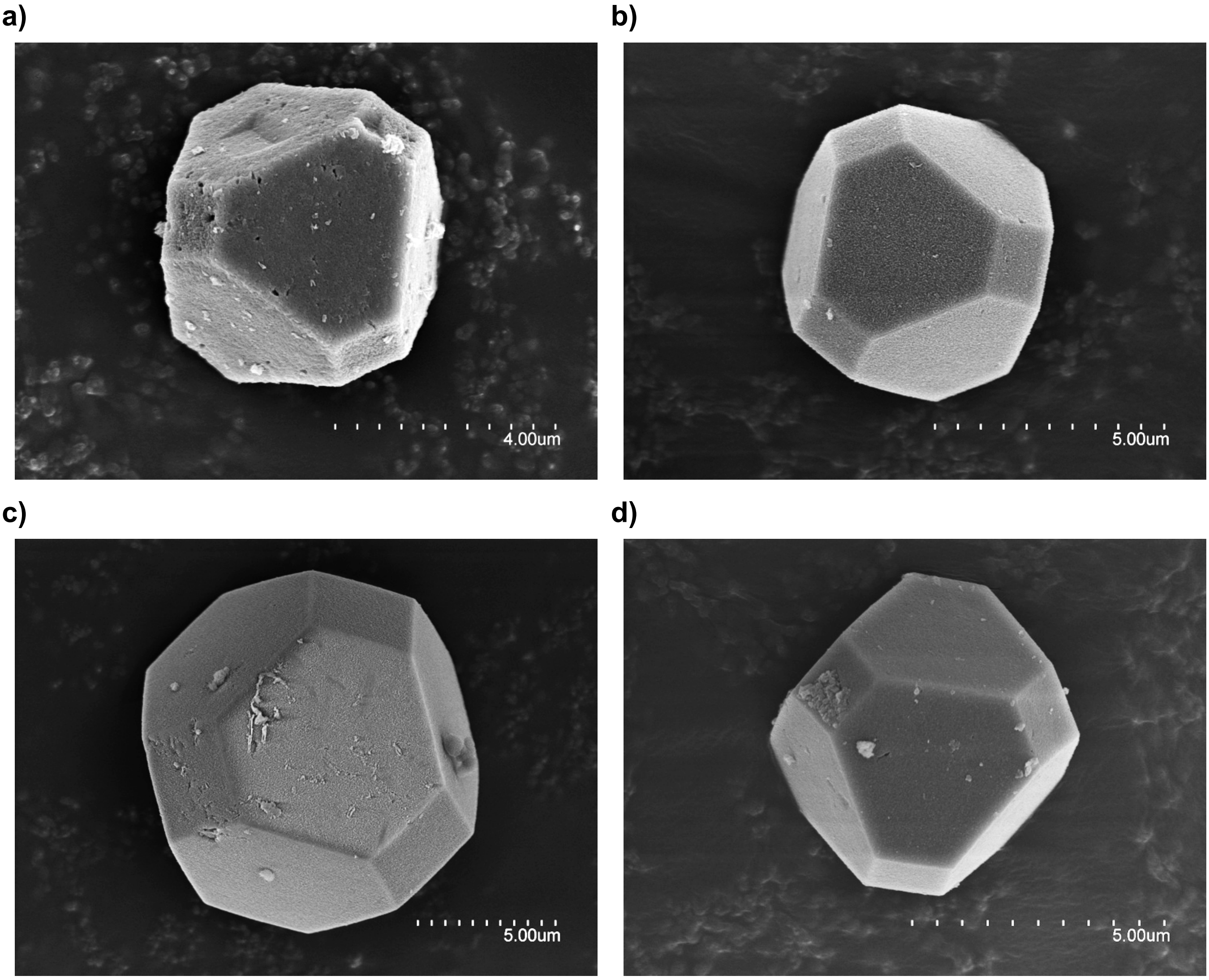

Figure S30. SEM images of a) UiO-68-TZDC; b) UiO-68-PZDC-(*L*)-Ala; c) UiO-68-PZDC-(*L*)-Phe; d) UiO-68-PZDC-(*L*)-His and after the separation experiments.

S.6.3. RECYCLABILITY TESTS

To test the recyclability of UiO-68-PZDC-(*L*)-His as enantioselective adsorbent of cetirizine, we performed the process three consecutive times with the same sample. Between each essay, we thoroughly washed the material with fresh methanol. Specifically, we added 1 mL of methanol to the Eppendorf tube and shaked it at 600 rpm for 2 hours. Then, we centrifugated the tube, removed the supernatant and repeated the process a total of four times.

As shown below, no significant change on the enantiomeric excess values were detected between the different cycles.

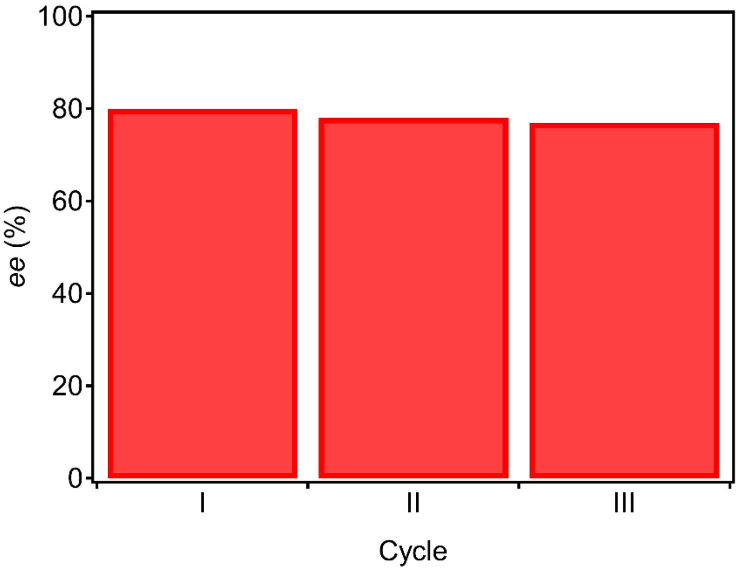

Figure S31. Enantiomeric excess values from the recycling experiments of UiO-68-PZDC-(*L*)-His as enantioselective adsorbent of cetirizine.

Characterization of the material after its use

To test the crystallinity of the material after its use in the separation experiments, we checked its PXRD patterns and crystal morphology via SEM. We interchanged the samples with *n*-hexane before the measurements.

As shown in the next page, both PXRD and SEM show the great retention of crystallinity from the samples after their use.

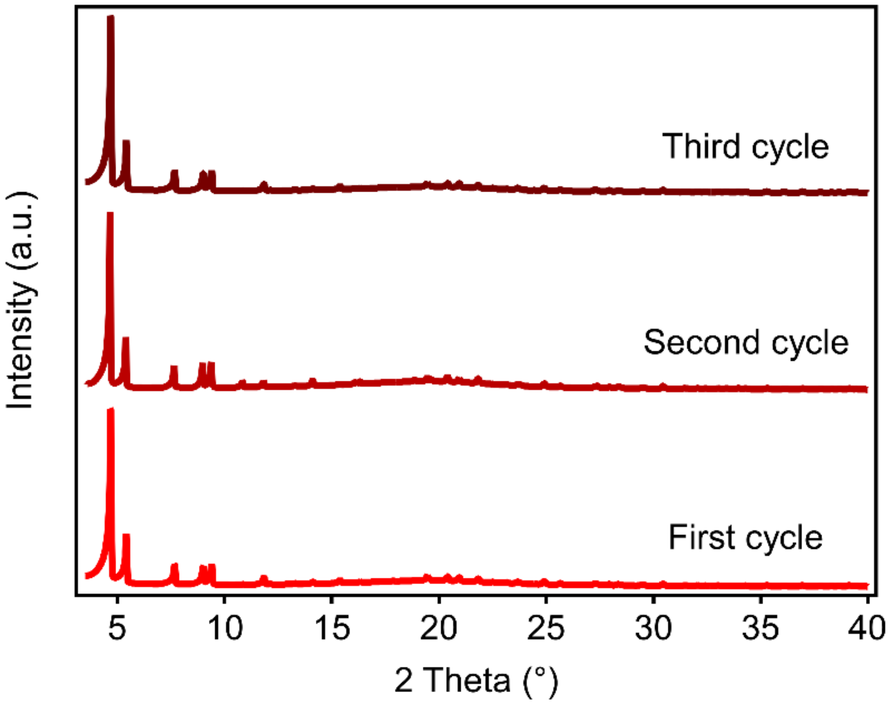

Figure S32. Comparison of the normalized PXRD patterns of UiO-68-PZDC-(*L*)-His after the three different cycles of separation experiments.

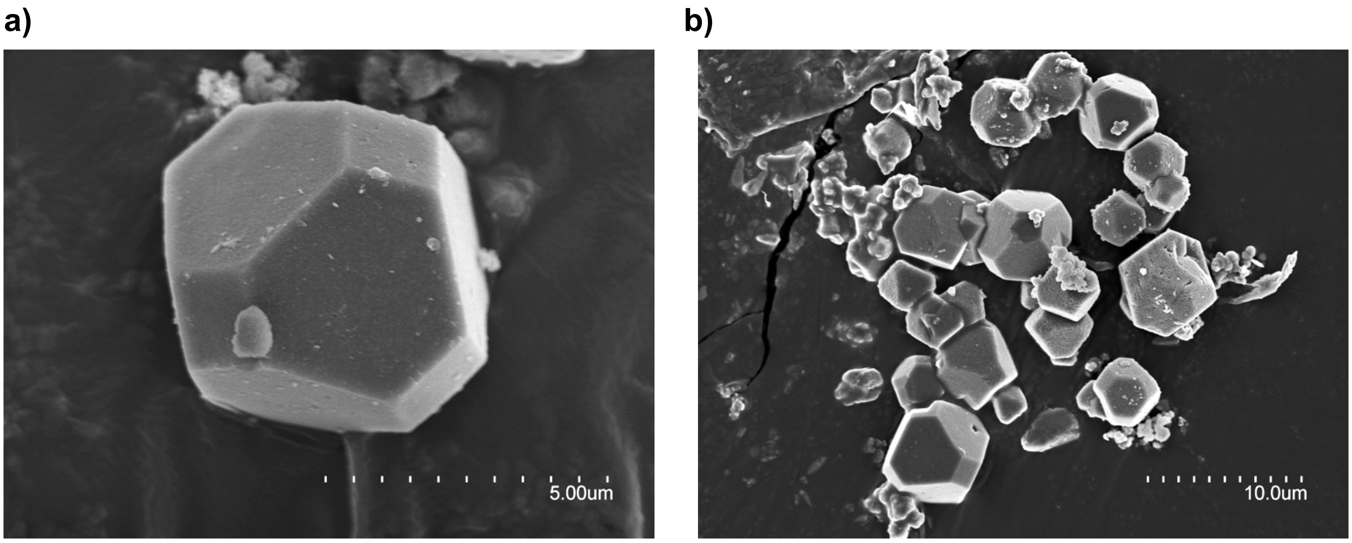

Figure S33. Scanning electron microscopy images of UiO-68-PZDC-(*L*)-His after third cycle of separation with a) higher and b) lower magnification.

S.6.4. HPLC ANALYSIS

We used normal phase conditions, with a mobile phase consisting of 90% *n*-hexane + 10% isopropyl alcohol + 0.3% trifluoroacetic acid + 0.1 % triethylamine and a flow rate of 1 mL·min^{-1}. The injection oven temperature was 40 ºC. The sample injection volume was 10 µL. The chosen wavelength of UV analysis was 230 nm. Between each essay, a blank was measured to ensure that the column was not poisoned with the drug, ensuring reliable results from the analyses. The chromatograms show two peaks at retention times around 11.8 min and 16.0 min which correspond to the two different enantiomers of cetirizine.

To calculate the enantiomeric excess (*ee*), we used the following formula:

$$ee\ (\%) = \frac{|A_S - A_R|}{A_S + A_R} \times 100$$

where A_S is the peak area of (*S*)-cetirizine and A_R is the peak area of (*R*)-cetirizine.

We started measuring the mother ethanolic solution of 1 mM racemic cetirizine in which we immersed the different materials to perform the separation experiments.

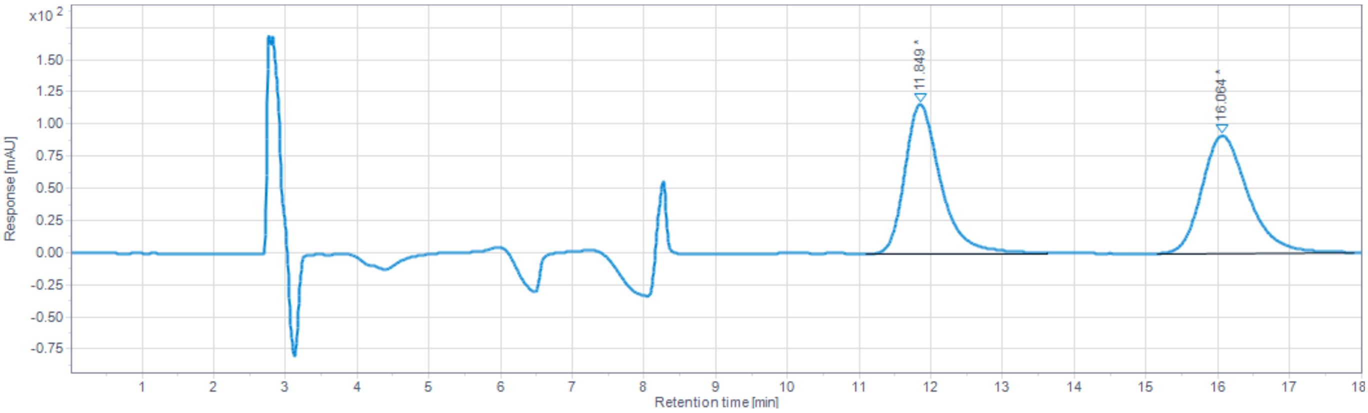

Peak number	Retention time (min)	Start time of the peak (min)	End time of the peak (min)	Area (mAU·s)	Area fraction (%)
**1**	11.849	11.093	13.617	4063.711	49.997
**2**	16.064	15.153	17.894	4064.208	50.003

Figure S34. HPLC analysis of 1 mM racemic cetirizine. Enantiomeric excess calculated from the peaks area: *ee* = < 1 %

We continued assigning each peak to the corresponding enantiomer of cetirizine. To do so, we measured an optically pure sample of (*R*)-cetizirine. The chromatogram shows the disappearance of the first peak. Therefore, the peak around 11.8 min corresponds to (*S*)-cetirizine, while the peak around 16.0 min corresponds to (*R*)-cetirizine.

The main chromatograms analysis corresponding to the entries from **Table S.6** can be found in the following pages.

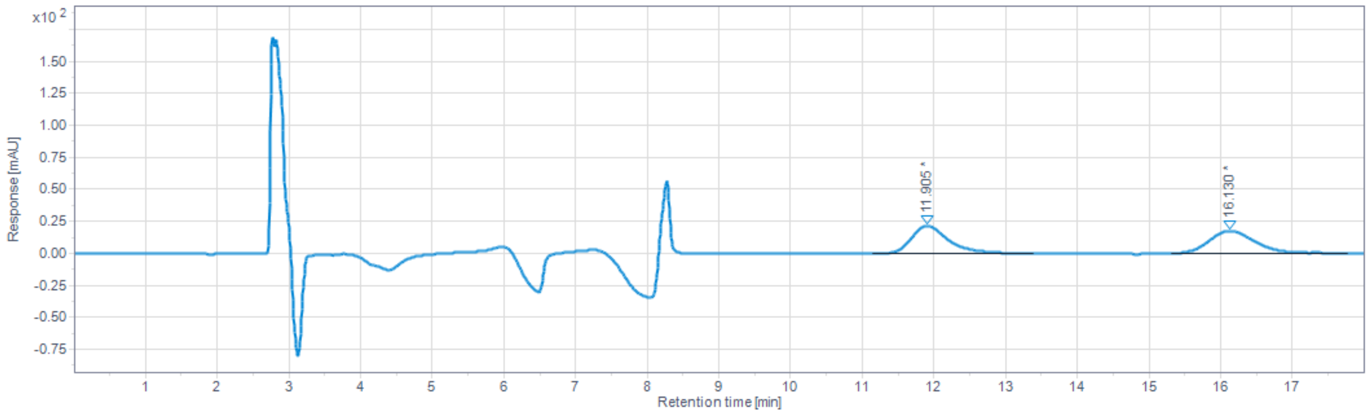

Peak number	Retention time (min)	Start time of the peak (min)	End time of the peak (min)	Area (mAU·s)	Area fraction (%)
**1**	11.905	11.156	13.376	782.608	49.042
**2**	16.130	15.325	17.761	811.550	50.908

Figure S35. HPLC analysis of the separation experiment with UiO-68-TZDC. Enantiomeric excess calculated from the peaks area: *ee* = < 1 %.

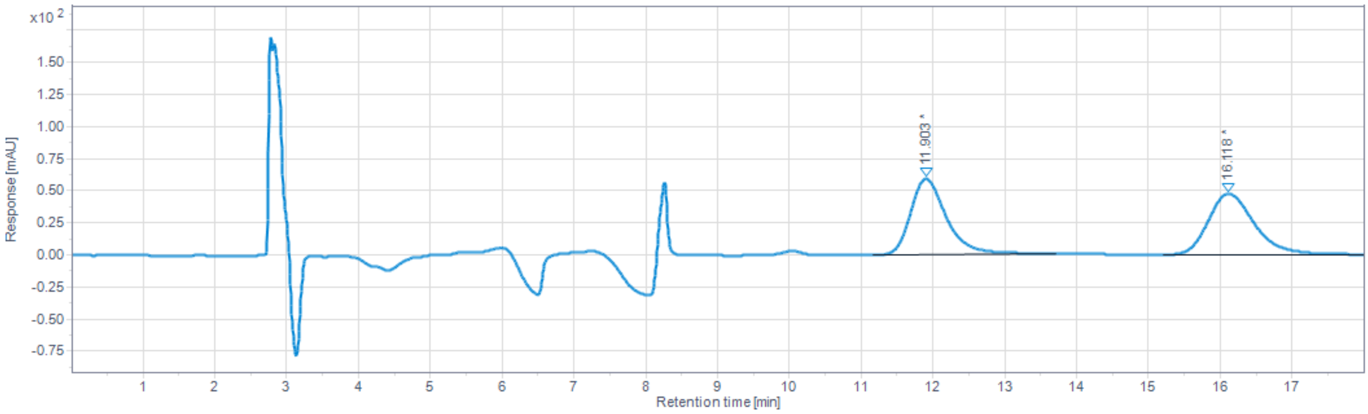

Peak number	Retention time (min)	Start time of the peak (min)	End time of the peak (min)	Area (mAU·s)	Area fraction (%)
**1**	11.903	11.158	13.702	2051.237	49.253
**2**	16.118	15.200	17.998	2113.424	50.747

Figure S36. HPLC analysis of the separation experiment with UiO-68-PZDC-(*L*)-Ala. Enantiomeric excess calculated from the peaks area: *ee* = < 1 %.

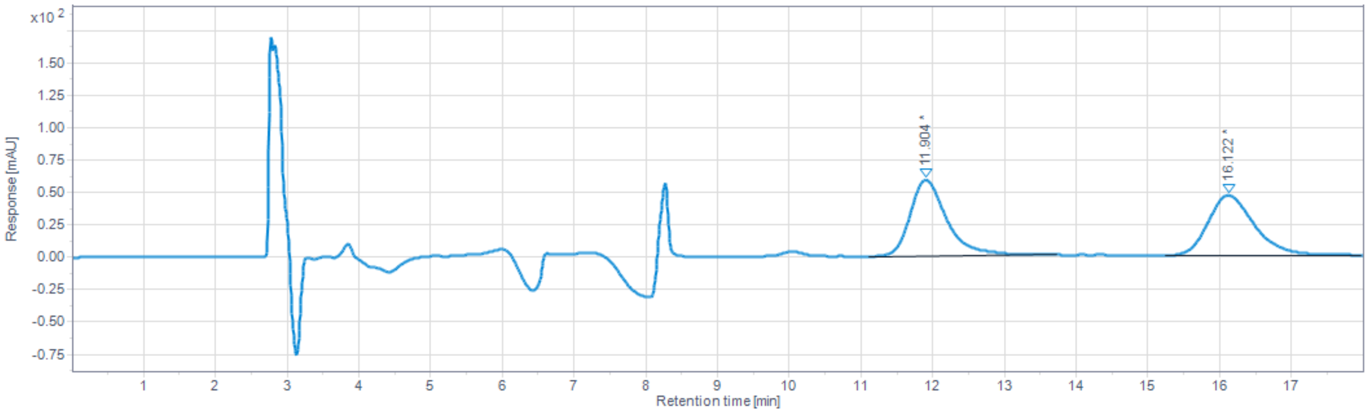

Peak number	Retention time (min)	Start time of the peak (min)	End time of the peak (min)	Area (mAU·s)	Area fraction (%)
**1**	11.904	11.121	13.738	2081.615	49.789
**2**	16.122	15.240	17.964	2099.218	50.211

Figure S37. HPLC analysis of the separation experiment with UiO-68-PZDC-(*L*)-Phe. Enantiomeric excess calculated from the peaks area: *ee* = < 1 %.

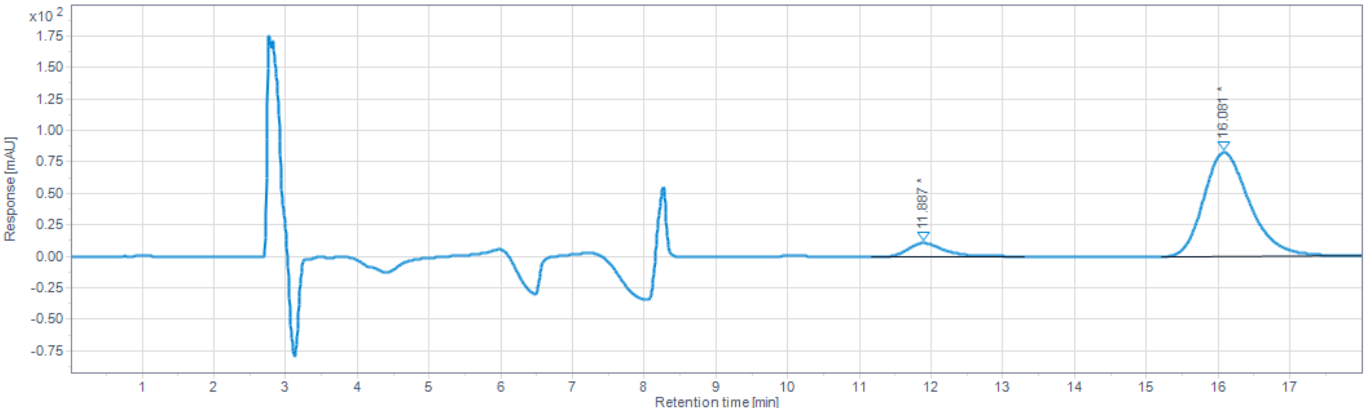

Peak number	Retention time (min)	Start time of the peak (min)	End time of the peak (min)	Area (mAU·s)	Area fraction (%)
**1**	11.887	11.159	13.289	416.802	10.117
**2**	16.081	15.202	17.999	3702.974	89.883

Figure S38. HPLC analysis of the separation experiment with UiO-68-PZDC-(*L*)-His. Enantiomeric excess calculated from the peaks area: *ee* = 80 % (*R*).

S.6.5. COMPARISON WITH OTHER CHIRAL MOFs

To place the performance of our material into a broader context, we conducted a survey of the most representative examples of chiral drug separation achieved in chiral MOFs, organizing them according to the separation protocol used.

Table S8. Representative examples of enantioselective drug separation in MOFs.

Protocol	Drug	MOF	ee (%)	Ref.
Adsorption	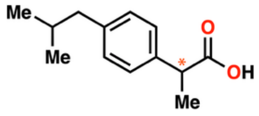 Ibuprofen	HMOF-1	18 %	6
	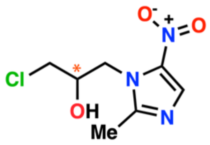 Ornidazole	Eu^{3+}@HMOF-1	86 %	7
	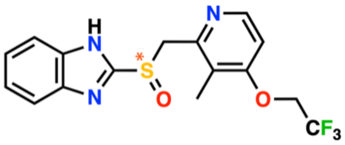 Lansoprazole	$[Cd_2\mathbf{L}(DMF)_2(H_2O)]\cdot DMF\cdot 3H_2O$	91 %	8
	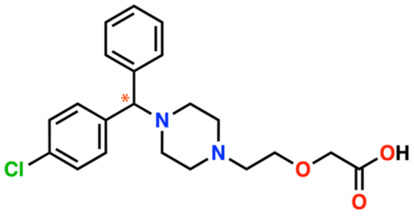 Cetirizine	UiO-68-PZDC-(*L*)-His	80 %	This work
	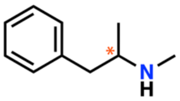 Methamphetamine	Cu(GHG)	30 %	9
Solid phase extraction column	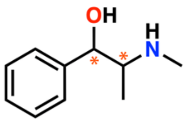 Ephedrine		54 %	

Protocol	Drug	MOF	ee (%)	Ref.
Membrane separation	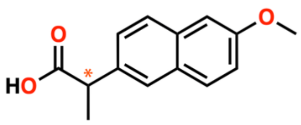<br>Naproxen	MOF-808-(*L*)-His	93 %	10
		MOF-808-(*L*)-Ala	95 %	
Chromatography column	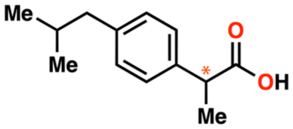<br>Ibuprofen	TAMOF-1	99 %	11
	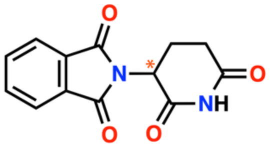<br>Thalidomide		99 %	
	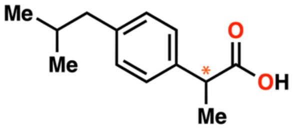<br>Ibuprofen	$[(Zn_4O)_2(\mathbf{L})_6(bpy)_3]$	94 %	12
Reverse phase HPLC column	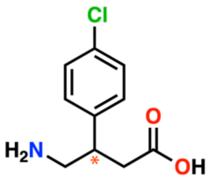<br>Baclofen	$[Zr_6O_4(OH)_8(H_2O)_4(\mathbf{L^1})_2]$	99%	13
	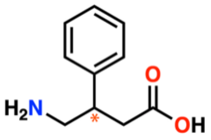<br>Phenibut		99%	
	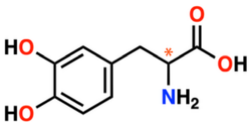<br>Dopa		99%	
	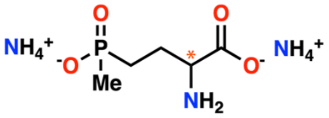<br>Phosphinothricin		99%	

S.6.6. ANALYSIS OF THE ADSORPTION CAPACITIES

With the aim of evaluating further the utility of UiO-68-PZDC-(*L*)-His as a chiral adsorbent of cetirizine, we analysed its adsorption capacity. Based on the area reduction for each enantiomer, we calculated the loading for each molecule within the MOF cavities.

Table S9. Summary of relevant data for the analysis of adsorption capacities.

Entry	Material	(*S*)-cetirizine uptake (mol/mol)	(*R*)-cetirizine uptake (mol/mol)
**1**	UiO-68-TZDC	1.07	1.08
**2**	UiO-68-PZDC-(*L*)-Ala	0.64	0.66
**3**	UiO-68-PZDC-(*L*)-Phe	0.64	0.65
**4**	UiO-68-PZDC-(*L*)-His	0.11	1.20

When comparing our results with those reported in the literature for systems employing similar experimental formats,[12,14] we find that our capacities fall within a comparable range. Importantly, the trends observed across our series provide additional insight into the structural consequences of the PSM. The pristine framework displays the highest overall uptake, while all three functionalized materials show similarly reduced capacities. This behavior is consistent with partial pore occupation imposed by the grafted chiral arms. Notably, only the histidine–modified framework exhibits an enantioenriched response, demonstrating that although pore volume decreases in all cases, histidine uniquely generates an internal environment capable of discriminating between the two enantiomers.

S.6.7. TESTING THE MATERIALS WITH OTHER CHIRAL DRUGS

With the aim of establishing generality and application potential of our materials, we tested their separation performance with other chiral drugs. Once again, we chose large molecules with conformational flexibility and groups capable of non-covalent interactions.

Briefly, $7.5 \cdot 10^{-4}$ mmol of evacuated material in 1 mL of an ethanolic solution of the racemic drug in a 1.5 mL Eppendorf tube. Then, the tube was shaken at 600 rpm in an Eppendorf ThermoMixer® C device with temperature control for 16 hours. The tube was afterwards centrifugated and the supernatant was collected. It was finally filtrated with a 0.2 µm PTFE filter and analysed via chiral HPLC to obtain the supernatant *ee* (%) values of each essay (**Table S10**).

Table S10. Survey of the enantioselective adsorption experiments of cetirizine with all the materials.

Entry	Drug	Material	*ee* (%)†
**1**	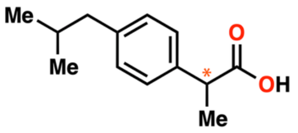 Ibuprofen	UiO-68-TZDC	< 1 %
**2**		UiO-68-PZDC-(*L*)-Ala	< 1 %
**3**		UiO-68-PZDC-(*L*)-Phe	< 1 %
**4**		UiO-68-PZDC-(*L*)-His	< 1 %
**5**	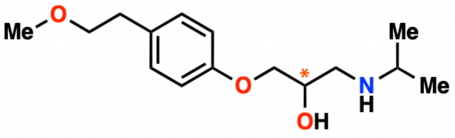 Metoprolol	UiO-68-TZDC	< 1 %
**6**		UiO-68-PZDC-(*L*)-Ala	< 1 %
**7**		UiO-68-PZDC-(*L*)-Phe	< 1 %
**8**		UiO-68-PZDC-(*L*)-His	< 1 %
**9**	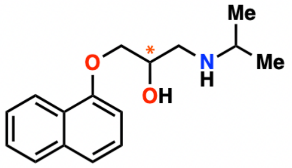 Propranolol	UiO-68-TZDC	< 1 %
**10**		UiO-68-PZDC-(*L*)-Ala	< 1 %
**11**		UiO-68-PZDC-(*L*)-Phe	< 1 %
**12**		UiO-68-PZDC-(*L*)-His	< 1 %

† Determined by HPLC (letters in brackets indicate most concentrated enantiomer in the supernatant).

HPLC analysis details

Ibuprofen: We used normal phase conditions, with a mobile phase consisting of 99% *n*-hexane + 1% isopropyl alcohol + 0.1% trifluoroacetic acid and a flow rate of 1 $mL \cdot min^{-1}$. The injection oven temperature was 25 ºC. The sample injection volume was 5 µL. The chosen wavelength of UV analysis was 254 nm. Between each essay, a blank was measured to ensure that the column was not poisoned with the drug, ensuring reliable results from the analyses. The chromatograms show two peaks at retention times around 11.7 min and 20.1 min which correspond to the two different enantiomers of ibuprofen.

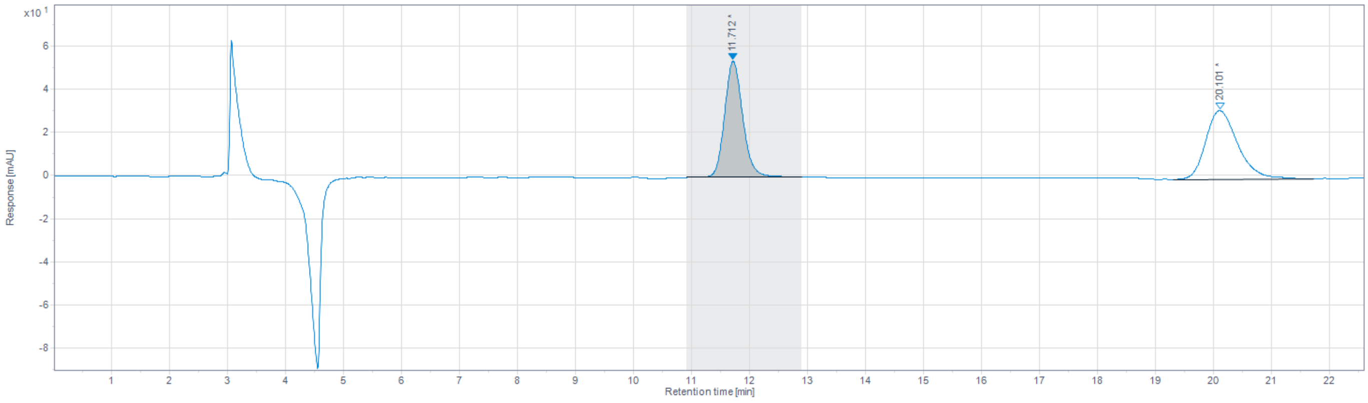

Peak number	Retention time (min)	Start time of the peak (min)	End time of the peak (min)	Area (mAU·s)	Area fraction (%)
**1**	11.712	10.917	12.892	1178.649	49.133
**2**	20.101	19.302	21.724	1220.258	50.867

Figure S39. HPLC analysis of the separation experiment with UiO-68-PZDC-(*L*)-His and ibuprofen. Enantiomeric excess calculated from the peaks area: *ee* = < 1 %.

Metoprolol: We used normal phase conditions, with a mobile phase consisting of 90% *n*-hexane + 10% ethanol + 0.1% diethylamine and a flow rate of 1 mL·min^{-1}. The injection oven temperature was 25 ºC. The sample injection volume was 5 µL. The chosen wavelength of UV analysis was 230 nm. Between each essay, a blank was measured to ensure that the column was not poisoned with the drug, ensuring reliable results from the analyses. The chromatograms show two peaks at retention times around 5.5 min and 7.8 min which correspond to the two different enantiomers of metoprolol.

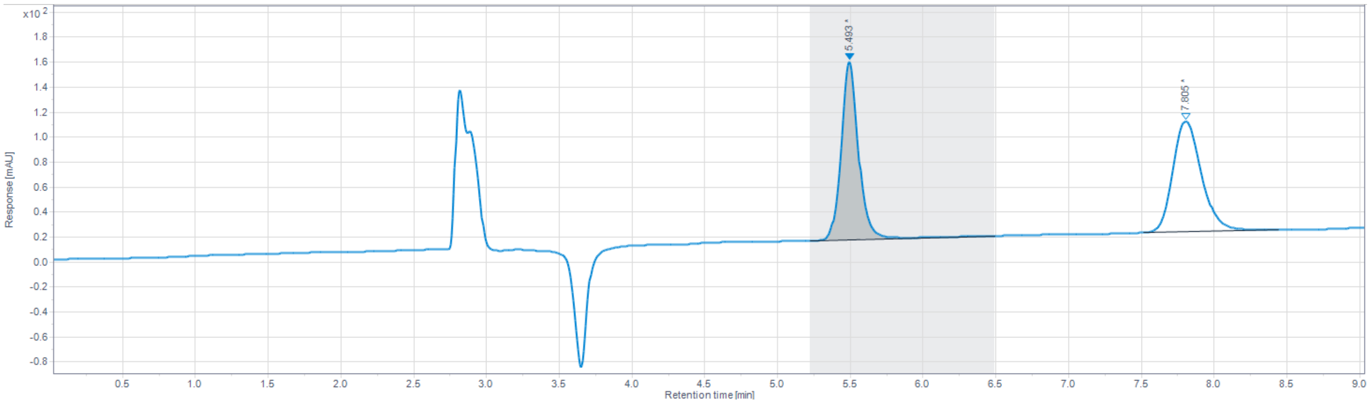

Peak number	Retention time (min)	Start time of the peak (min)	End time of the peak (min)	Area (mAU·s)	Area fraction (%)
**1**	5.493	5.225	6.490	1188.527	49.606
**2**	7.805	7.515	8.440	1207.404	50.394

Figure S40. HPLC analysis of the separation experiment with UiO-68-PZDC-(*L*)-His and metoprolol. Enantiomeric excess calculated from the peaks area: *ee* = < 1 %.

Propranolol: We used polar phase conditions, with a mobile phase consisting of 100% acetonitrile + 0.1% ethanolamine and a flow rate of 1 mL·min^{-1}. The injection oven temperature was 25 ºC. The sample injection volume was 5 µL. The chosen wavelength of UV analysis was 270 nm. Between each essay, a blank was measured to ensure that the column was not poisoned with the drug, ensuring reliable results from the analyses. The chromatograms show two peaks at retention times around 6.3 min and 7.8 min which correspond to the two different enantiomers of propranolol.

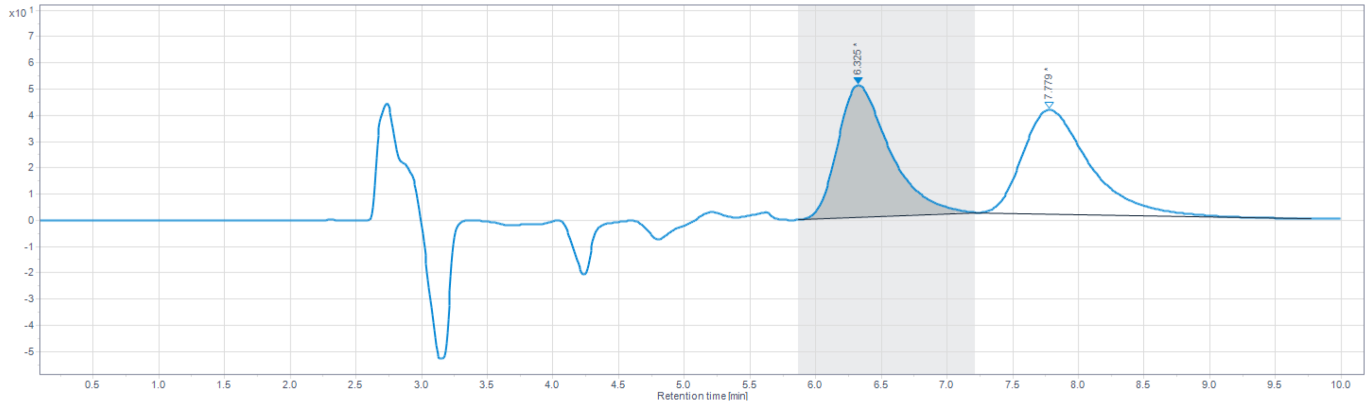

Peak number	Retention time (min)	Start time of the peak (min)	End time of the peak (min)	Area (mAU·s)	Area fraction (%)
**1**	6.325	5.868	7.219	1392.769	49.702
**2**	7.779	7.219	9.774	1409.445	50.298

Figure S41. HPLC analysis of the separation experiment with UiO-68-PZDC-(*L*)-His and propranolol. Enantiomeric excess calculated from the peaks area: *ee* = < 1 %.

S.7. COMPUTATIONAL METHODS

S.7.1. FRAMEWORK MODELS

The structural models were built through a meticulous process using an in-house developed script (codename: *TopoTemp*) in which the original TZDC linkers were replaced by PZDC-(*L*)-X linkers (X = Ala, Phe, His) within the experimentally unit cell of UiO-68-TZDC (CCDC: 2023345) (**Figure S42**). Cell parameters were fixed, but the atomic positions were relaxed using density functional theory (DFT) calculations with the VASP code.[15,16] We applied the GGA with the PBE functional,[17] together with dispersion correction (DFT-D3),[18,19] and the PAW method for valence–core interactions.[20] A kinetic cutoff of 500 eV and Γ-point sampling were used for both UiO-68-PZDC-(*L*)-Ala/Phe/His and (*S*)/(*R*)-cetirizine (CTZ) molecules. After optimization, Monte Carlo simulations in Materials Studio 2017 R2 (adsorption locate module) were performed to identify the most stable adsorption sites for each material. Using this method, different host–guest geometries were obtained for each stereoisomer, corresponding to two configurations for the Phe-containing material, three for His, and five for Ala. These 20 structures were then subjected to energy minimization calculations, employing the same level of theory previously described.

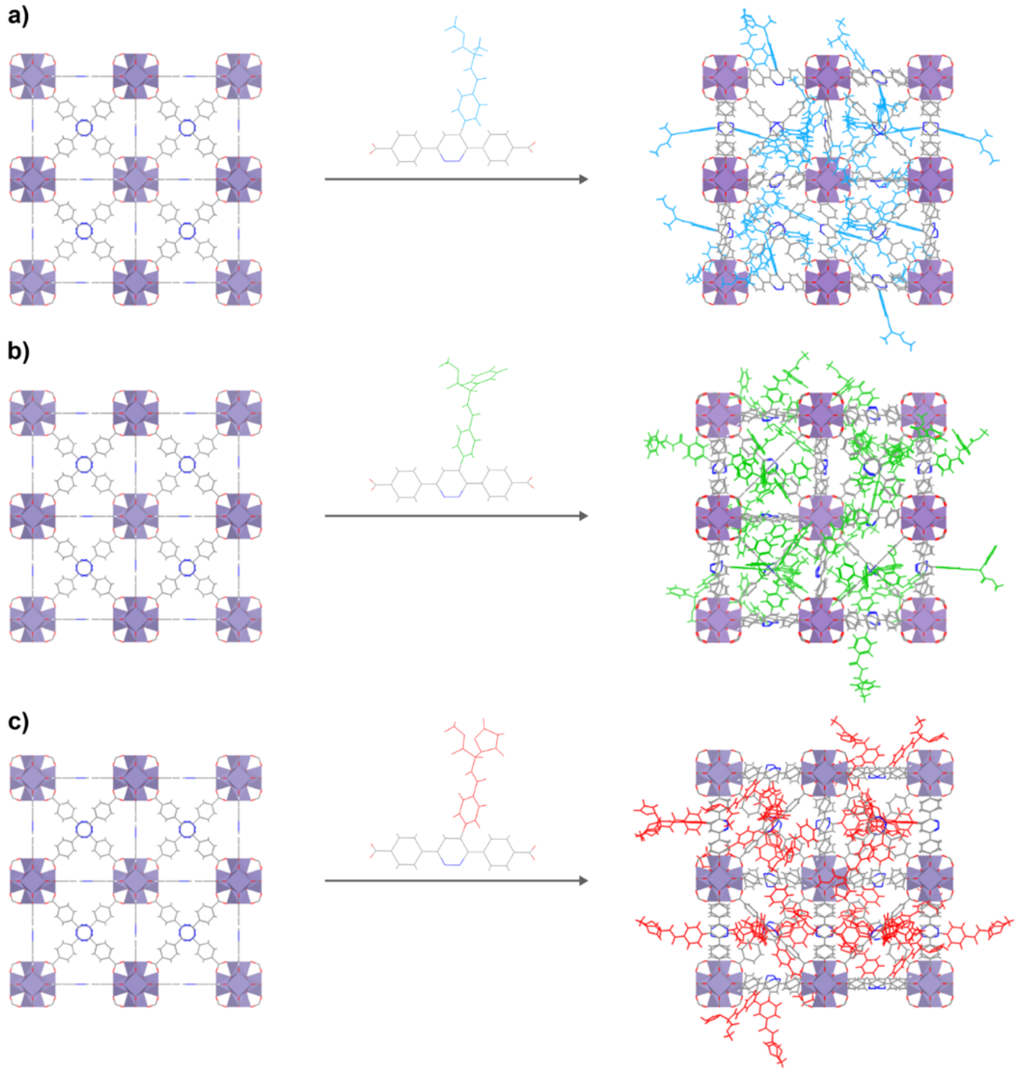

Figure S42. Structural models constructed with the in-house script *TopoTemp*, where the original TZDC linkers were replaced by PZDC-(*L*)-X linkers: a) Ala, b) Phe, and c) His.

S.7.2. INTERACTION ENERGIES

The peptide fragment and cetirizine, with their many degrees of freedom, give rise to a complex potential energy landscape. This makes adequate configurational sampling challenging, yet essential for obtaining results that reproduce the enantioselectivity observed experimentally. Therefore, a combination of molecular dynamics simulations, simulated annealing, and geometry optimizations was employed to explore as many low-energy minima in the conformational space as possible.

Starting from the host-guest geometries optimized at the DFT level, further optimization was carried out with the pGFN-FF model,[21,22] using an algorithm that simulates annealing, as implemented in GULP package (version 6.1.2).[23] The 20 resulting structures served as initial configurations for molecular dynamics simulations in the NVT ensemble. Simulations were first run at 273 K over 400 ps. The temperature was then increased to 288 K, being equilibrated during 200 ps, followed by a 300 ps production run, from which at least 90 different configurations per CTZ stereoisomer have been extracted.

These structures were subsequently energy minimized, also using simulated annealing algorithms, and the Drug-MOF (DM) interaction energy was obtained from single point energy calculations (**Eq. S1**), where $\mathrm{E}(DM)$ is the energy of the UiO-68-PZDC-(L)-X with CTZ molecule adsorbed, $E(D)$ is the energy of CTZ in the gas phase, and $E(M)$ is the energy of the crystal of UiO-68-PZDC-(L)-X. From the 45 most stable geometries per enantiomer, the average interaction energy was calculated for each DM pair (**Eq. S2**, with N = 45, and **Figure S43**), as well as the relative interaction energy between the S and R stereoisomers of CTZ (**Eq. S3** and **Figure 4b** in the main text).

$$\Delta\mathrm{E}^{\mathrm{int}} = \mathrm{E}(DM) - E(D) - E(M) \qquad \textbf{Eq. S1}$$

$$\overline{\Delta\mathrm{E}^{\mathrm{int}}} = \frac{1}{N}\sum_{i=1}^{N} \Delta\mathrm{E}_i^{\mathrm{int}} \qquad \textbf{Eq. S2}$$

$$\Delta\overline{\Delta\mathrm{E}^{\mathrm{int}}} = \overline{\Delta\mathrm{E}^{\mathrm{int}}}(S) - \overline{\Delta\mathrm{E}^{\mathrm{int}}}(R) \qquad \textbf{Eq. S3}$$

In addition to the energetics of the host–guest systems, the preferential adsorption sites were analyzed (**Figure 4a**), using the color scheme shown in **Figure S44**. **Table S8** summarizes previous computational studies on enantioselective drug separation by MOFs, providing an overview of the interaction energies reported, the systems investigated, and the theoretical approaches employed.

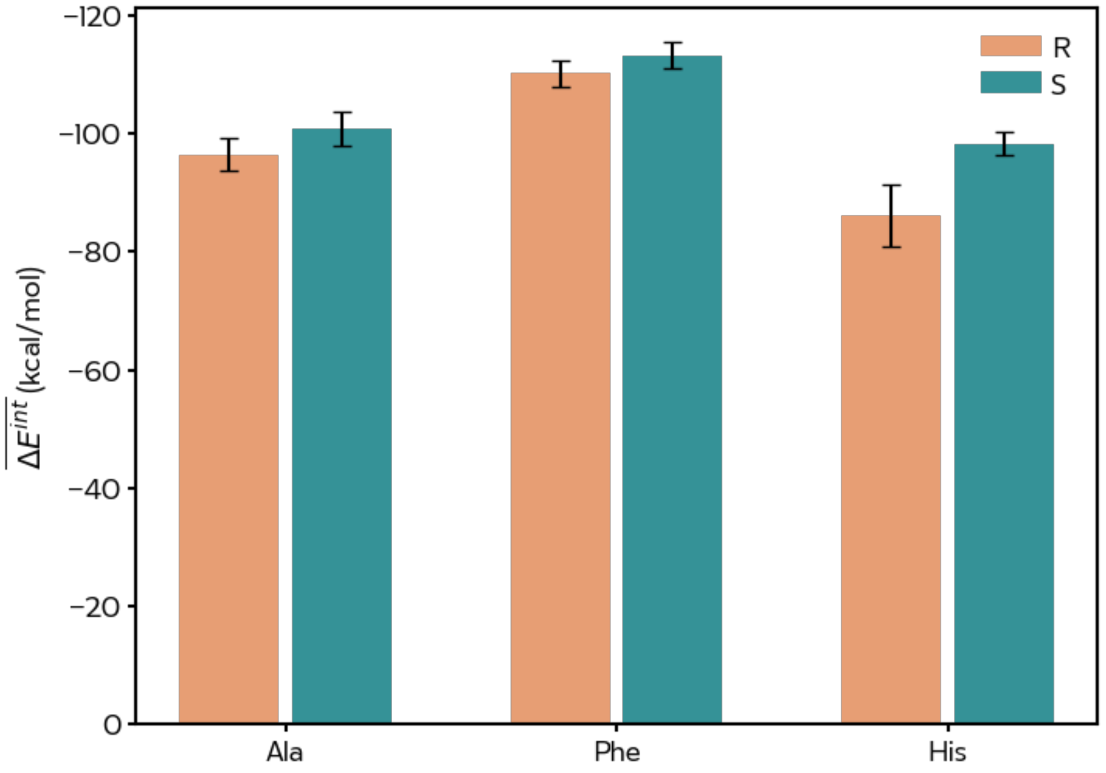

Figure S43. Average interaction energy ($\overline{\Delta \mathrm{E}^{\mathrm{int}}}$) and standard deviation for cetirizine enantiomers and the modified MOFs, considering the 45 lowest-energy configurations for each system.

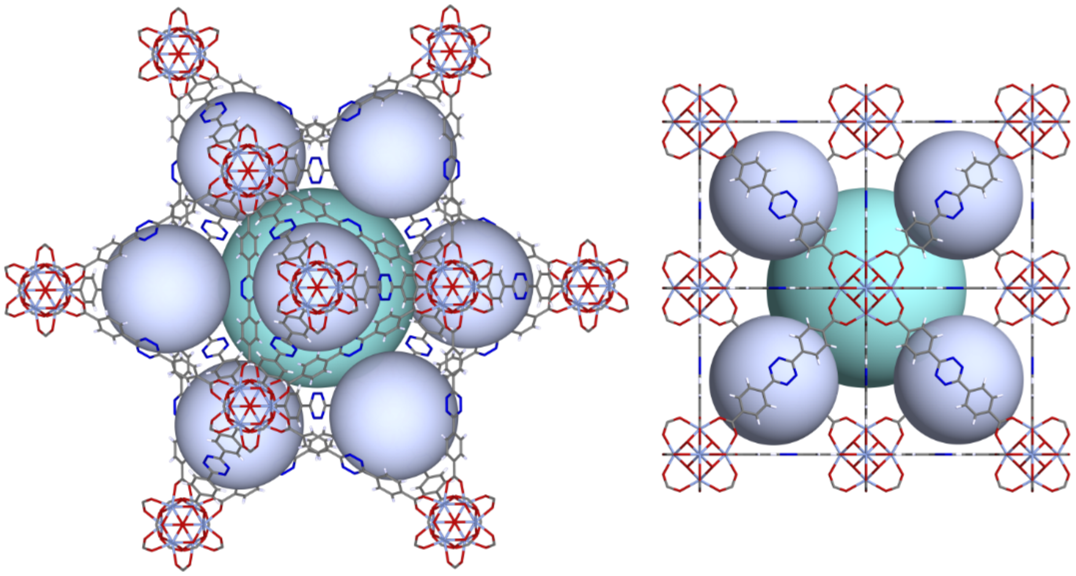

Figure S44. UiO-68-TZDC porosity represented by spheres in the tetrahedral (light purple) and octahedral (light blue) cages, shown in the [111] (left) and [100] (right) views. Color code: O in red, C in gray, H in white, N in blue, Zr in lavender color.

Table S11. Representative examples of enantioselective drug separation in MOFs studied computationally.

Technique	Drug	MOF	Energy (kcal·mol^{-1})	Ref.
energy-minimized with DFT (clusters) and molecular dynamics (diffusion coefficients (*D*)	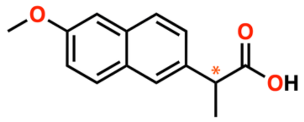 Naproxen	MOF-808-Ala	4.2	10
		MOF-808-Thr	2.2	
		MOF-808-His	14.1	
energy-minimized with DFT	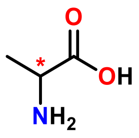 Alanine	CD-PlasmaCOF-1	5.6	24
energy-minimized with DFT	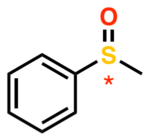 Methyl phenyl sulfoxide	[Zn_2(bdc){(*S*)-lac}(DMF)]	8.4	25
ab initio quantum calculations	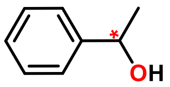 1-Phenylethanol	[Zn_2(bdc)(*S*-lac)(DMF)]	4.5	26
Monte Carlo simulations and discrete models of the MOF and quantum-chemistry calculations	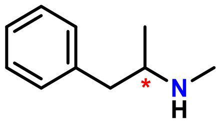 Methamphetamine	Cu(GHG)	3.62	9
	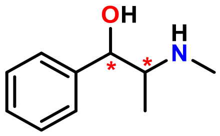 Ephedrine		12.48	

Technique	Drug	MOF	Energy (kcal·mol^{-1})	Ref.
hybrid Monte Carlo and hybrid molecular dynamics moves	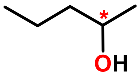 2-Pentanol	HMOF 1	-0.37	27
		HMOF 2	0.72	
	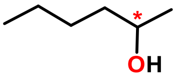 2-Hexanol	HMOF 1	-0.46	
		HMOF 2	0.31	
	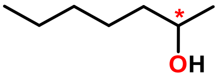 2-Heptanol	HMOF 1	-0.60	
		HMOF 2	-0.61	
	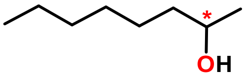 2-Octanol	HMOF 1	-0.62	
		HMOF 2	-0.06	
	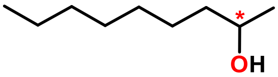 2-Nonanol	HMOF 1	-0.67	
		HMOF 2	0.002	
	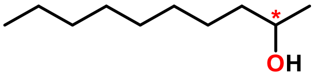 2-Decanol	HMOF 1	-0.67	
		HMOF 2	-0.59	
Monte Carlo simulations	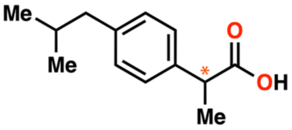 Ibuprofen	TAMOF-1	6.5	11
	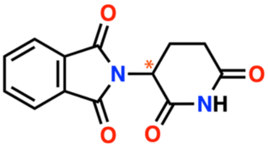 Thalidomide		0.40	

Technique	Drug	MOF	Energy (kcal·mol^{-1})	Ref.
energy-minimized with the solvent model density and DFT	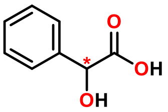<br>Mandelic acid	UiO-68-type Zr-HMOFs 1	0.47	28
		UiO-68-type Zr-HMOFs 3	0.88	
	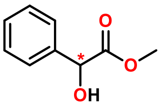<br>Methyl mandelate	UiO-68-type Zr-HMOFs 1	3.35	
		UiO-68-type Zr-HMOFs 3	5.66	
energy-minimized with the solvent model density and DFT	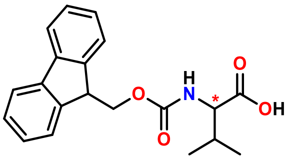<br>Fmoc-*L*/*D*-Val	UiO-68-HMOF-1	1.67	29
	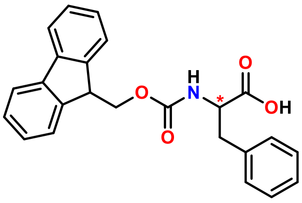<br>Fmoc-*L*/*D*-Phe	UiO-68-HMOF-3	0.43	
	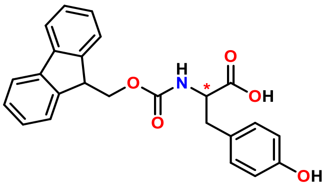<br>Fmoc-*L*/*D*-Try		2.69	
energy-minimized with DFT using ONIOM cluster models	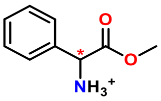<br>Protonated Phg-OMe	(*S*)-CE-1	2.9	13
simulation annealing and grand canonical Monte Carlo simulation	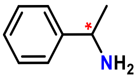<br>1-Phenylethylamine	$Mn_2L(DMF)_2(H_2O)_2$	0.66	30

S.7.3. ANALYSIS OF HOST-GUEST INTERACTIONS

To gain insight into the molecular origins of the experimentally observed stereoselectivity, a systematic analysis was performed on the 45 most stable configurations of each host–guest pair. The average number of peptidic moieties in proximity to the drug (**Figure S45**) was calculated using two distance cutoffs, 2.2 and 3.2 Å, corresponding to ranges where moderate and weak hydrogen bonds (HB) can, in principle, be formed.[31]

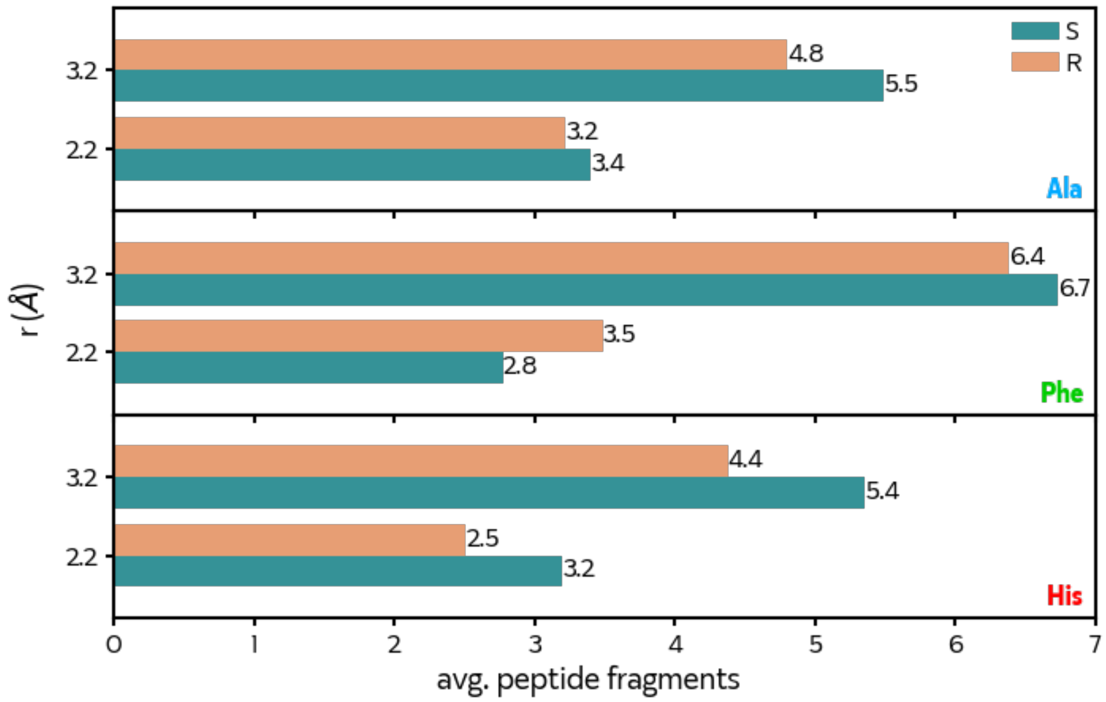

Figure S45. Average number of peptide fragments surrounding the drug at different cutoff distances (r).

Hydrogen bonds and π–π interactions were also identified based solely on geometrical criteria. The former interactions (**Figure S46a**) were classified as strong or moderate according to geometric criteria: strong HBs correspond to H···acceptor distances up to 1.5 Å and donor-H···acceptor angles of 165-180°, whereas moderate HBs correspond to distances up to 2.2 Å and angles of 130-180°.[31] Predominantly moderate HBs were observed across the studied systems. **Table S12** summarizes the number of HBs for each host-guest system, reported both for the most stable conformation (N = 1) and as averages over the 45 most stable geometries.

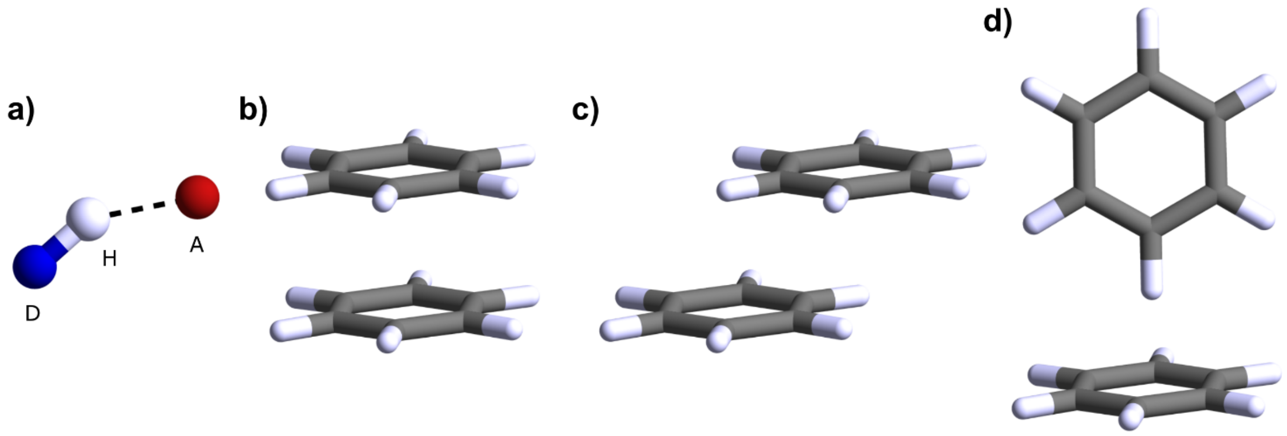

Figure S46. Types of interactions analyzed using geometrical criteria. a) Hydrogen bond (dashed line), with HB donor (D) and acceptor (A) atoms indicated. b) Parallel-stacked, c) parallel-displaced and d) T-shaped π-π interactions.

Table S12. Average interaction energies ($\overline{\Delta E^{int}}/kcal\,mol^{-}$) calculated from the **N** most stable geometries, and relative interaction energies ($\Delta\overline{\Delta E^{int}}/kcal\,mol^{-}$) between the *S* and *R* stereoisomers of CTZ. Number of moderate hydrogen bonds between the drug and the MOF (**HB_{DM}**), classified by whether the drug acts as HB donor or acceptor. Intramolecular hydrogen bonds within the drug (**HB_{DD}**) and within the MOF (**HB_{MM}**).

AA	CTZ	N	$\overline{\Delta E^{int}}$	$\Delta\overline{\Delta E^{int}}$	HB_{DM}	HB_{DM} (D donor)	HB_{DM} (D acceptor)	HB_{DD}	HB_{MM}
**Ala**	*S*	1	-107.5	-5.5	6.0	5.0	1.0	-	24.0
	R	1	-102.0		9.0	4.0	5.0	-	14.0
	S	45	-100.8	-4.3	5.7	3.0	2.8	0.1	20.7
	R	45	-96.5		6.7	3.5	3.2	0.4	15.7
**Phe**	*S*	1	-118.2	-2.7	5.0	2.0	3.0	-	28.0
	R	1	-115.5		11.0	6.0	5.0	-	35.0
	S	45	-113.2	-3.0	6.0	3.7	2.3	-	26.7
	R	45	-110.2		6.9	3.4	3.5	-	29.6
**His**	*S*	1	-104.1	-9.0	4.0	3.0	1.0	-	21.0
	R	1	-95.1		7.0	3.0	4.0	-	24.0
	S	45	-98.2	-12.1	5.1	2.6	2.4	0.1	21.4
	R	45	-86.1		4.9	2.0	2.9	0.6	21.7

The π–π interactions were identified and classified according to the criteria reported by Zhao and co-workers.[32] In addition to the interaction types illustrated by **Figure S46b-d**, intermediate conformations were also defined as geometries between T-shaped and parallel arrangements, with acute angles between the normal vectors of the interacting planes ranging from 30º to 50º. The systematic analysis considered both 5- and 6-membered aromatic rings. The average number of each type of π–π interaction in the drug–MOF systems are reported in **Table S13**, considering both the most stable configuration and the 45 lowest-energy geometries. For the studied systems, T-shaped π–π interactions are the most prevalent, typically occurring as intramolecular interactions within the MOF.

Table S13. Average number of π-π interactions in the **N** most stable geometries, classified by the type of interaction (**Figure S46b-d**) and species involved. Average T-shaped (**T**), intermediate (**I**, between T-shaped and parallel), parallel-displaced (**Pd**) and parallel-stacked (**Ps**) interactions between drug and MOF (**DM**) or within the MOF (**MM**).

AA	CTZ	N	T_{DM}	T_{MM}	I_{DM}	I_{MM}	Pd_{DM}	Pd_{MM}	Ps_{DM}	Ps_{MM}
**Ala**	*S*	1	-	9.0	1.0	2.0	-	-	-	-
	R	1	-	9.0	1.0	-	-	-	-	-
	S	45	0.4	7.6	0.7	1.5	0.1	0.4	-	-
	R	45	0.2	6.4	0.3	1.7	0.6	0.1	0.2	-
**Phe**	*S*	1	-	13.0	-	4.0	-	-	-	-
	R	1	1.0	9.0	-	3.0	-	2.0	-	-
	S	45	0.3	14.7	-	2.7	-	1.8	-	-
	R	45	1.0	9.3	0.2	3.8	-	2.5	-	0.1
**His**	*S*	1	-	14.0	-	1.0	-	1.0	-	-
	R	1	-	13.0	-	-	-	-	-	-
	S	45	1.3	12.1	0.2	1.5	-	0.6	-	-
	R	45	0.8	8.0	0.2	1.9	0.2	0.8	-	0.1

Further analysis of drug-MOF interactions was performed using the noncovalent interaction (NCI) method[33] with the Multiwfn software.[34] Gradient isosurfaces for the whole unit cell were computed using the promolecular density approach, which in our previous work yielded results comparable to those obtained with DFT electron densities of MOF fragments.[34] The complete NCI plots for His···(*R*)/(*S*)-CTZ are shown in **Figure S47a,b**, while **Figures S47c,d** display only the isosurfaces surrounding the drug for clearer visualization. Corresponding 3D views are provided in **Movies S2** and **S1**. Green regions are predominantly observed around the drug, corresponding to weak van der Waals interactions, which are more pronounced in the *S* enantiomer. The pocket-like conformation promotes a richer array of dispersive interactions, driving the enantioselectivity of the His-containing framework toward the *S* form of the drug. Red ellipsoids within the rings indicate steric effects, whereas blue regions denote strong attractive interactions such as hydrogen bonds.

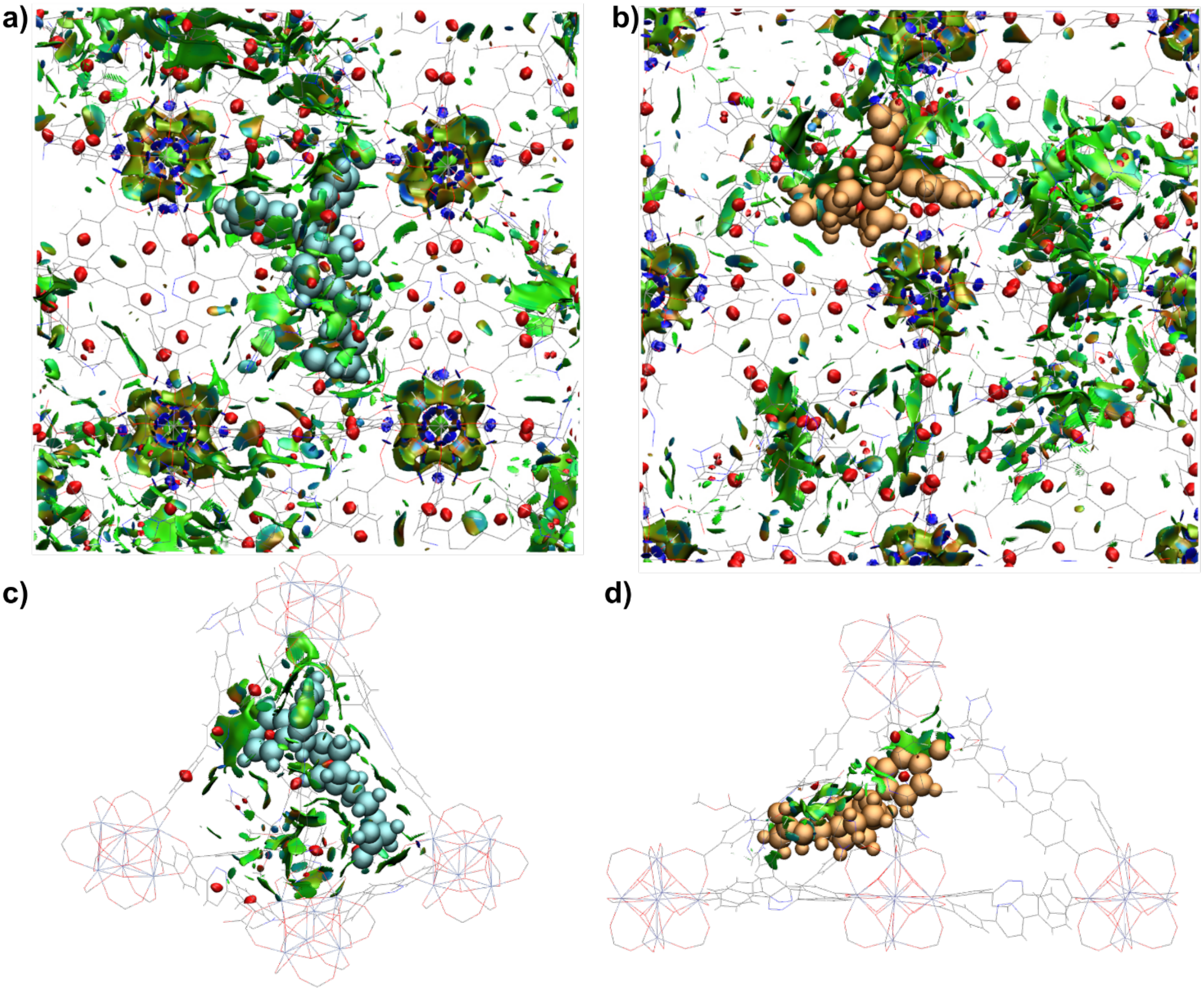

Figure S47. NCI plots of the most stable conformations of a) His···*S*-CTZ and b) His···*R*-CTZ (right), computed using the promolecular density approximation. Gradient isosurfaces were generated at an isovalue of 0.3. Red and blue regions correspond to strong repulsive and attractive interactions, respectively, while green indicates weak van der Waals interactions. c,d) Detailed view of noncovalent interactions around the drug in c) His···*S*-CTZ and d) His···*R*-CTZ systems, shown as representative frames from **Movies S2** and **S1**, respectively.

S.8. NMR SPECTRA

Compound 1

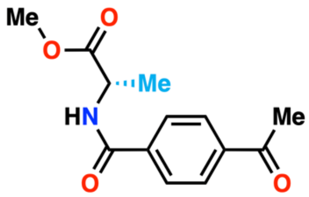

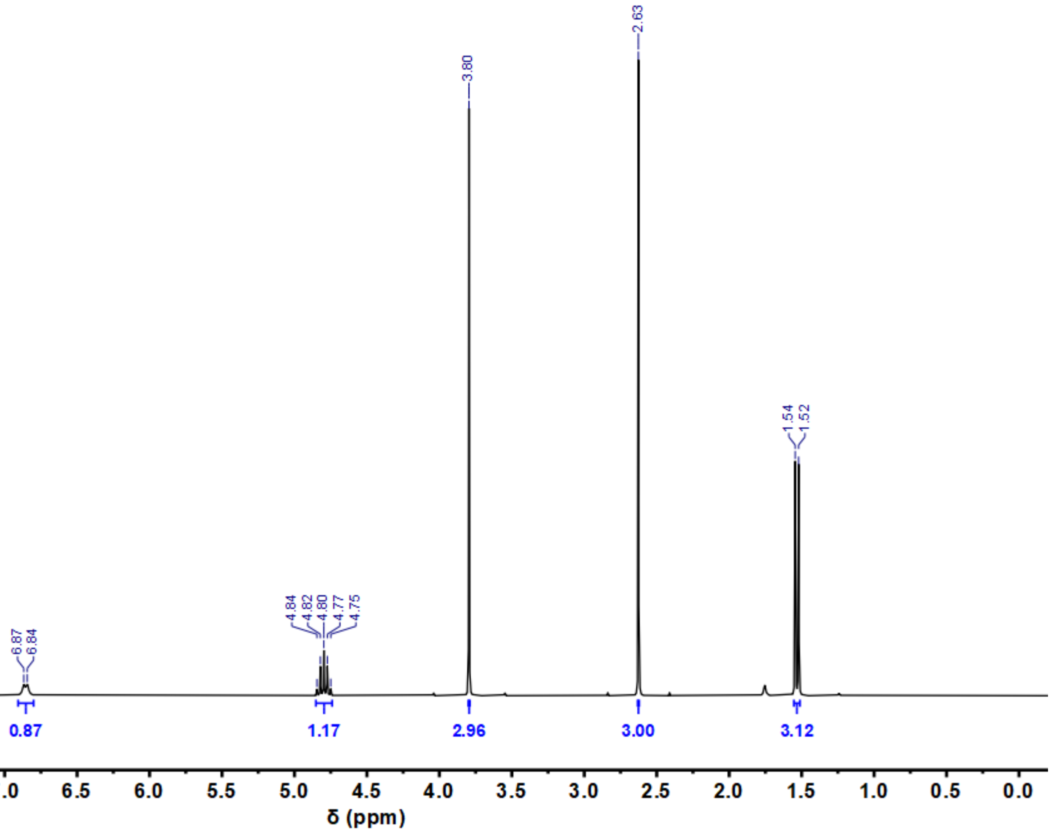

Figure S48. ^{1}H NMR spectrum of compound **1** in $CDCl_3$.

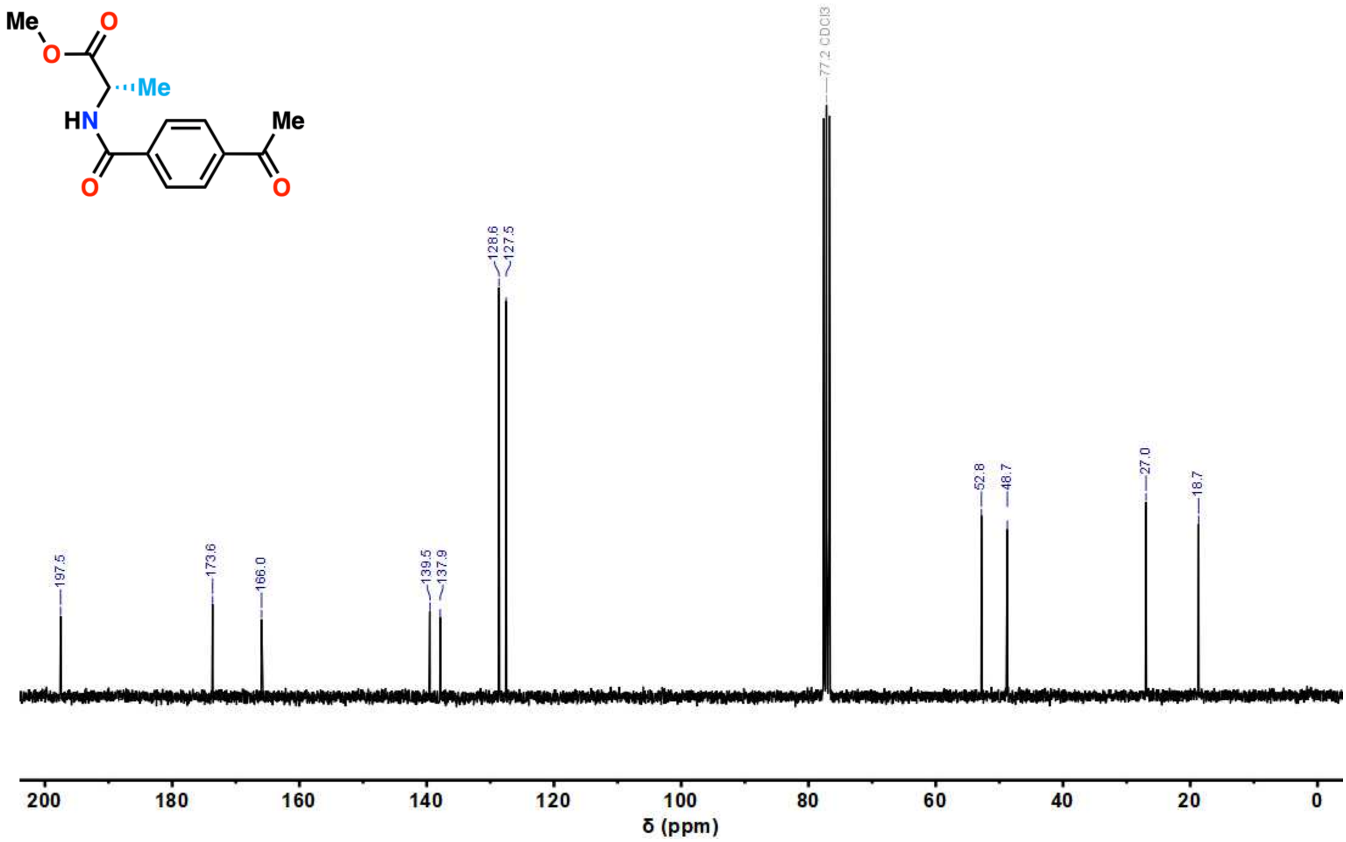

Figure S49. ^{13}C NMR spectrum of compound **1** in $CDCl_3$.

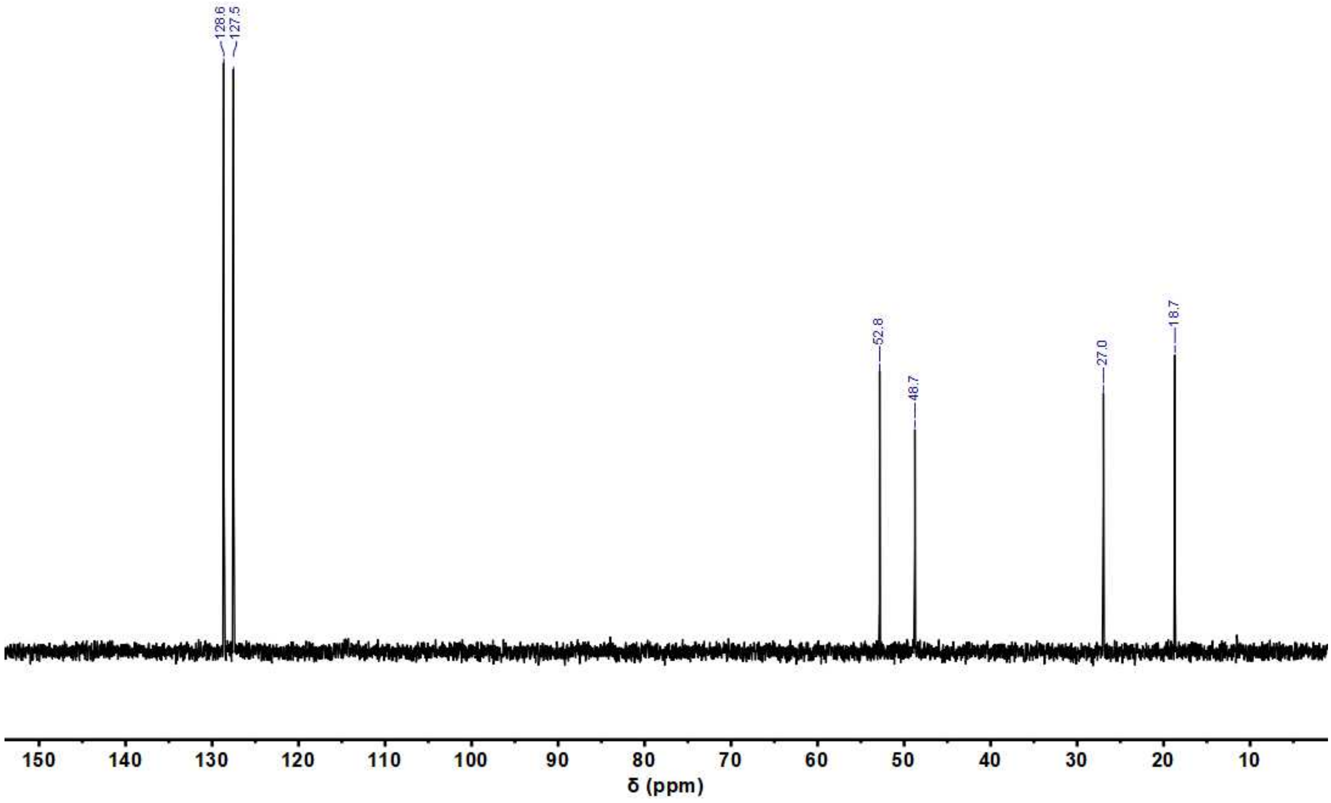

Figure S50. DEPT-135 spectrum of compound **1** in $CDCl_3$.

Compound 2

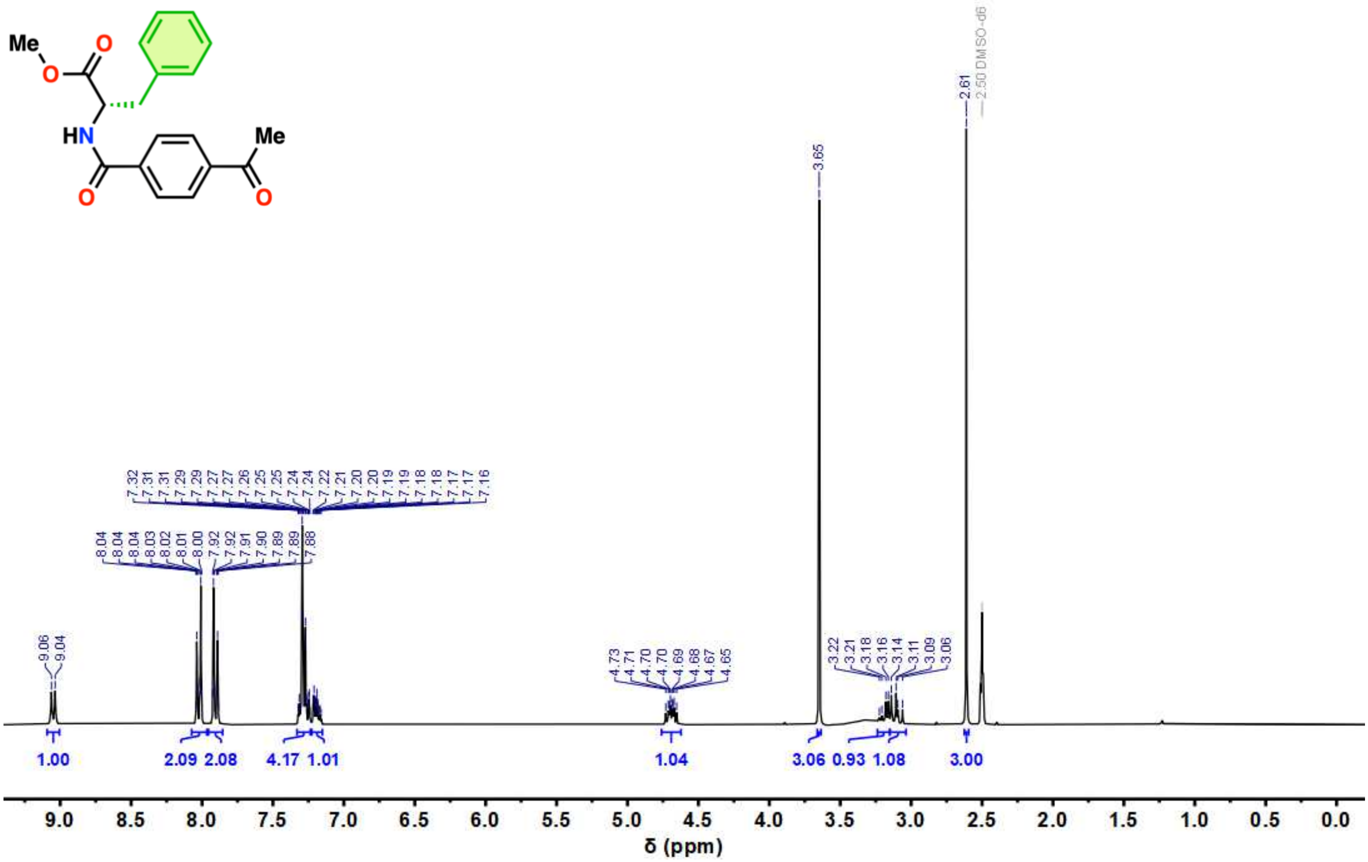

Figure S51. ^{1}H NMR spectrum of compound **2** in DMSO-d_6.

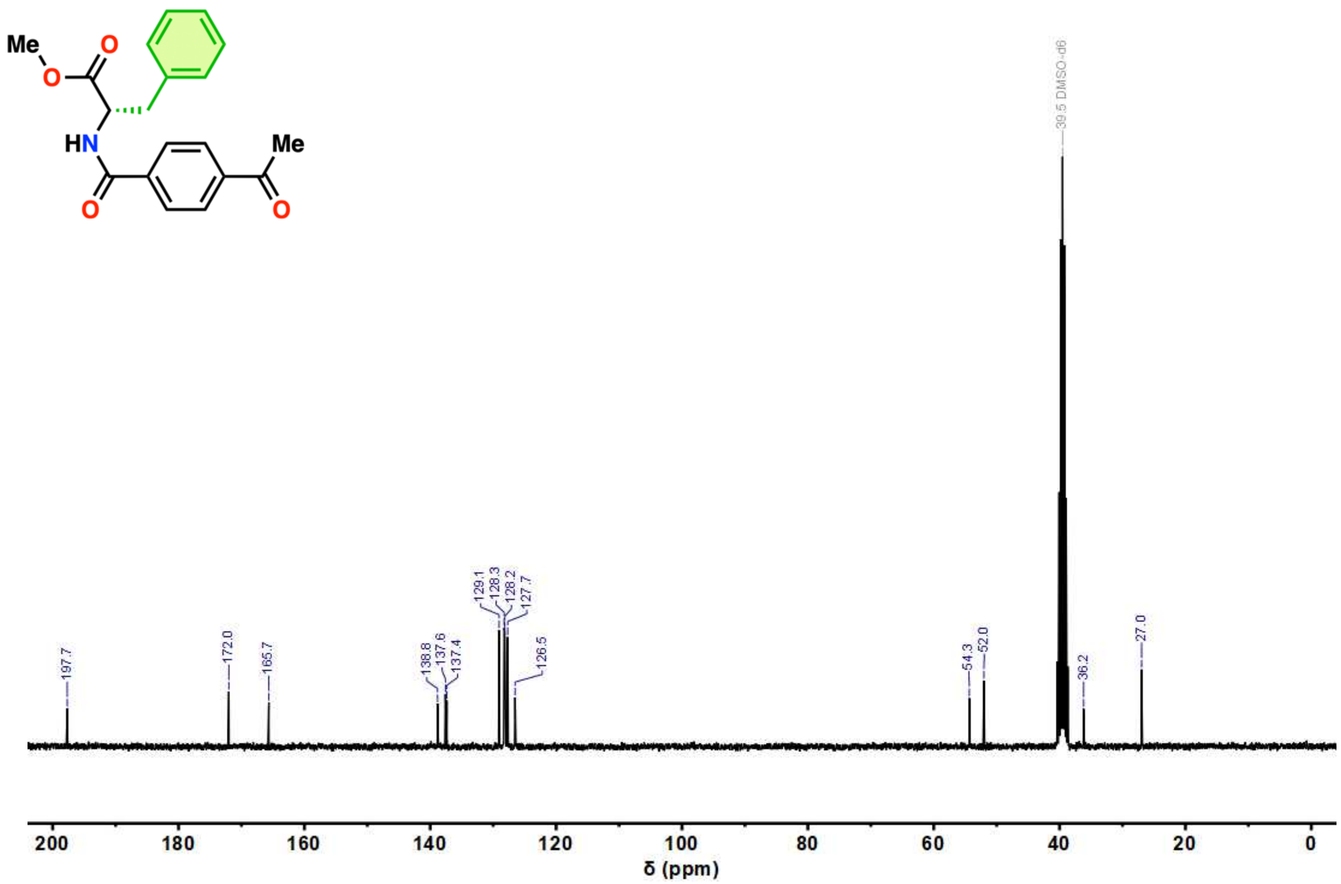

Figure S52. ^{13}C NMR spectrum of compound **2** in DMSO-d_6.

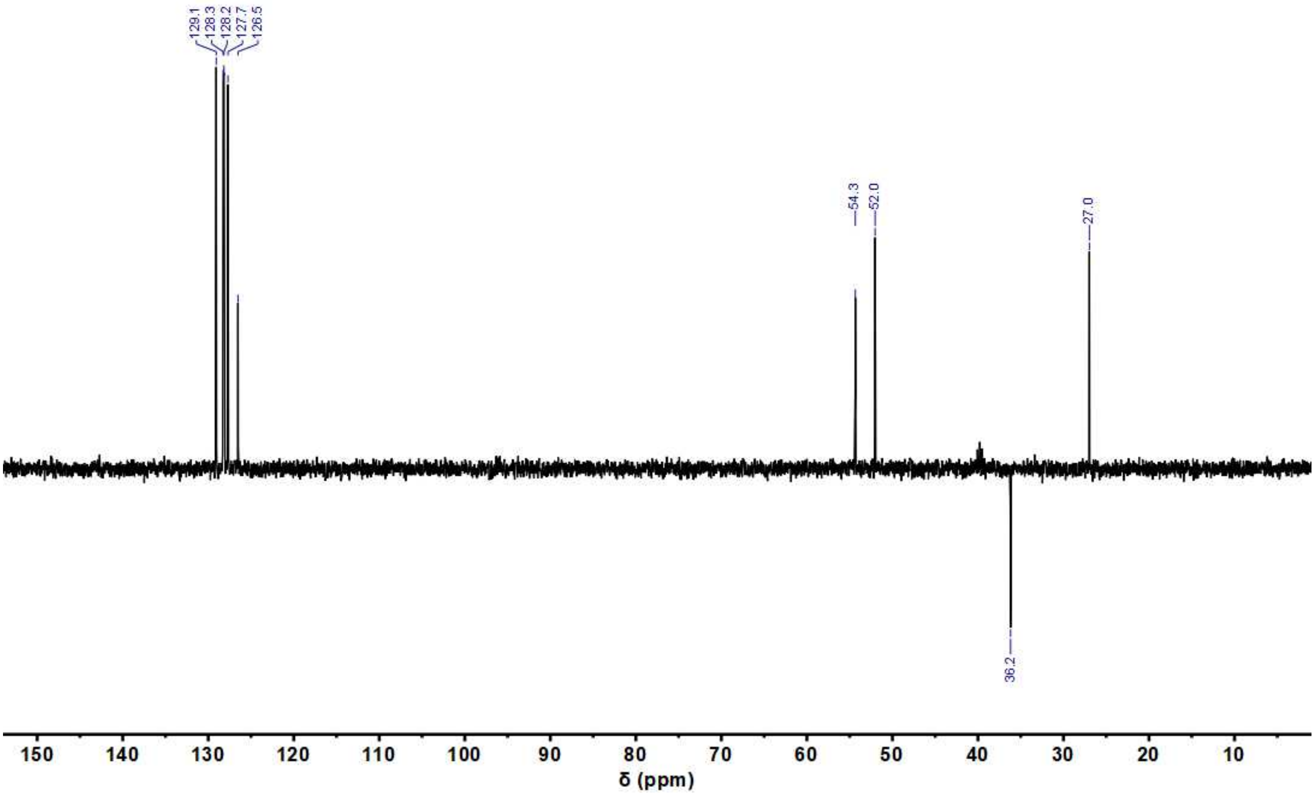

Figure S53. DEPT-135 spectrum of compound **2** in DMSO-d_6.

Compound 3

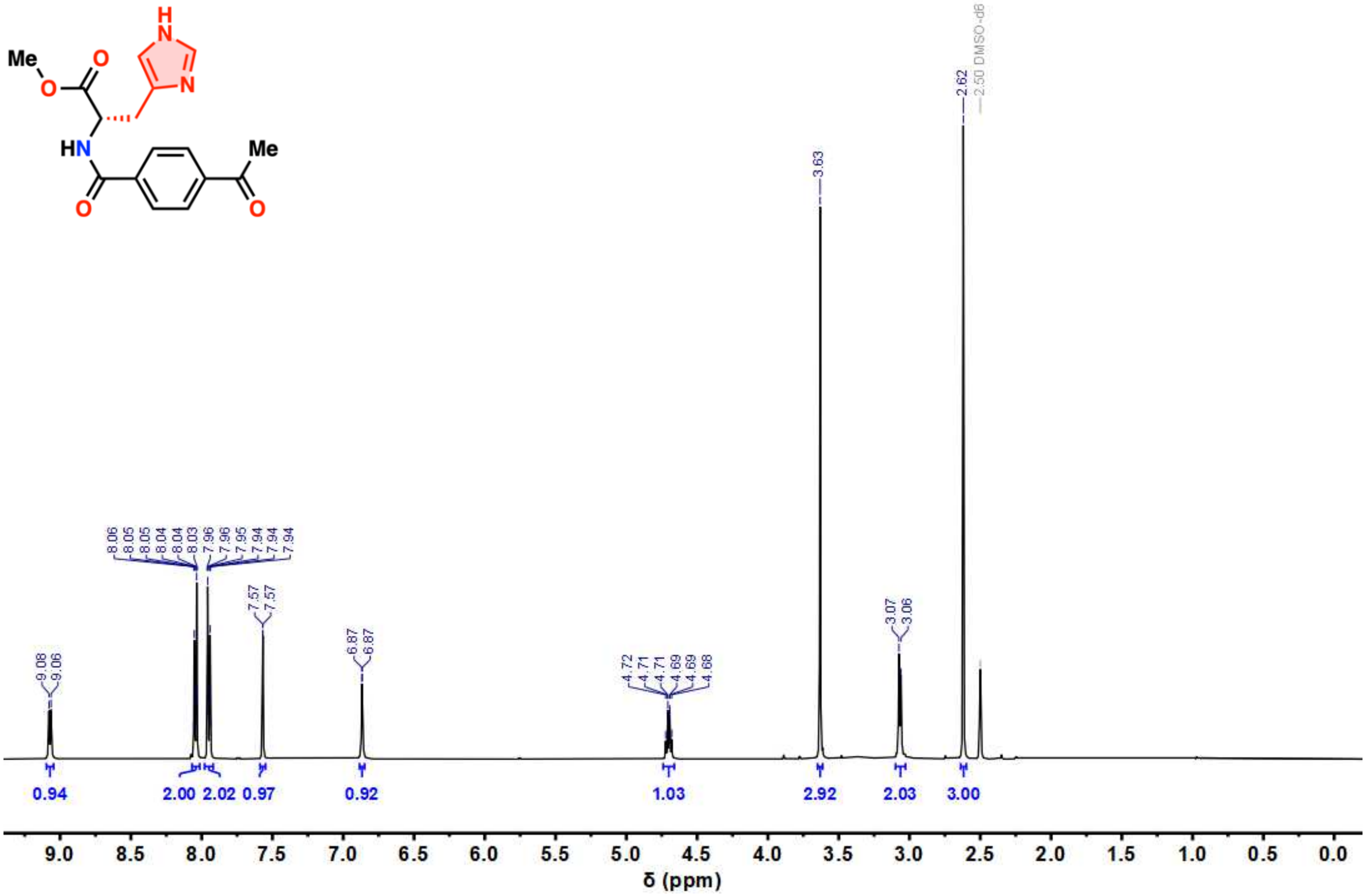

Figure S54. ^{1}H NMR spectrum of compound **3** in DMSO-d_6.

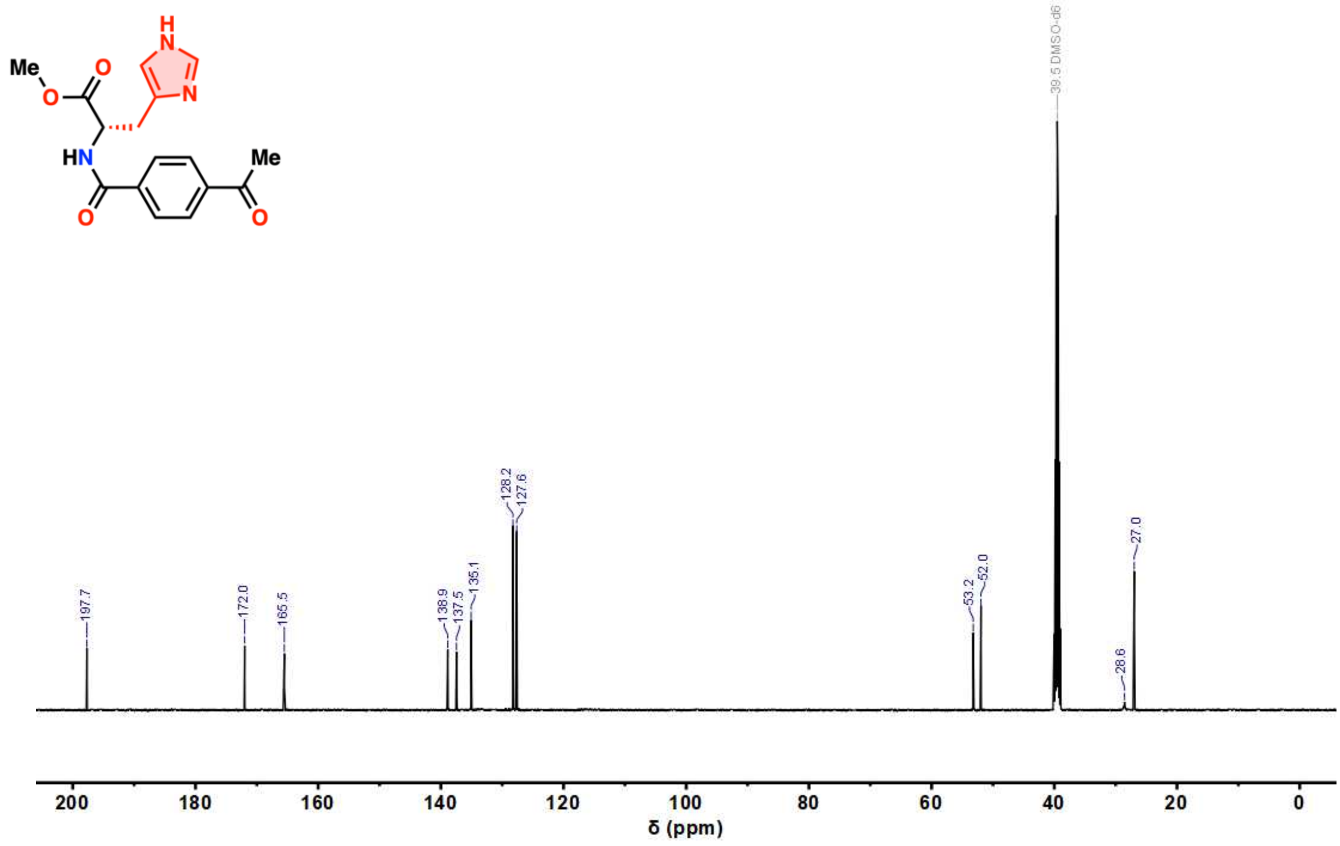

Figure S55. ^{13}C NMR spectrum of compound **3** in DMSO-d_6.

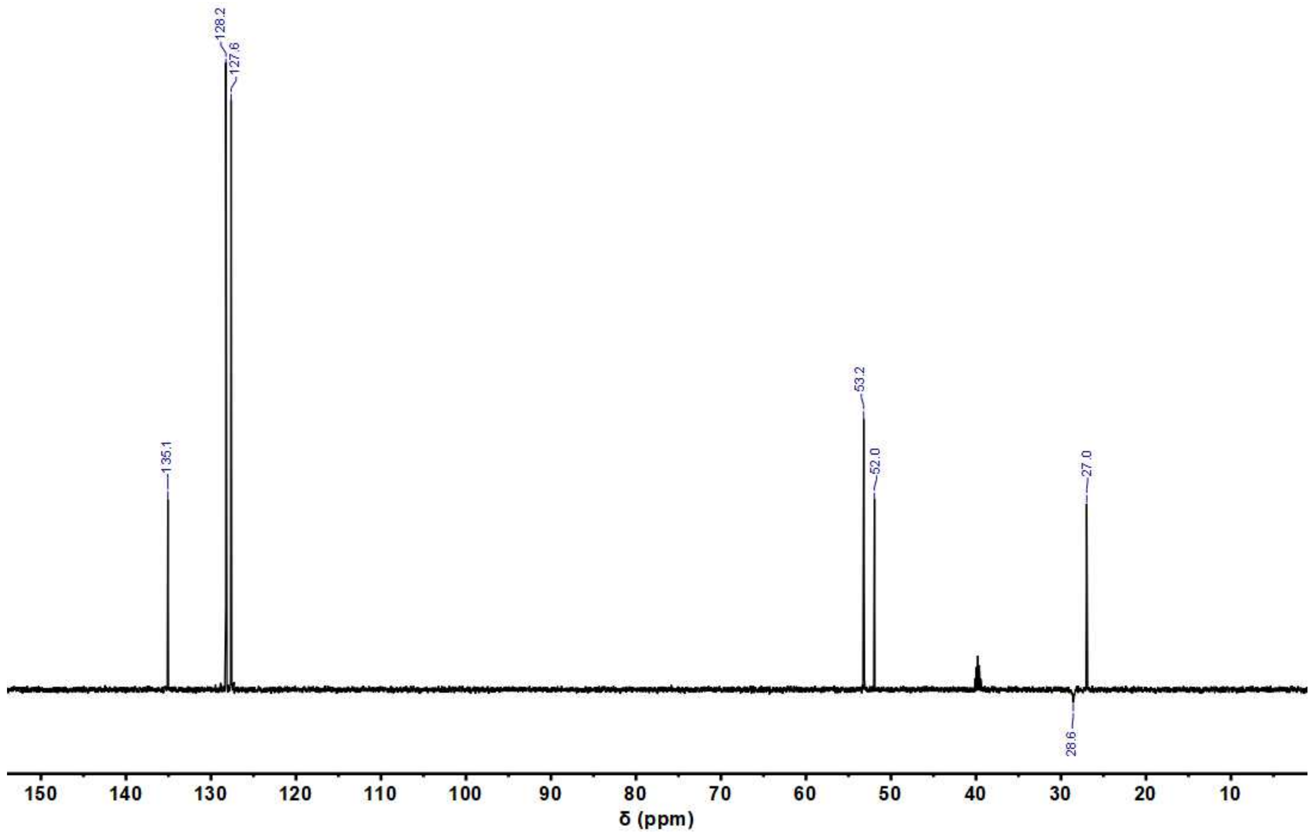

Figure S56. DEPT-135 spectrum of compound **3**.

Compound 4

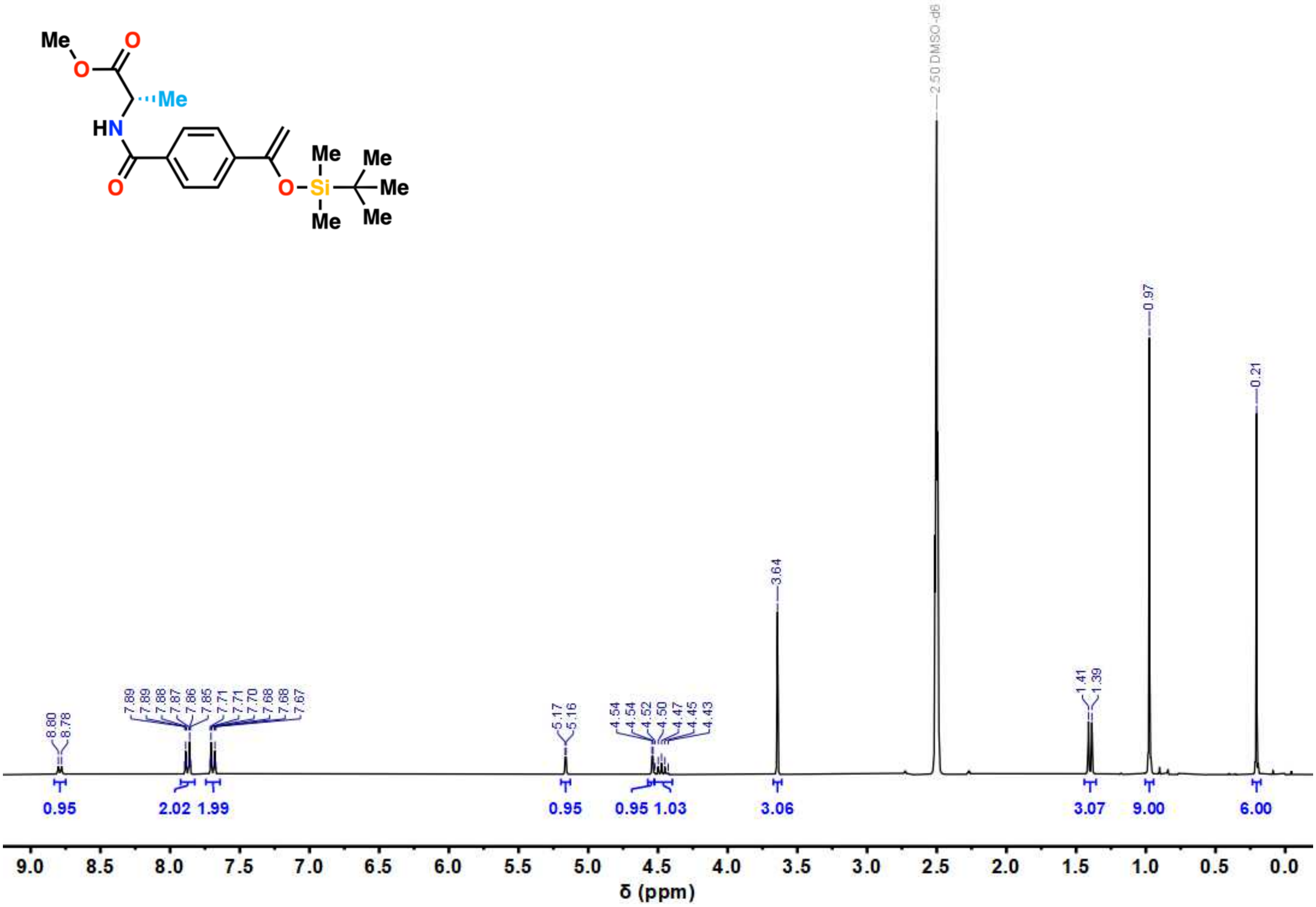

Figure S57. ^{1}H NMR spectrum of compound **4** in DMSO-d_6.

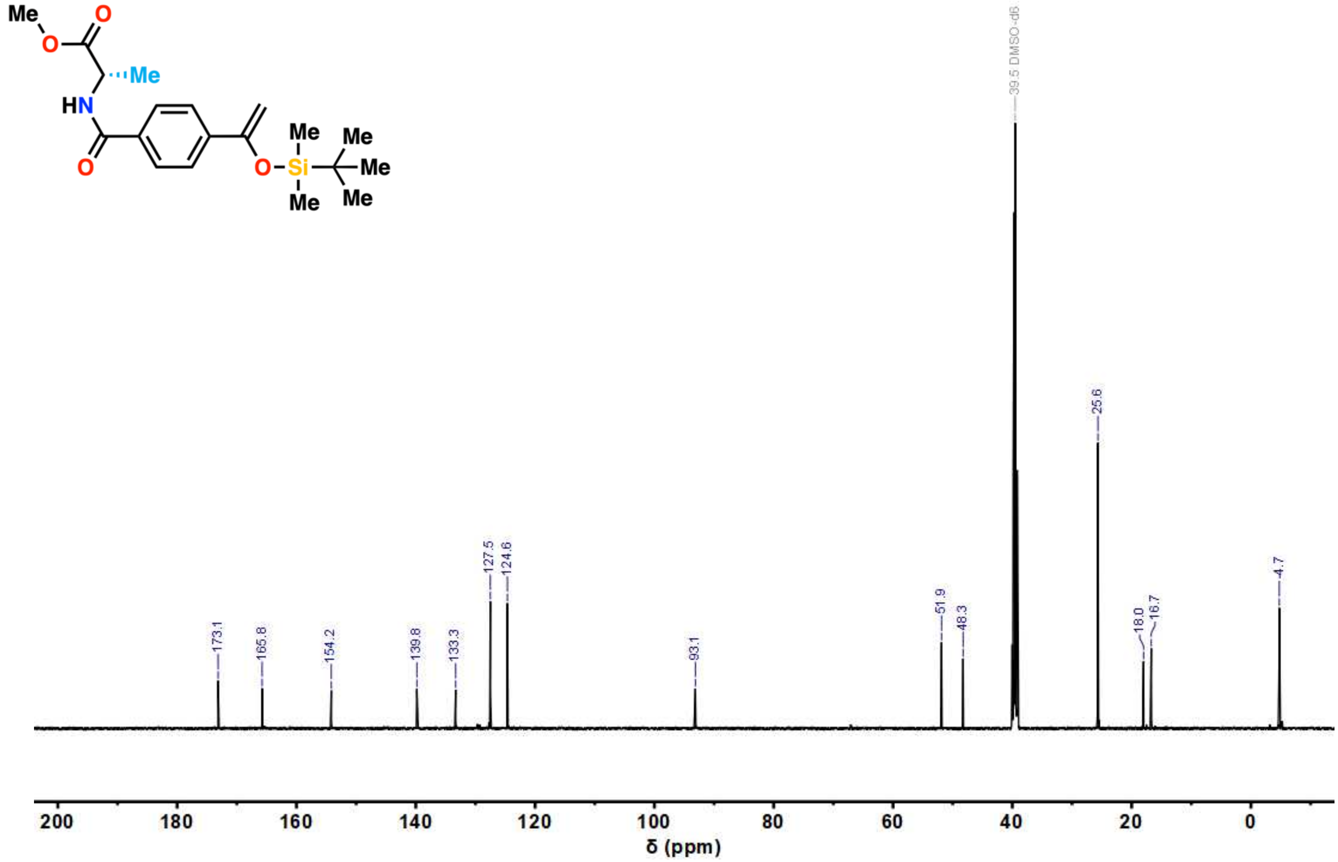

Figure S58. ^{13}C NMR spectrum of compound **4** in DMSO-d_6.

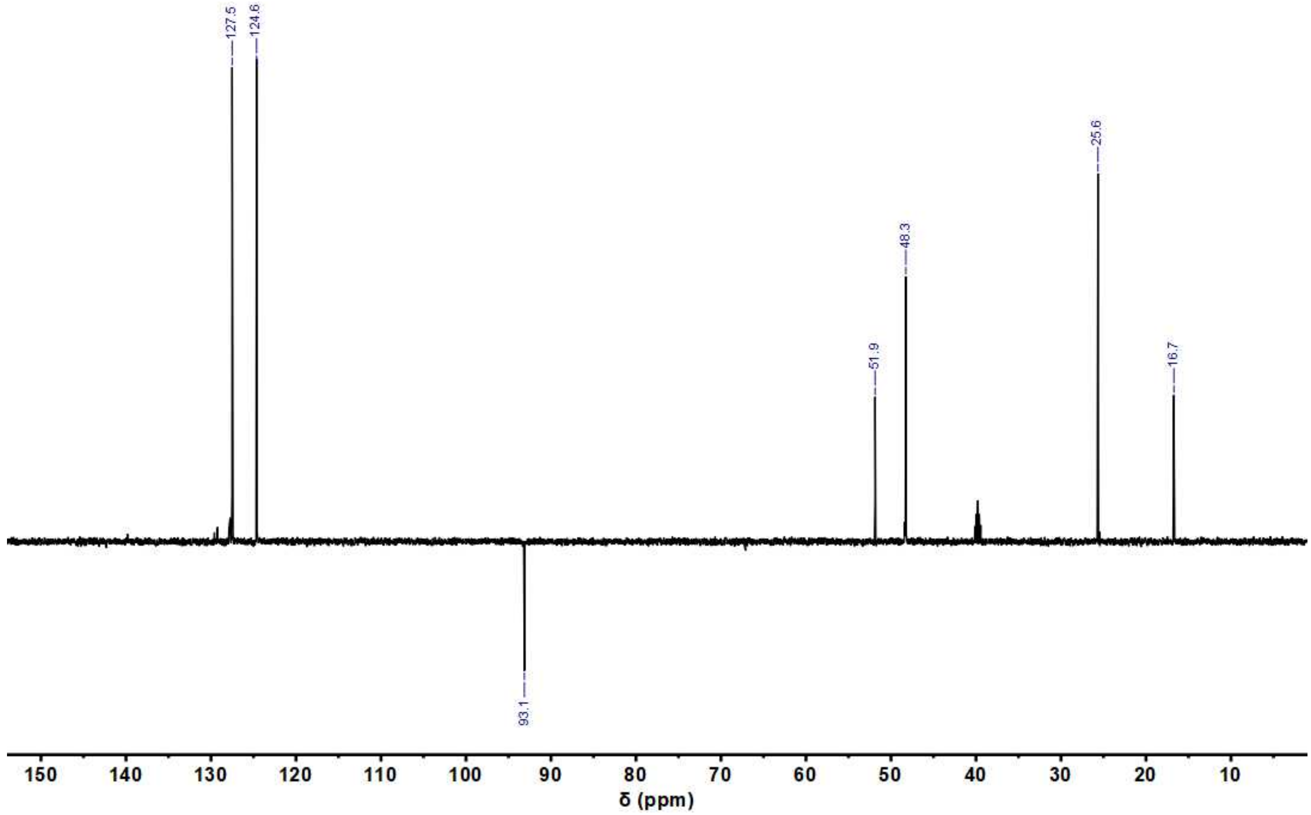

Figure S59. DEPT-135 spectrum of compound **4** in DMSO-d_6.

Compound 5

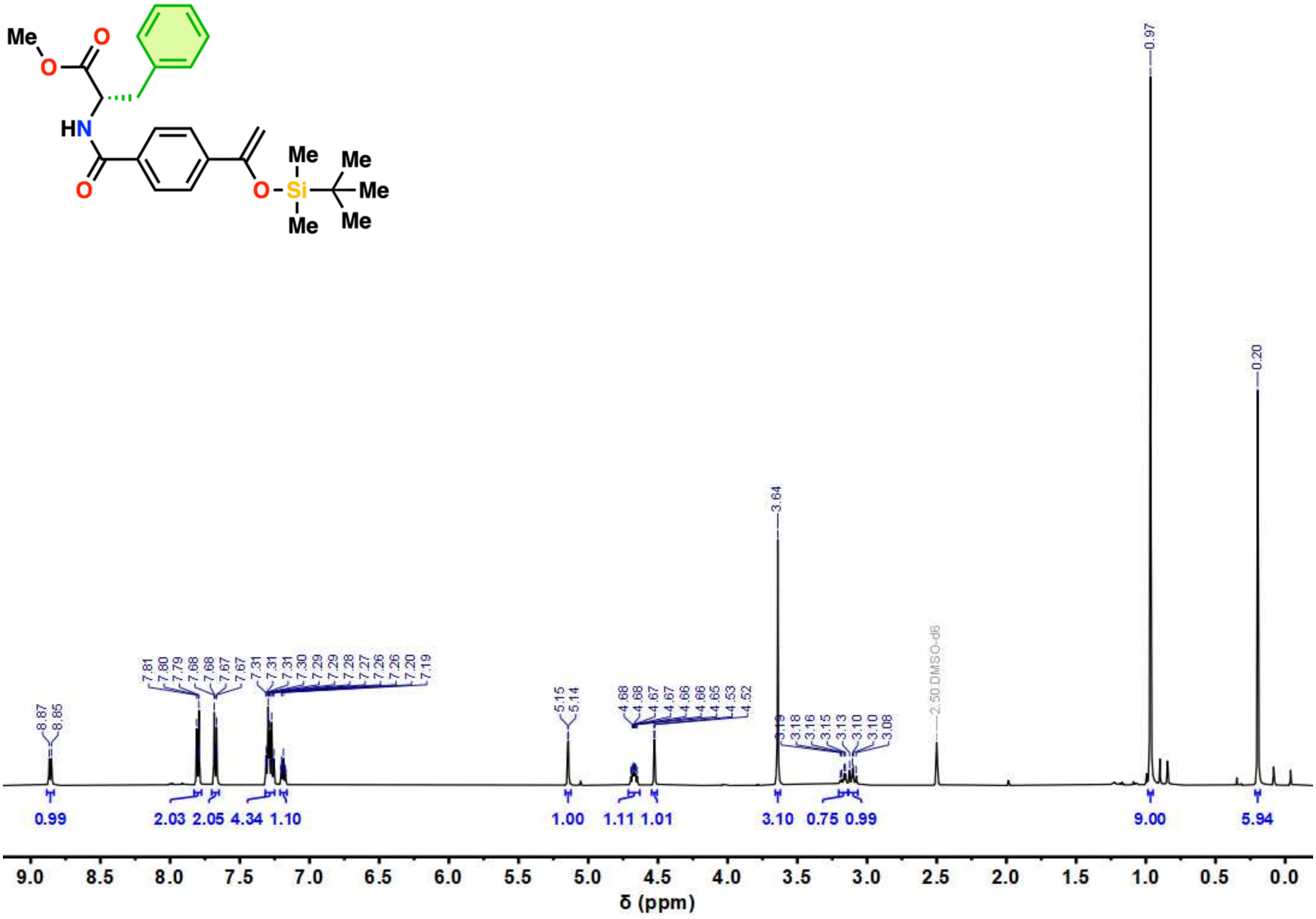

Figure S60. ^{1}H NMR spectrum of compound **5** in DMSO-d_6.

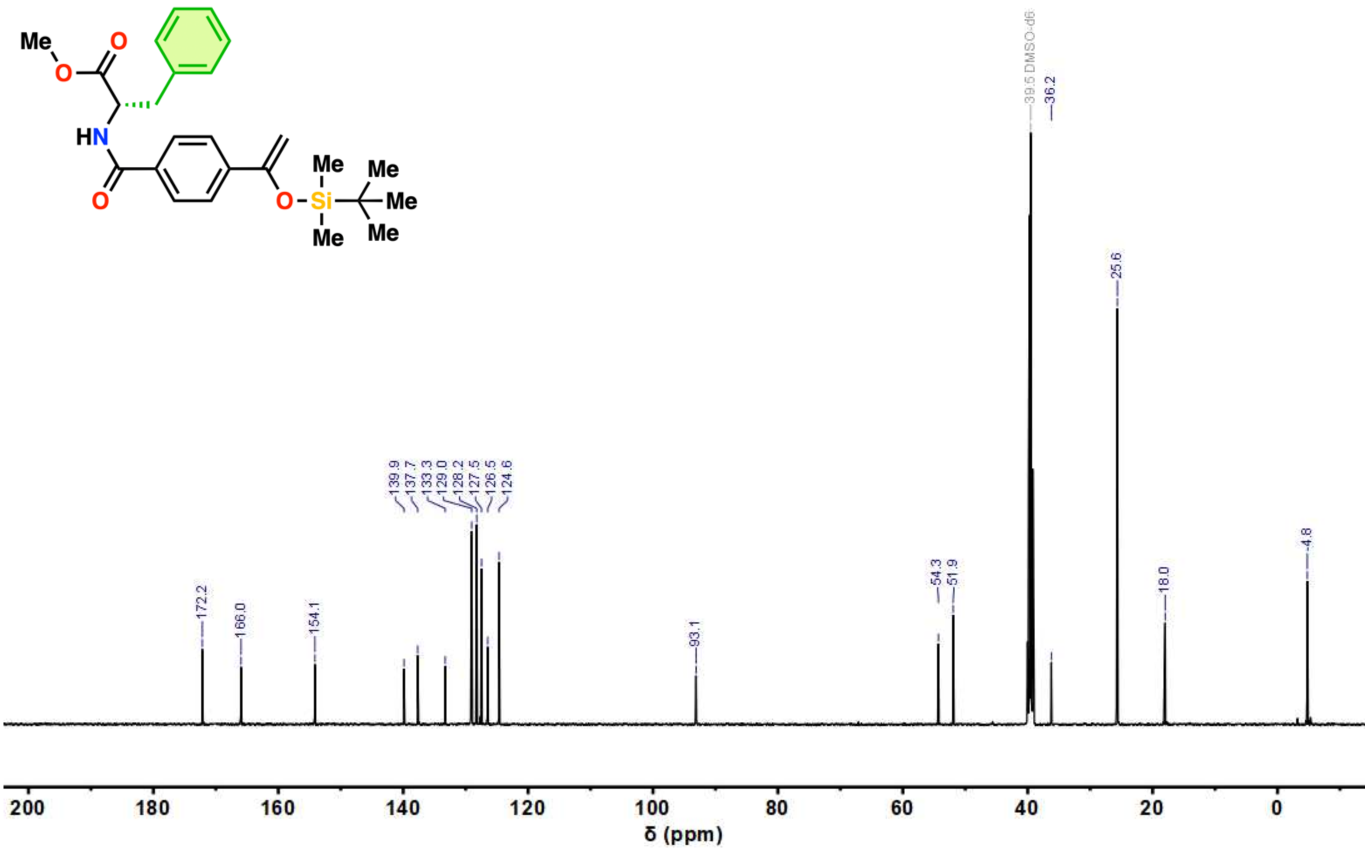

Figure S61. ^{13}C NMR spectrum of compound **5** in DMSO-d_6.

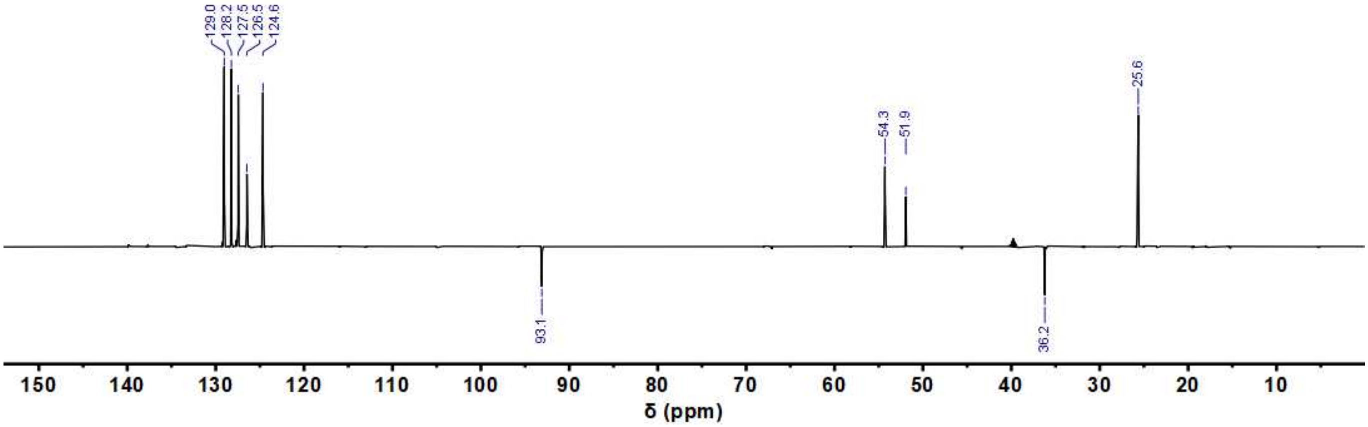

Figure S62. DEPT-135 spectrum of compound **5** in DMSO-d_6.

Compound 6

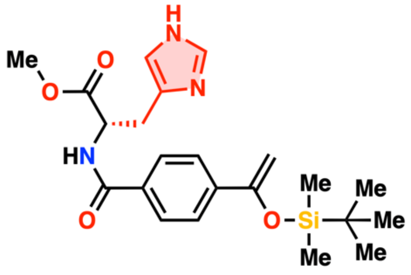

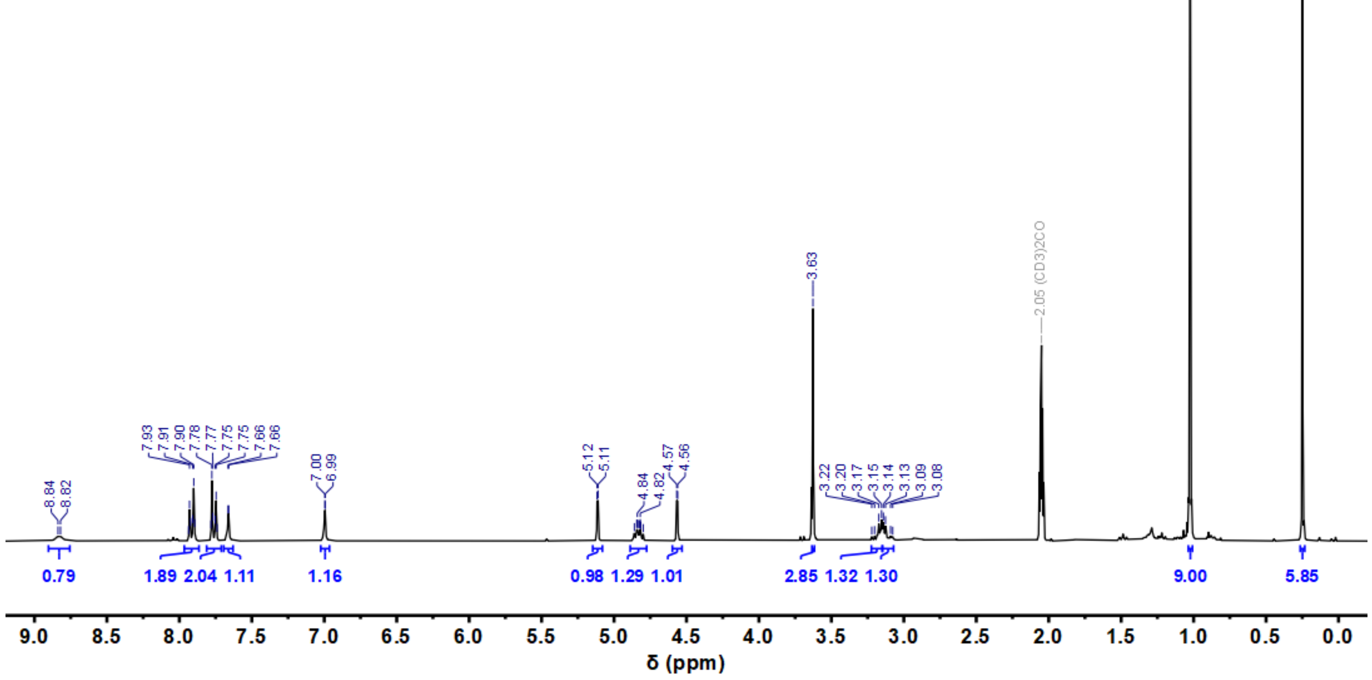

Figure S63. ^{1}H NMR spectrum of compound **6** in Acetone-d_3.

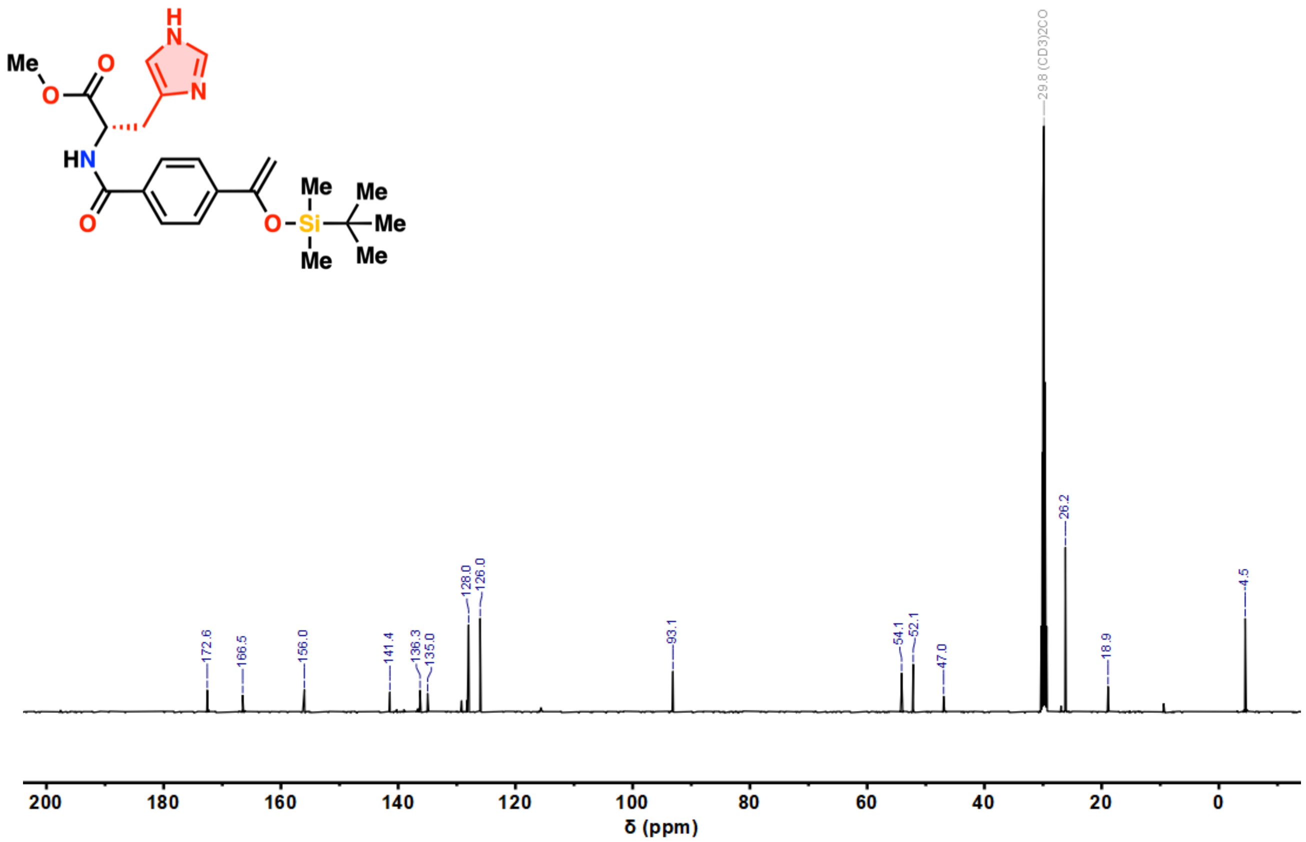

Figure S64. ^{13}C NMR spectrum of compound **6** Acetone-*d₃*.

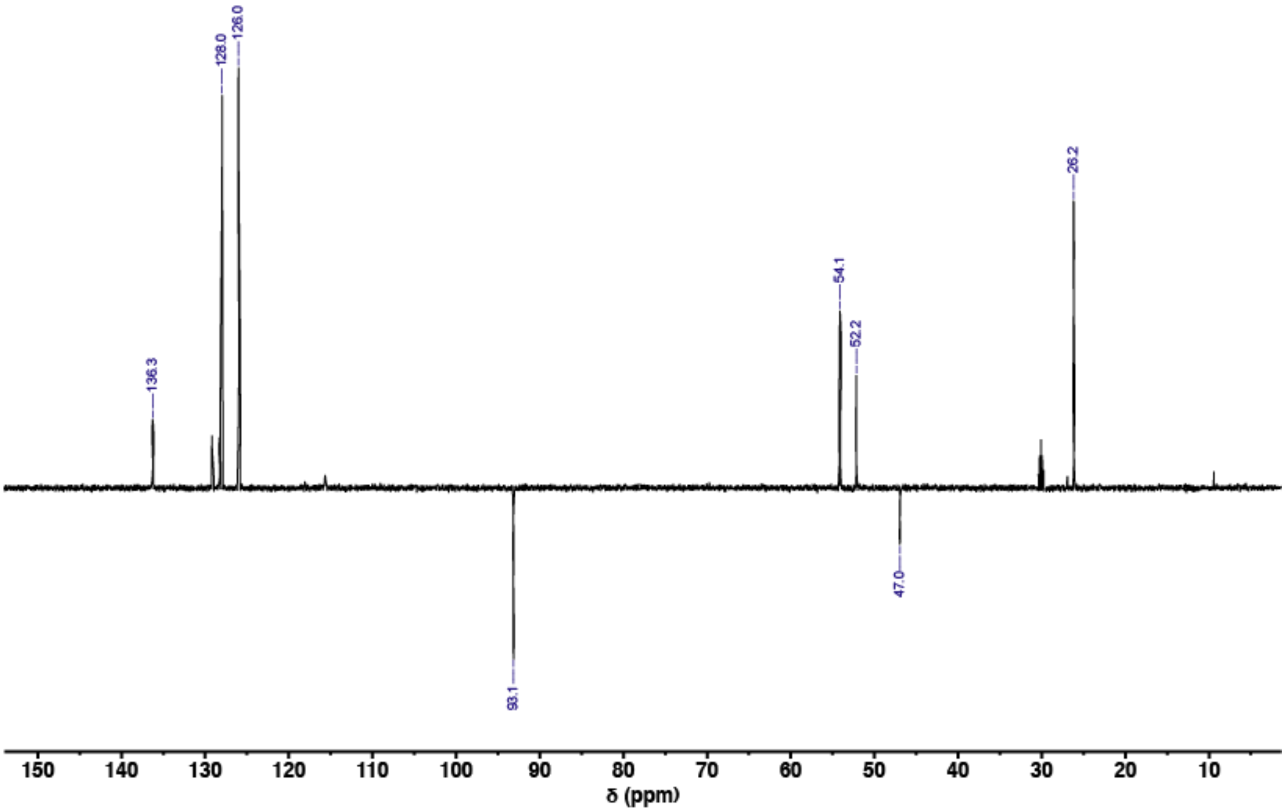

Figure S65. DEPT-135 spectrum of compound **6** Acetone-*d₃*.

Digested UiO-68-PZDC-(*L*)-Ala

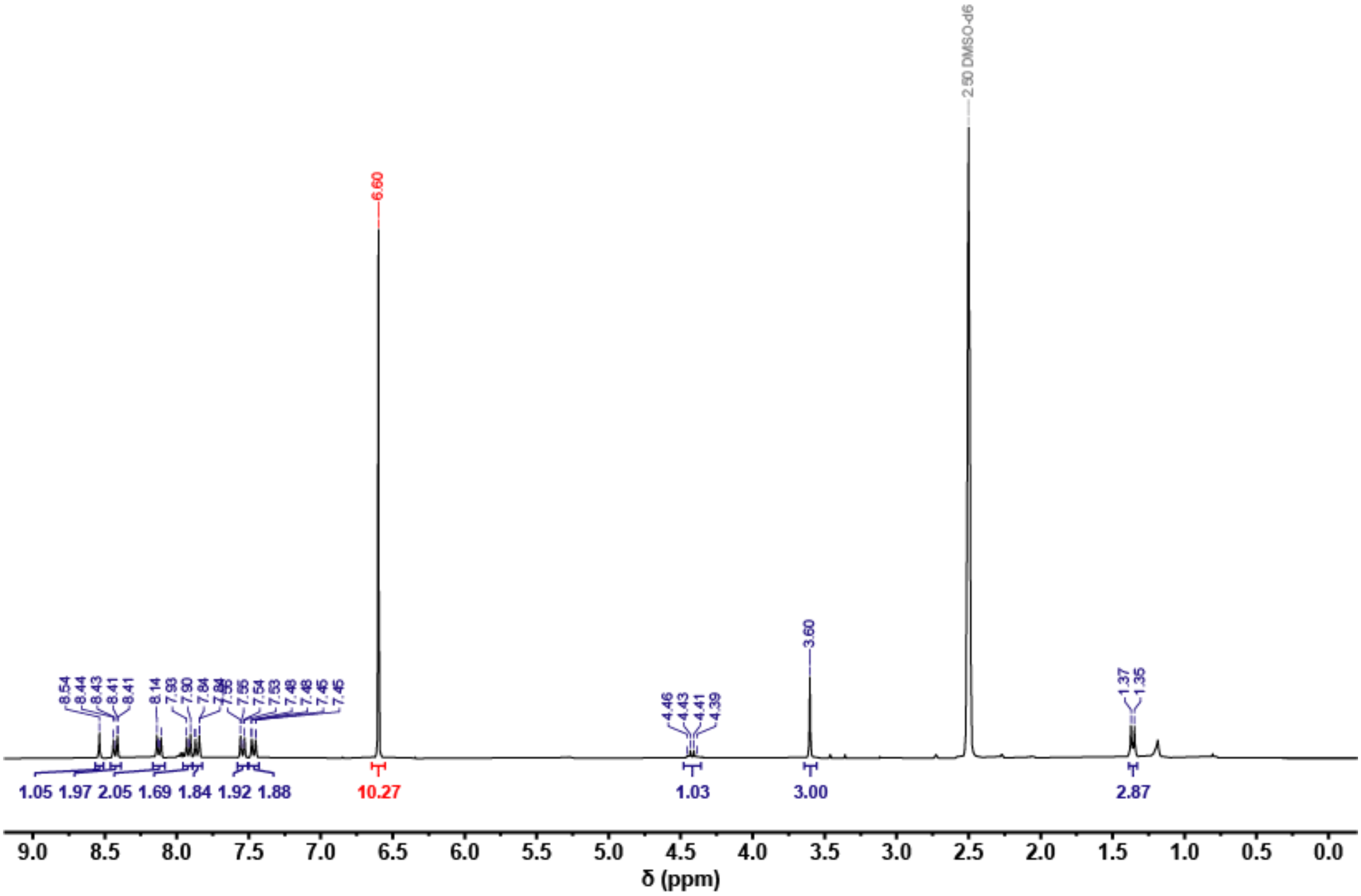

Figure S66. ^{1}H NMR quantification spectrum of UiO-68-PZDC-(*L*)-Ala. The signal in red corresponds to fumaric acid. Signals in blue correspond to the modified linker: ^{1}H NMR (300 MHz, DMSO): δ_H 8.54 (s, 1H), 8.47 – 8.38 (m, 2H), 8.15 – 8.10 (m, 2H), 7.94 – 7.90 (m, 2H), 7.90 – 7.81 (m, 2H), 7.57 – 7.52 (m, 2H), 7.50 – 7.44 (m, 2H), 4.42 (q, *J* = 7.1 Hz, 1H), 3.60 (s, 3H), 1.36 (d, *J* = 7.3 Hz, 3H).

Digested UiO-68-PZDC-(*L*)-Phe

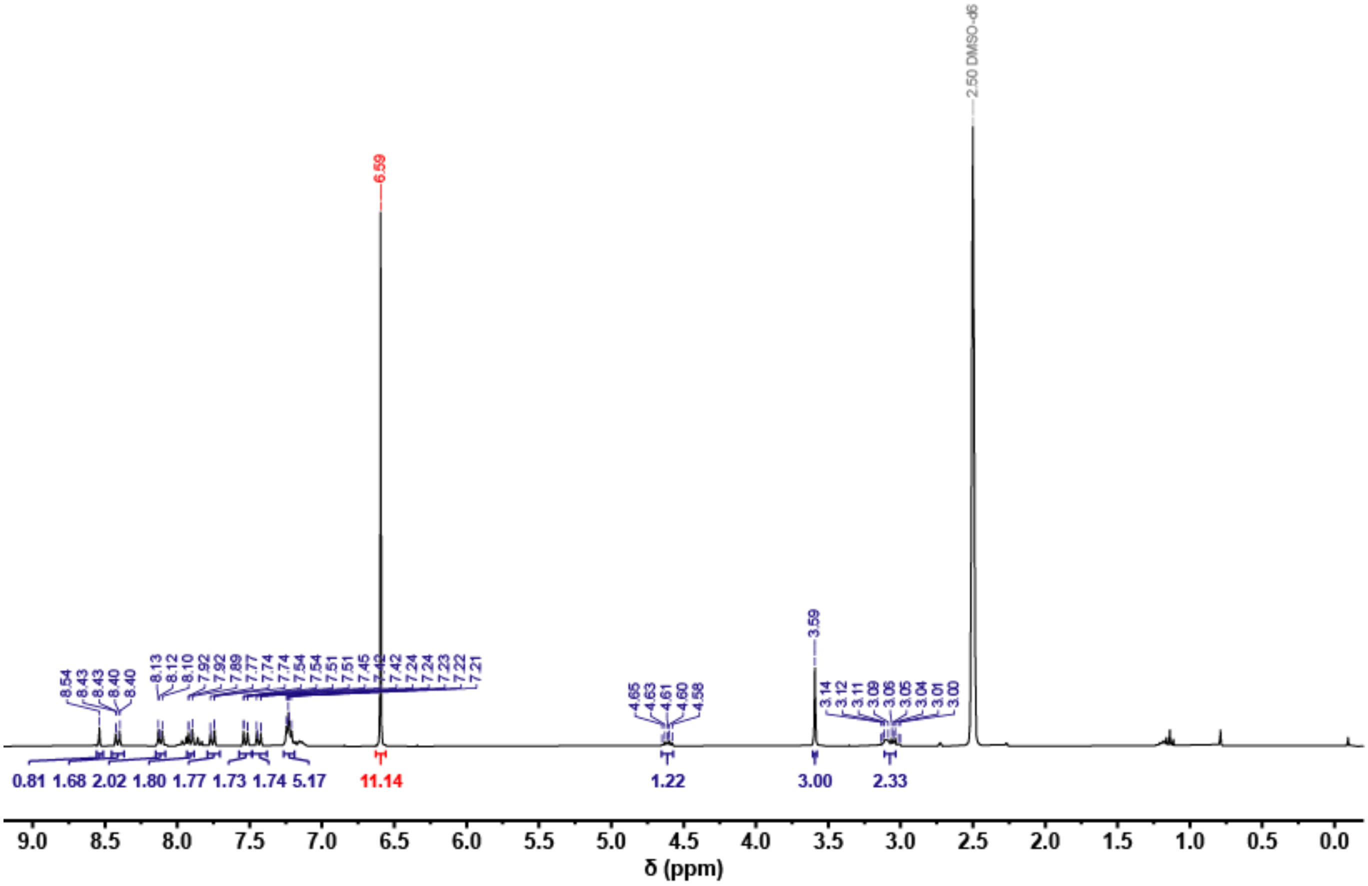

Figure S67. ^{1}H NMR quantification spectrum of UiO-68-PZDC-(*L*)-Phe. The signal in red corresponds to fumaric acid. Signals in blue correspond to the modified linker: ^{1}H NMR (300 MHz, DMSO): δ_H 8.54 (s, 1H), 8.45 – 8.38 (m, 2H), 8.14 – 8.09 (m, 2H), 7.93 – 7.89 (m, 2H), 7.80 – 7.73 (m, 2H), 7.59 – 7.50 (m, 2H), 7.48 – 7.40 (m, 2H), 7.27 – 7.17 (m, 5H), 4.66 – 4.57 (m, 1H), 3.59 (s, 3H), 3.15 – 3.00 (m, 2H).

Digested UiO-68-PZDC-(*L*)-His

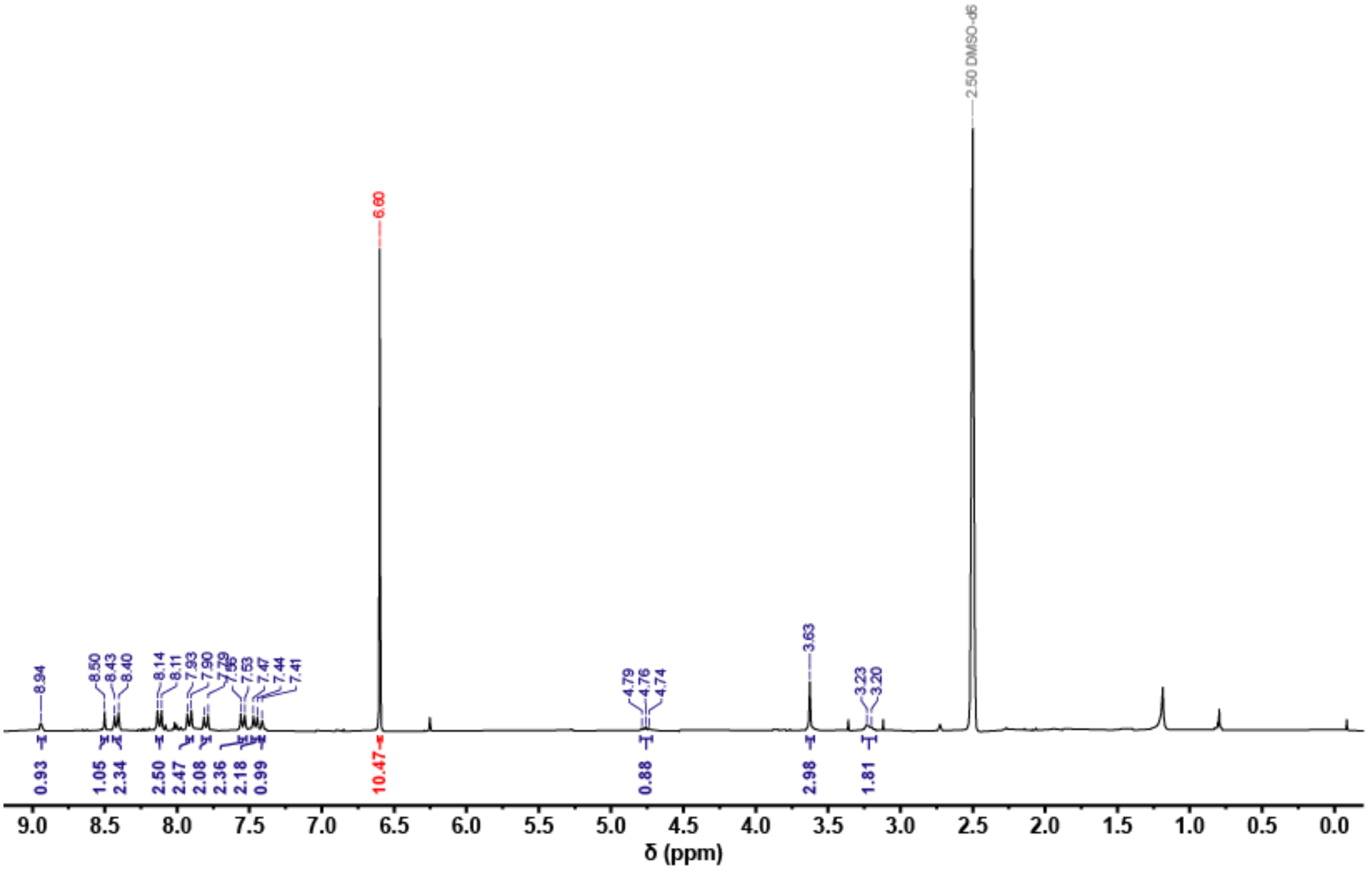

Figure S68. ^{1}H NMR quantification spectrum of UiO-68-PZDC-(*L*)-His. The signal in red corresponds to fumaric acid. Signals in blue correspond to the modified linker: ^{1}H NMR (300 MHz, DMSO): δ_H 8.94 (s, 1H), 8.50 (s, 1H), 8.44 – 8.39 (m, 2H), 8.13 – 8.09 (m, 2H), 7.96 – 7.85 (m, 2H), 7.82 – 7.74 (m, 2H), 7.59 – 7.49 (m, 2H), 7.50 – 7.42 (m, 2H), 7.41 (s, 1H), 4.90 – 4.67 (m, 1H), 3.63 (s, 3H), 3.28 – 3.20 (m, 2H).

S.8. REFERENCES

(1) Coelho, A. A. TOPAS and TOPAS-Academic: An Optimization Program Integrating Computer Algebra and Crystallographic Objects Written in C++. *J. Appl. Crystallogr.* **2018**, *51* (1), 210–218.

(2) Lerma-Berlanga, B.; Ganivet, C. R.; Almora-Barrios, N.; Tatay, S.; Peng, Y.; Albero, J.; Fabelo, O.; González-Platas, J.; García, H.; Padial, N. M.; Martí-Gastaldo, C. Effect of Linker Distribution in the Photocatalytic Activity of Multivariate Mesoporous Crystals. *J. Am. Chem. Soc.* **2021**, *143* (4), 1798–1806.

(3) Schnell, S. D.; González, J. A.; Sklyaruk, J.; Linden, A.; Gademann, K. Boron Trifluoride-Mediated Cycloaddition of 3-Bromotetrazine and Silyl Enol Ethers: Synthesis of 3-Bromo-Pyridazines. *J. Org. Chem.* **2021**, *86* (17), 12008–12023.

(4) Lerma‐Berlanga, B.; Ganivet, C. R.; Almora‐Barrios, N.; Vismara, R.; Navarro, J. A. R.; Tatay, S.; Padial, N. M.; Martí‐Gastaldo, C. Tetrazine Linkers as Plug‐and‐Play Tags for General Metal‐Organic Framework Functionalization and C_{60} Conjugation. *Angew. Chem. Int. Ed.* **2022**, *61* (41), e202208139.

(5) Howarth, A. J.; Peters, A. W.; Vermeulen, N. A.; Wang, T. C.; Hupp, J. T.; Farha, O. K. Best Practices for the Synthesis, Activation, and Characterization of Metal–Organic Frameworks. *Chem. Mater.* **2017**, *29* (1), 26–39.

(6) Bueno-Perez, R.; Martin-Calvo, A.; Gómez-Álvarez, P.; Gutiérrez-Sevillano, J. J.; Merkling, P. J.; Vlugt, T. J. H.; Erp, T. S. van; Dubbeldam, D.; Calero, S. Enantioselective Adsorption of Ibuprofen and Lysine in Metal–Organic Frameworks. *Chem. Commun.* **2014**, *50* (74), 10849–10852.

(7) Lei, M.; Wang, X.; Zhang, T.; Shi, Y.; Wen, J.; Zhang, Q. Homochiral Eu3+@MOF Composite for the Enantioselective Detection and Separation of (*R*/*S*)-Ornidazole. *Inorg. Chem.* **2022**, *61* (18), 6764–6772.

(8) Abbas, A.; Wang, Z.-X.; Li, Z.; Jiang, H.; Liu, Y.; Cui, Y. Enantioselective Separation over a Chiral Biphenol-Based Metal–Organic Framework. *Inorg. Chem.* **2018**, *57* (15), 8697 8700.

(9) Navarro-Sánchez, J.; Argente-García, A. I.; Moliner-Martínez, Y.; Roca-Sanjuán, D.; Antypov, D.; Campíns-Falcó, P.; Rosseinsky, M. J.; Martí-Gastaldo, C. Peptide Metal–Organic Frameworks for Enantioselective Separation of Chiral Drugs. *J. Am. Chem. Soc.* **2017**, *139* (12), 4294–4297.

(10) Chen, T.; Li, H.; Shi, X.; Imbrogno, J.; Zhao, D. Robust Homochiral Polycrystalline Metal–Organic Framework Membranes for High-Performance Enantioselective Separation. *J. Am. Chem. Soc.* **2024**, *146* (21), 14433–14438.

(11) Corella-Ochoa, M. N.; Tapia, J. B.; Rubin, H. N.; Lillo, V.; González-Cobos, J.; Núñez-Rico, J. L.; Balestra, S. R. G.; Almora-Barrios, N.; Lledós, M.; Güell-Bara, A.; Cabezas-Giménez, J.; Escudero-Adán, E. C.; Vidal-Ferran, A.; Calero, S.; Reynolds, M.; Martí-Gastaldo, C.; Galán-Mascarós, J. R. Homochiral Metal–Organic Frameworks for Enantioselective Separations in Liquid Chromatography. *J. Am. Chem. Soc.* **2019**, *141* (36), 14306–14316.

(12) Tang, H.; Yang, K.; Wang, K.-Y.; Meng, Q.; Wu, F.; fang, Y.; Wu, X.; Li, Y.; Zhang, W.; Luo, Y.; Zhu, C.; Zhou, H.-C. Engineering a Homochiral Metal–Organic Framework Based on an Amino Acid for Enantioselective Separation. *Chem. Commun.* **2020**, *56* (63), 9016–9019.

(13) Jiang, H.; Yang, K.; Zhao, X.; Zhang, W.; Liu, Y.; Jiang, J.; Cui, Y. Highly Stable Zr(IV)-Based Metal–Organic Frameworks for Chiral Separation in Reversed-Phase Liquid Chromatography. *J. Am. Chem. Soc.* **2020**, *143* (1), 390–398.

(14) Abbas, A.; Wang, Z.-X.; Li, Z.; Jiang, H.; Liu, Y.; Cui, Y. Enantioselective Separation over a Chiral Biphenol-Based Metal–Organic Framework. *Inorg. Chem.* **2018**, *57* (15), 8697 8700.

(15) Kresse, G.; Furthmüller, J. Efficiency of Ab-Initio Total Energy Calculations for Metals and Semiconductors Using a Plane-Wave Basis Set. *Comput. Mater. Sci.* **1996**, *6* (1), 15–50.

(16) Kresse, G.; Furthmüller, J. Efficient Iterative Schemes for Ab Initio Total-Energy Calculations Using a Plane-Wave Basis Set. *Phys. Rev. B.* **1996**, *54* (16), 11169–11186.

(17) Perdew, J. P.; Burke, K.; Ernzerhof, M. Generalized Gradient Approximation Made Simple. *Phys. Rev. Lett.* **1996**, *77* (18), 3865–3868.

(18) Grimme, S.; Antony, J.; Ehrlich, S.; Krieg, H. A Consistent and Accurate Ab Initio Parametrization of Density Functional Dispersion Correction (DFT-D) for the 94 Elements H-Pu. *J. Chem. Phys.* **2010**, *132* (15), 154104.

(19) Bučko, T.; Hafner, J.; Lebègue, S.; Ángyán, J. G. Improved Description of the Structure of Molecular and Layered Crystals: Ab Initio DFT Calculations with van Der Waals Corrections. *J. Phys. Chem. A.* **2010**, *114* (43), 11814–11824.

(20) Kresse, G.; Joubert, D. From Ultrasoft Pseudopotentials to the Projector Augmented-Wave Method. *Phys. Rev. B.* **1998**, *59* (3), 1758–1775.

(21) Gale, J. D.; LeBlanc, L. M.; Spackman, P. R.; Silvestri, A.; Raiteri, P. A Universal Force Field for Materials, Periodic GFN-FF: Implementation and Examination. *J. Chem. Theory Comput.* **2021**, *17* (12), 7827–7849.

(22) Spicher, S.; Grimme, S. Robust Atomistic Modeling of Materials, Organometallic, and Biochemical Systems. *Angew. Chem. Int. Ed.* **2020**, *59* (36), 15665–15673.

(23) Gale, J. D. GULP: A Computer Program for the Symmetry-Adapted Simulation of Solids. *J Chem Soc, Faraday Trans.* **1997**, *93* (4), 629–637.

(24) Yuan, C.; Wang, Z.; Xiong, W.; Huang, Z.; Lai, Y.; Fu, S.; Dong, J.; Duan, A.; Hou, X.; Yuan, L.-M.; Cui, Y. Cyclodextrin Incorporation into Covalent Organic Frameworks Enables Extensive Liquid and Gas Chromatographic Enantioseparations. *J. Am. Chem. Soc.* **2023**, *145* (34), 18956–18967.

(25) Dybtsev, D. N.; Yutkin, M. P.; Samsonenko, D. G.; Fedin, V. P.; Nuzhdin, A. L.; Bezrukov, A. A.; Bryliakov, K. P.; Talsi, E. P.; Belosludov, R. V.; Mizuseki, H.; Kawazoe, Y.; Subbotin, O. S.; Belosludov, V. R. Modular, Homochiral, Porous Coordination Polymers: Rational Design,

Enantioselective Guest Exchange Sorption and Ab Initio Calculations of Host–Guest Interactions. *Chem. Eur. J.* **2010**, *16* (34), 10348–10356.

(26) Suh, K.; Yutkin, M. P.; Dybtsev, D. N.; Fedin, V. P.; Kim, K. Enantioselective Sorption of Alcohols in a Homochiral Metal–Organic Framework. *Chem. Commun.* **2011**, *48* (4), 513–515.

(27) Bao, X.; Broadbelt, L. J.; Snurr, R. Q. Elucidation of Consistent Enantioselectivity for a Homologous Series of Chiral Compounds in Homochiral Metal–Organic Frameworks. *Phys. Chem. Chem. Phys.* **2010**, *12* (24), 6466–6473.

(28) Li, M.; Zhang, L.; Wu, B.; Hong, M. High-Enantioselectivity Adsorption Separation of Racemic Mandelic Acid and Methyl Mandelate by Robust Chiral UiO-68-Type Zr-MOFs. *Inorg. Chem.* **2024**, *63* (1), 381–389.

(29) Li, M.; Yuan, D.; Wu, B.; Hong, M. Engineering UiO-68-Typed Homochiral Metal–Organic Frameworks for the Enantiomeric Separation of Fmoc-AAs and Mechanism Study. *ACS Appl. Mater. Interfaces.* **2023**, *15* (18), 22241–22250.

(30) Peng, Y.; Gong, T.; Zhang, K.; Lin, X.; Liu, Y.; Jiang, J.; Cui, Y. Engineering Chiral Porous Metal-Organic Frameworks for Enantioselective Adsorption and Separation. *Nat. Commun.* **2014**, *5* (1), 4406.

(31) Gilli, G.; Gilli, P. *The Nature of the Hydrogen Bond*; Press, O. U., Ed.; 2009.

(32) Zhao, Y.; Li, J.; Gu, H.; Wei, D.; Xu, Y.; Fu, W.; Yu, Z. Conformational Preferences of π–π Stacking Between Ligand and Protein, Analysis Derived from Crystal Structure Data Geometric Preference of π–π Interaction. *Interdiscip. Sci.: Comput. Life Sci.* **2015**, *7* (3), 211–220.

(33) Johnson, E. R.; Keinan, S.; Mori-Sánchez, P.; Contreras-García, J.; Cohen, A. J.; Yang, W. Revealing Noncovalent Interactions. *J. Am. Chem. Soc.* **2010**, *132* (18), 6498–6506.

(34) Lu, T.; Chen, F. Multiwfn: A Multifunctional Wavefunction Analyzer. *J. Comput. Chem.* **2012**, *33* (5), 580–592.

(35) Chinchilla‐Garzón, C.; Galbiati, M.; Misturini, A.; Gimeno‐Fonquernie, P.; Almora‐Barrios, N.; Padial, N. M.; Martí‐Gastaldo, C. Structural Control of Photoconductivity in a Flexible Titanium‐Organic Framework. *Adv. Mater.* **2025**, e2412045.
